# Effectiveness of strategies to improve health-care provider practices in low-income and middle-income countries: a systematic review

**DOI:** 10.1016/S2214-109X(18)30398-X

**Published:** 2018-10-08

**Authors:** Alexander K Rowe, Samantha Y Rowe, David H Peters, Kathleen A Holloway, John Chalker, Dennis Ross-Degnan

**Affiliations:** a Malaria Branch, Division of Parasitic Diseases and Malaria, Center for Global Health, Centers for Disease Control and Prevention, Atlanta, GA, USA; b CDC Foundation, Atlanta, GA, USA; c Department of International Health, Johns Hopkins Bloomberg School of Public Health, Baltimore, MD, USA; d World Health Organization, Southeast Asia Regional Office, New Delhi, India; e International Institute of Health Management Research, Jaipur, India; f Institute of Development Studies, University of Sussex, Brighton, UK; g Pharmaceuticals & Health Technologies Group, Management Sciences for Health, Arlington, VA, USA; h Harvard Medical School and Harvard Pilgrim Health Care Institute, Boston, MA, USA

## Abstract

**Background:**

Inadequate health-care provider performance is a major challenge to the delivery of high-quality health care in low-income and middle-income countries (LMICs). The Health Care Provider Performance Review (HCPPR) is a comprehensive systematic review of strategies to improve health-care provider performance in LMICs.

**Methods:**

For this systematic review we searched 52 electronic databases for published studies and 58 document inventories for unpublished studies from the 1960s to 2016. Eligible study designs were controlled trials and interrupted time series. We only included strategy-versus-control group comparisons. We present results of improving health-care provider practice outcomes expressed as percentages (eg, percentage of patients treated correctly) or as continuous measures (eg, number of medicines prescribed per patient). Effect sizes were calculated as absolute percentage-point changes. The summary measure for each comparison was the median effect size (MES) for all primary outcomes. Strategy effectiveness was described with weighted medians of MES. This study is registered with PROSPERO, number CRD42016046154.

**Findings:**

We screened 216 477 citations and selected 670 reports from 337 studies of 118 strategies. Most strategies had multiple intervention components. For professional health-care providers (generally, facility-based health workers), the effects were near zero for only implementing a technology-based strategy (median MES 1·0 percentage points, IQR −2·8 to 9·9) or only providing printed information for health-care providers (1·4 percentage points, −4·8 to 6·2). For percentage outcomes, training or supervision alone typically had moderate effects (10·3–15·9 percentage points), whereas combining training and supervision had somewhat larger effects than use of either strategy alone (18·0–18·8 percentage points). Group problem solving alone showed large improvements in percentage outcomes (28·0–37·5 percentage points), but, when the strategy definition was broadened to include group problem solving alone or other strategy components, moderate effects were more typical (12·1 percentage points). Several multifaceted strategies had large effects, but multifaceted strategies were not always more effective than simpler ones. For lay health-care providers (generally, community health workers), the effect of training alone was small (2·4 percentage points). Strategies with larger effect sizes included community support plus health-care provider training (8·2–125·0 percentage points). Contextual and methodological heterogeneity made comparisons difficult, and most strategies had low quality evidence.

**Interpretation:**

The impact of strategies to improve health-care provider practices varied substantially, although some approaches were more consistently effective than others. The breadth of the HCPPR makes its results valuable to decision makers for informing the selection of strategies to improve health-care provider practices in LMICs. These results also emphasise the need for researchers to use better methods to study the effectiveness of interventions.

**Funding:**

Bill & Melinda Gates Foundation, CDC Foundation.

## Introduction

Health-care providers, including facility-based and community-based health workers, are essential for delivering high-quality health care. However, hundreds of studies have documented inadequate health-care provider performance in low-income and middle-income countries (LMICs).^1^, ^2^, ^3^, ^4^, ^5^, ^6^, ^7^, ^8^, ^9^

Numerous strategies to improve health-care provider performance have been tested in LMICs, and a summary of the evidence about successful approaches would be valuable for decision makers. Many systematic reviews have been done for specific sets of strategies or types of health services,^6^, ^10^, ^11^, ^12^, ^13^, ^14^, ^15^, ^16^, ^17^, ^18^, ^19^ but a common shortcoming of these studies is that they include only a limited range of strategies for improving health-care provider performance in LMICs. To answer the broad programmatic question about the most effective ways to improve health-care provider performance, all strategies would need to be compared. The Health Care Provider Performance Review (HCPPR) is designed to help fill this gap. The primary objective of the HCPPR is to assess the effectiveness and cost of all strategies to improve health-care provider performance outcomes in LMICs. Strategies in the HCPPR include components directly targeting individual health-care provider behaviour (eg, training and supervision) as well as broader, systems-level interventions to reform or strengthen areas such as health system financing, management, and infrastructure.

**Research in context**

**Evidence before this study**

We did not do a systematic review to establish the need for the Health Care Provider Performance Review (HCPPR). Before the HCPPR protocol was developed in 2005, we examined three landmark reviews of the effectiveness of multiple strategies to improve health-care provider performance and found important limitations. One study was not a systematic review, and it included studies from both high-income countries (HICs) and low-income and middle-income countries (LMICs). A second study was a systematic review, but the literature search from 1998 was outdated, and nearly all studies were from HICs. A third study was a systematic review of studies from LMICs, but the literature search from 1999 was outdated, and the review only included studies on improving antimicrobial use, which excluded many important aspects of health care. We also identified nine reviews of individual strategies. Although these reviews were important contributions to the literature, their shared limitation was that they each only evaluated a single strategy; and, taken together, they only covered a relatively narrow set of strategies.

**Added value of this study**

The evidence about the effectiveness of all strategies to improve health-care provider practices in LMICs (evaluated with reasonably good quality studies, and acknowledging that some studies were inevitably missed, which is a limitation of all reviews) has been, to the best of our knowledge, summarised for the first time with a single analytical approach in one systematic review. The public release of the review's database will allow others to do analyses that are tailored to the needs of specific health programmes (eg, for a particular geographical location, health condition, or health-care delivery setting).

**Implications of all the available evidence**

Results of the HCPPR support 14 guidance statements on improving health-care provider practices in LMICs. For example, the review identified strategies that tended to have large effect sizes (eg, group problem solving plus training), which programmes might consider using, as well as strategies that were generally ineffective (eg, only providing printed information to health-care providers), which health programmes might want to avoid. The review also found wide variability in the effects of nearly all strategies tested by multiple studies, which underscores the importance of monitoring the effect of any strategy. Future research should identify the attributes of commonly used strategies, such as training and supervision, that are associated with effectiveness; focus on a better understanding of how context influences strategy effectiveness; emphasise the use of robust and standardised methods; and include more rigorous studies of strategies to improve the performance of community health workers.

Health worker performance is a relatively broad construct that encompasses availability, clinical competence, responsiveness (providing patient-centred care), and productivity (or efficiency).^20^ Studies in the HCPPR use a multitude of outcomes that reflect health-care provider practices, patients' health outcomes, and other aspects of performance. Here, we present results of the effectiveness of strategies to improve health-care provider practices expressed as percentages (eg, percentage of patients diagnosed or treated correctly) or as continuous measures (eg, number of medicines prescribed per patient). Improving health-care provider practices is important for programmes and the patients they serve, as well as being relevant for meeting the UN Sustainable Development Goal of achieving universal health coverage.^1^ Improvements in health-care provider practices have been found to be highly correlated with improvements in patients' health outcomes.^21^ This outcome category also had the largest number of studies in the HCPPR.

## Methods

### Search strategy and selection criteria

The HCPPR is a systematic review that includes all elements recommended by the PRISMA guidelines.^22^ The PRISMA checklist and the review protocol are in appendix 1 (pp 2–25).

We included published and unpublished studies from LMICs that quantitatively evaluated strategies to improve health-care provider performance (for detailed inclusion criteria see appendix 1, pp 8–10). Eligible strategies had to include at least one intervention component that could plausibly affect performance. Health-care providers were broadly defined as hospital-based, clinic-based, or community-based health workers; pharmacists and dispensers; and shopkeepers and informal vendors who sell medicines. Studies could include multiple health-care provider types and multiple service delivery settings (eg, hospital inpatient wards and hospital-based outpatient clinics). Eligible study designs comprised pre-intervention versus post-intervention studies with a randomised or non-randomised comparison group, post-intervention only studies with a randomised comparison group, and interrupted time series (ITS) with at least three baseline and follow-up measures. We included studies on any health condition, in any language, and from public and private sector settings. We only included results for primary study outcomes. For this report, we only included health-care provider practice outcomes, which are health-care provider behaviours such as patient assessment, diagnosis, and treatment (full list provided in appendix 1, pp 83, 84). This report only includes strategy-versus-control group comparisons (head-to-head comparisons will be analysed separately). A comparison denotes an examination or analysis of two study groups (or a study group and its historical control in ITS studies). A study (eg, with three groups) could have several comparisons. For percentage outcomes, we excluded effect sizes with baselines of 95% or greater, as there was little room for improvement.

We searched 52 electronic databases for published studies and 58 document inventories for unpublished studies from the 1960s to 2016. Literature searches were done from 2006 to 2008 (Rowe SY, unpublished) and from October, 2015, to May, 2016 (appendix 1, pp 26–37). We also screened personal libraries, asked colleagues for unpublished studies, and hand-searched bibliographies from previous reviews.

Titles and abstracts were screened to identify relevant reports. If this screening was insufficient for establishing eligibility, the report's full text was reviewed. Data were abstracted independently by two investigators or research assistants by use of a standardised form. Discrepancies were resolved through discussion. Data were entered into a computer database (Microsoft Access, Microsoft Inc, Professional Plus 2016). Data elements included study setting and timing, health-care provider type, improvement strategies, study design, sample size, outcomes, effect sizes, risk of bias domains, and economic evaluations. Study investigators were queried about details not available in the study reports.

### Time trends in study attributes

We defined the time for a given study as the publication year. If study results were presented in multiple reports, we analysed the year that the first report identified by the HCPPR was publicly available. Time trends for dichotomous study attributes were assessed by logistic regression. Time trends in the number of studies per year were assessed with a Poisson regression model, or with a negative binomial regression model to address over-dispersion. Goodness-of-fit was assessed with a χ^2^ test of deviance, with a p value greater than 0·05 indicating adequate model fit. For analyses of the number of studies per year, studies published after 2015 were excluded as such studies did not represent all research done after that time because of the literature search end date of May, 2016.

### Definition of strategy groups

To define mutually exclusive strategy groups, we coded the presence of 207 strategy components for each study group exposed to an improvement strategy and grouped them into 13 component categories (panel 1; appendix 1, pp 39–44). A unique strategy was any unique combination of the 13 component categories. Placebo strategy components were analysed with control groups, which received no new intervention. After ascertaining that there was no meaningful difference between results of low-intensity and high-intensity training (appendix 1, pp 88, 93), these results were combined into a single “any training” category.

Panel 1 **Definitions of strategy component categories**

**Community support**

Examples include community health education or social marketing of health services.

**Patient support**

Examples include patient health education via printed materials or home visits.

**Strengthening infrastructure**

Examples include provision of medicines or implementation of an improved data collection system.

**Health-care provider-directed financial incentives**

Examples include performance-based payments.

**Health system financing and other incentives**

Examples include social health insurance or reducing a consultation fee.

**Regulation and governance**

Examples include accreditation or introducing standard drug quality requirements.

**Group problem solving**

Examples include collaborative improvement or group problem solving with or without formal teams.

**Supervision**

Examples include improving routine supervision, benchmarking, or audit with feedback.

**Other management techniques**

These include techniques that do not include group problem solving and supervision (which are separate component categories), such as health-care provider self-assessment or health-care provider group process that is neither training nor group problem solving.

High-intensity training*

Training with a duration greater than 5 days (or ongoing training) and at least one interactive educational method (ie, clinical practice, role play, or interactive sessions). This category includes academic detailing (ie, one-on-one training by an opinion leader).

Low-intensity training*

Any training that was not categorised as high-intensity training (above). This category includes informal education of health-care providers by their peers.

**Printed information (including job aids) for health-care providers**

This category refers to information that is not an integral part of another component, such as a pamphlet. Other strategy components (especially training) often include printed information for health-care providers; in these cases, the printed information was not considered a separate component.

**Information and communication technology for health-care providers**

This category includes mHealth and eHealth. Examples include computerised decision aids or text message reminders sent to health-care providers' phones.

See appendix 1 for further details (pp 39–44).

### Assessment of risk of bias, quality of evidence, and publication bias

Our study-level risk of bias assessment was based on guidance from the Cochrane Effective Practice and Organisation of Care Group;^23^ the categories were low, moderate, high, and very high (appendix 1, pp 45–47). Our strategy-level quality of evidence (QOE) assessment used the Grading of Recommendations Assessment, Development, and Evaluation (GRADE) system (appendix 1, pp 48–51),^24^ which has four categories: high, moderate, low, and very low. To identify publication bias, we examined funnel plots and did Egger's test for strategies tested by at least ten comparisons.^25^

### Estimation of effect sizes

The primary outcome measure was the effect size, which was defined as an absolute percentage-point difference and calculated such that positive values indicate improvement. For study outcomes that decreased to indicate improvement (eg, percentage of patients receiving unnecessary treatments), we multiplied effect sizes by −1. Effect sizes for percentage and continuous outcomes were calculated differently (see below) and analysed separately.

For non-ITS studies, effect sizes were based on the baseline value closest in time to the beginning of the strategy and the follow-up value furthest in time from the beginning of the strategy. In non-ITS studies, for outcomes that were dichotomous, percentages, or bounded continuous outcomes that could be converted to percentages (eg, performance score ranging from 0 to 12), the effect size was calculated with equation 1:

$$\text{Effect}\;\text{size}={\left(\text{follow}-\text{up}-\text{baseline}\right)}_{\text{intervention}}-{\left(\text{follow}-\text{up}-\text{baseline}\right)}_{\text{control}}$$

In non-ITS studies, for unbounded continuous outcomes, the effect size was calculated with equation 2.

$$\begin{array}{l} \text{Effect}\;\text{size}=100\%\times \\ \{{\left(\frac{\text{follow}-\text{up}-\text{baseline}}{\text{baseline}}\right)}_{\text{intervention}}-\\ {\left(\frac{\text{follow}-\text{up}-\text{baseline}}{\text{baseline}}\right)}_{\text{control}}\} \end{array}$$

If the baseline value for either the intervention or control group equalled zero, the effect size was undefined and thus excluded.

For ITS studies, segmented linear regression modelling^26^ was done to estimate a summary effect size that incorporated both the level and trend effects (appendix 1, pp 52–56). For example, for percentage outcomes from a single-arm ITS study, the summary effect size was the outcome level at the midpoint of the follow-up period as predicted by the regression model minus a predicted counterfactual value that equalled the outcome level based on the pre-intervention trend extended to the midpoint of the follow-up period.

We adjusted effect sizes for contextual and methodological factors that might have differed among effect sizes and strategy groups (appendix 1, pp 58–61). These factors (eg, baseline performance level) were identified with random-effects linear regression modelling. The purpose of the adjustment was to reduce bias when comparing strategies. As the model includes dummy variables for the strategy component categories, the results also estimate the marginal effect of adding a strategy component category.

### Estimation of strategy effectiveness

To estimate strategy effectiveness, the effect size for each comparison was defined as the median of all effect sizes within the comparison.

We stratified results by whether the primary focus of the comparison was on improving performance of professional health-care providers, who typically work in health facilities (eg, physicians, nurses, and midwives), or on improving lay or community health worker performance. We also stratified results by whether or not the comparison was an equivalency comparison with a gold standard control group (for which an effect size close to zero would be considered a successful result).

To compare the effectiveness of different strategies in a way that accounts for or reduces bias from outliers, small numbers of studies per strategy, unequal sample sizes, and methodological and contextual differences among the studies, we used a primary and secondary analysis (described below), each with advantages and limitations. Unless otherwise specified, all analyses were done with SAS version 9.4.

### Primary analysis

In the primary analysis, each study comparison was summarised with a median effect size (MES), and the effectiveness of each strategy was described with a median of MES values (IQR, range). Medians for strategies tested by five or more study comparisons were weighted, with weights equal to 1 plus the natural logarithm of the number of health-care providers or (if number of health-care providers was not reported) the number of service provision sites (eg, number of health facilities) or (if neither the number of health-care providers nor the number of service provision sites were available) the number of administrative units (eg, districts). Strategy groups tested by at least three study comparisons (ie, at least three comparisons with percentage outcomes or at least three comparisons with continuous outcomes) were considered to have enough evidence to form generalisable estimates, with increasing caution in interpretation as the minimum of three comparisons was approached. Strategies tested by fewer than three study comparisons per outcome group were interpreted separately.

Four sensitivity analyses were done to examine the influence of study bias, limited numbers of comparisons, different economic and health facility settings, and the effects of weighting and adjustment (see appendix 1 for details, p 62). One sensitivity analysis involved broadening the strategy definition to increase the contextual and implementation diversity of the studies that tested the strategy (eg, “information and communication technology [ICT] only” was broadened to “ICT with or without other strategy components”). This approach is useful when a strategy is tested by few studies, and effectiveness is unusually large or small.

### Secondary analysis

For percentage outcomes only, the secondary analysis involved summarising comparisons with an MES, as described above, and then a standard random-effects meta-analysis (which was an a-priori methodological choice) was used to estimate the weighted mean MES and 95% CI of each strategy.^27^ To do a meta-analysis, we required standard errors for effect sizes that accounted for any correlations in the data (eg, patients “clustered” within health facilities). We did not use standard errors from study reports when investigators did not account for data correlations. Instead, we applied some conservative assumptions about the correlations (eg, an intraclass correlation of 0·4) to the sample size information from the studies, and we calculated conservative estimates of standard errors (appendix 1, pp 63–74). The secondary analysis focuses only on percentage outcomes because in the initial version of the HCPPR, sample size information needed for standard error calculations was only available for percentage outcomes (this information was not in our initial data abstraction form and thus required a second round of data abstraction).

### Role of the funding source

The funders of the study had no role in study design and conduct, data collection, data management, data analysis, data interpretation, or writing of the report. AKR, the corresponding author, had full access to all the reports included in the review and had final responsibility for the decision to submit for publication.

## Results

**Figure 1:**
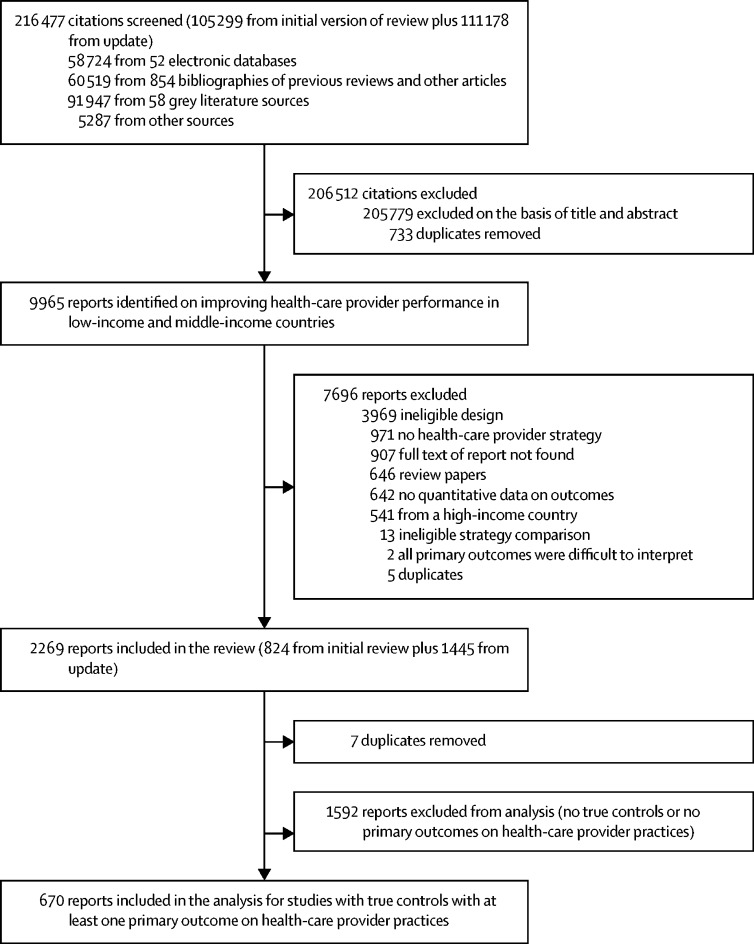
Study selection

We screened 216 477 citations (figure 1). After excluding 205 779 citations and 733 duplicates, we found 9965 reports on improving health-care provider performance in LMICs. Of these reports, 7696 were excluded, primarily for ineligible study designs. Of the remaining 2269 eligible reports, 670 were from studies with at least one health-care provider practice outcome and a true control group (or historical control from single-arm ITS studies). These reports, which are the focus of this Article, presented results for 337 studies.

The 337 studies comprised 381 comparisons of 118 strategies (table 1). Of the four strategies tested by the minimum number of studies (ten) to evaluate publication bias, we found evidence of publication bias for one strategy: supervision plus training (asymmetric funnel plot and Egger's test p=0·0017).

**Table 1:** Number of studies, strategies, comparisons, effect sizes, and distribution of median effect size values for each health-care provider group, stratified by outcome scale

	**Health-care provider practice outcome scale**		**Totals for percentage and continuous outcomes combined**
	Percentage outcomes	Continuous outcomes	
Professional health-care providers (eg, physicians, nurses, midwives, and other health-care providers who typically work in health facilities); lay health workers might be included, but they are not the primary focus of the study	269 studies*, 101 strategies*, 309 comparisons*, and 1297 effect sizes*; median MES 11·5 percentage points†‡ (5·1 to 24·6; −19·9 to 77·8)	96 studies§, 53 strategies§, 106 comparisons§, and 182 effect sizes§; median MES 11·8 percentage points†¶ (−2·2 to 56·9; −90·4 to 615·5)	313 studies, 112 strategies, 356 comparisons, and 1479 effect sizes
Lay health workers are the predominant type of health-care provider in the study	18 studies, 14 strategies, 19 comparisons, and 189 effect sizes; median MES 8·2 percentage points† (4·9 to 12·4; −6·1 to 68·4)	9 studies‖, 9 strategies‖, 9 comparisons‖, and 20 effect sizes‖; median MES 40·2 percentage points†** (23·3 to 99·0; 5·3 to 125·0)	24 studies, 18 strategies, 25 comparisons, and 209 effect sizes
Totals for both health-care provider groups combined	287 studies, 106 strategies, 328 comparisons, and 1486 effect sizes	105 studies, 58 strategies, 115 comparisons, and 202 effect sizes	337 studies, 118 strategies, 381 comparisons, and 1688 effect sizes

MES=median effect size.

* Includes four effect sizes from two comparisons from two studies that were equivalency comparisons with a gold standard control group (equivalency comparisons).

† Median (IQR; range) of the MES per comparison. Medians for cells with five or more study comparisons were weighted (see Methods). For percentage outcomes among studies for professional health-care providers, MES was based on effect sizes adjusted for baseline performance level, public health facility setting only, and study done in Asia. No adjustment for percentage outcomes among studies for lay health-care providers or continuous outcomes.

‡ Results are for the 307 comparisons that did not involve an equivalency comparison.

§ Includes six effect sizes from two comparisons from two studies that were equivalency comparisons.

¶ Results are for the 104 comparisons that did not involve an equivalency comparison.

‖ Includes one effect size from one comparison in one study that was an equivalency comparison.

** Results are for the eight comparisons that did not involve an equivalency comparison.

The 337 studies represented a diversity of methods, geographical settings, health-care provider types, work environments, and health conditions. Appendix 1 (pp 77–82) summarises study attributes in aggregate, and appendix 2 contains citations and details of individual studies. The studies were from 64 countries, with 133 (39·5%) from low-income countries (appendix 1, p 77). 284 (84·2%) studies were published in 2000 or later. Data on strategy costs or other economic evaluations were available from 125 (37·1%) studies. The studies tested 118 unique strategies (table 1), most with multiple intervention components. Risk of bias was low for 54 (16·0%) studies, moderate for 84 (24·9%), high for 98 (29·1%), and very high for 101 (30·0%). 284 (84·3%) studies had at least one study report published in a scientific journal, and there was no association between risk of bias and publication status (p=0·11; appendix 1, p 80). Among 317 studies for which study duration could be obtained, follow-up times were often short: 1–3 months in 114 (36·0%) studies, 4–9 months in 107 (33·7%) studies, and 10–73 months in 96 (30·3%) studies (appendix 1, p 161). We observed wide heterogeneity in outcomes, with nearly every study using a unique set.

**Figure 2:**
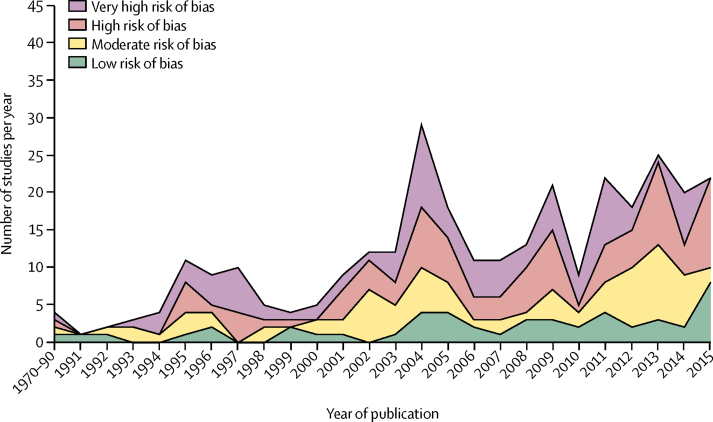
Number and risk of bias of studies with acceptable research designs* over time *Study designs eligible for the review included pre-intervention versus post-intervention studies with a randomised or non-randomised comparison group, post-intervention only studies with a randomised comparison group, and interrupted time series with at least three datapoints before and after the intervention.

The number of studies increased significantly from the 1970s to 2010s (figure 2; appendix 1, pp 86, 87). Research growth was so substantial that the number of studies per year significantly increased for all study categories, including study quality. Over time, studies were significantly more likely to be from upper-middle-income countries and Africa, and more likely to be published in a scientific journal. The proportion of studies with a low or moderate risk of bias did not significantly change over time.

For percentage outcomes, three contextual factors were significantly associated with effect size after adjusting for strategy component categories: baseline performance, settings with only public health facilities, and studies from Asia (table 2). For every 1 percentage-point increase in baseline performance, effect sizes decreased by 0·2 percentage points, on average, regardless of the strategy to improve performance (appendix 1, p 162). When the study setting comprised only public health facilities, effect sizes were 6·8 percentage points higher, on average. Effect sizes in Asian studies were, on average, 5·3 percentage points lower than in other continents. We therefore adjusted effect sizes for these three factors. The adjusted effect sizes reflect a partly standardised study context in which baseline performance is 40·1% (ie, mean for all effect sizes from percentage outcomes), about half (54·5%) of service provision sites are public health facilities and half are other settings (eg, private facilities and community settings), and 43·1% of settings are in Asia. Among the many factors not significantly associated with effect size, we present one that is often discussed in the literature: the number of components in the strategy (appendix 1, pp 163, 164).^15^, ^18^, ^28^ For continuous outcomes, the regression models seemed to be unstable, probably because of smaller sample sizes and a wide range of effect size values with influential outliers. Similarly, the small sample size of studies focused predominantly on lay or community health workers was not sufficient to support multivariable modelling. We decided not to proceed further with modelling for continuous outcomes or for outcomes from studies focused predominantly on lay or community health workers; thus, no adjustments were made for their effect sizes.

**Table 2:** Model to estimate strategy component effectiveness and identify contextual factors associated with effect size for practice outcomes expressed as a percentage from studies of professional health-care providers

		**Parameter estimate (95% CI)**	p value*
Intercept		7·6 (2·1 to 13·1)	0·01
Dummy variables† that each code for a strategy component category			
	Community support	0·3 (−5·0 to 5·6)	0·92
	Patient support	−3·6 (−9·5 to 2·2)	0·22
	Strengthening infrastructure	−1·2 (−7·2 to 4·9)	0·71
	Health-care provider-directed financial incentives	6·1 (−6·5 to 18·6)	0·34
	Health system financing and other incentives	2·5 (−3·4 to 8·5)	0·40
	Regulation and governance	2·7 (−4·3 to 9·7)	0·45
	Group problem solving	13·6 (5·7 to 21·6)	0·001
	Supervision	1·0 (−1·8 to 3·9)	0·48
	Other management techniques	3·3 (−1·8 to 8·5)	0·21
	Any training	6·4 (1·5 to 11·2)	0·01
	Printed information or job aid for health-care providers	−1·0 (−6·6 to 4·6)	0·72
	Information and communication technology for health-care providers	−2·4 (−8·6 to 3·8)	0·44
Contextual factors‡ for effect size adjustment (all mean-centred)			
	Baseline performance level	−0·2 (−0·2 to −0·1)	<0·0001
	Public health facility setting only (*vs* other settings)	6·8 (2·6 to 10·9)	0·002
	Country was in Asia	−5·3 (−9·2 to −1·4)	0·01

* Based on score statistics for type 3 tests of fixed effects, which tend to give conservative estimates. The one exception is the p value of the intercept, based on the *t* test, which tends to give less conservative estimates. This is the only p value provided in the SAS output for the intercept. The conclusion of the test (significant or not, based on a 0·05 cutoff) from the two sets of p values (*t* test *vs* type 3 test) always agreed.

† Dichotomous variable with a value of one if the strategy included a component from a given strategy component category (eg, training), otherwise the variable has a value of zero. The parameter estimate is the mean effect of the strategy component category, adjusted for other components in the strategy and contextual factors in the model.

‡ Baseline performance is a continuous variable, and public health facility setting only and Asian country are dichotomous variables. The adjusted R-square of the model was 0·05567 without contextual factors and 0·2155 with contextual factors.

Among studies of professional health-care providers, for percentage outcomes, the median MES across all 307 study comparisons (excluding equivalency comparisons) was an improvement of 11·5 percentage points (IQR 5·1 to 24·6; range −19·9 to 77·8; table 1). For continuous outcomes, the median MES across all 104 study comparisons (excluding equivalency comparisons) was an improvement of 11·8 percentage points (IQR −2·2 to 56·9; range −90·4 to 615·5), highlighting that extreme results can occur with effect sizes based on relative changes. Among studies predominantly done with lay or community health-care providers, the median MES for percentage outcomes was 8·2 percentage points and that for continuous outcomes was 40·2 percentage points (table 1).

In table 2, the parameter estimate for a given strategy component category dummy variable is the mean effect of strategies with the component category minus the mean effect of strategies without the component category, adjusted for all other factors in the model. Two strategy component categories were associated with greatest effectiveness: any training (marginal effect size of 6·4 percentage points) and group problem solving (marginal effect size of 13·6 percentage points). These findings suggest that training health-care providers and group problem solving might be useful components in any strategy.

The effectiveness of specific strategies is shown for non-equivalency studies. The four equivalency studies are presented in appendix 1 (pp 159, 160). For brevity, we use the term “effect” to denote strategy effectiveness in terms of the median MES. As qualitative descriptors of effect, “small effects” corresponds to improvements of less than 5 percentage points, “modest” corresponds to about 5–10 percentage points, “moderate” corresponds to about 10–20 percentage points, “large” corresponds to about 20–30 percentage points, and “very large” corresponds to greater than 30 percentage points.

Among 353 comparisons from 310 studies of professional health-care providers, we identified 111 unique strategies (table 3; appendix 1, pp 111–23). Among the 101 strategies tested with percentage outcomes, the GRADE-based QOE on effectiveness was high for two (2·0%) strategies, moderate for 20 (19·8%), low for 26 (25·7%), and very low for 53 (52·5%). Among the 51 strategies tested with continuous outcomes, the corresponding QOE proportions were high for two (3·9%), moderate for ten (19·6%), low for 15 (29·4%), and very low for 24 (47·1%). Most strategies (88 [79·3%] of 111) were tested by fewer than three study comparisons for both outcome types (ie, percentage and continuous outcomes; appendix 1, pp 111–23). Thus, the generalisability of these results is limited.

**Table 3:** Effectiveness of strategies to improve health-care provider performance for studies with at least one practice outcome

	**Practice outcomes expressed as a percentage**				**Continuous practice outcomes**			
	Number of study comparisons (studies with low or moderate risk of bias)	Number of countries studied	Median MES based on adjusted effect sizes* (IQR; range)	GRADE quality of evidence	Number of study comparisons (studies with low or moderate risk of bias)	Number of countries studied	Median MES based on unadjusted effect sizes (IQR; range)	GRADE quality of evidence
**Studies focused on professional health-care providers (descending order of effect size for percentage outcomes**†**)**								
Community support plus strengthening infrastructure plus supervision plus other management techniques plus any training	0 (0)	0	NA	NA	3 (1)	3	76·1 (NA; 73·9 to 153·0)	Moderate
Strengthening infrastructure plus health system financing and other incentives plus supervision plus other management techniques plus any training	3 (1)	2	57·7 (NA; 4·4 to 58·7)	Moderate	0 (0)	0	NA	NA
Group problem solving plus any training	4 (1)	2	56·0 (40·9 to 68·6; 29·2 to 77·8)	Moderate	1 (1)	1	52·4	Moderate
Strengthening infrastructure plus supervision plus other management techniques plus any training	2 (2)	2	33·1 (NA; 29·4 to 36·7)	Low	4 (1)	4	183·2 (63·2 to 456·3; 56·9 to 615·5)	Moderate
Group problem solving only	12 (3)	10	28·0 (12·1 to 41·7; 5·5 to 61·2)	Low	4 (0)	3	−8·1 (−24·3 to 44·2; −28·2 to 84·1)	Low
Community support plus supervision plus any training	4 (2)	4	20·7 (7·5 to 24·3; −2·9 to 25·3)	Low	0 (0)	0	NA	NA
Other management techniques plus printed information or job aid for HCPs	2 (2)	2	18·2 (NA; 4·7 to 31·8)	Low	3 (3)	3	11·8 (NA; 0·3 to 16·5)	Moderate
Supervision plus any training	26 (11)	17	18·0 (6·0 to 25·2; −2·7 to 67·0)	Very low	8 (3)	5	11·1 (7·3 to 60·4; −16·3 to 101·1)	Low
Other management techniques only	4 (3)	3	16·5 (2·3 to 21·3; −11·1 to 25·3)	Moderate	0 (0)	0	NA	NA
Other management techniques plus any training	5 (1)	4	15·9 (2·8 to 23·9; −1·7 to 54·2)	Low	2 (0)	2	9·1 (NA; 8·3 to 9·9)	Very low
Community support plus any training	4 (0)	4	15·1 (9·0 to 25·0; 8·2 to 29·6)	Very low	1 (1)	1	4·5	Low
Supervision only	16 (8)	12	14·8 (6·2 to 25·2; −6·1 to 56·3)	Moderate	3 (1)	3	−3·0 (NA; −90·4 to 31·4)	Low
Strengthening infrastructure only	3 (3)	3	13·0 (NA; −7·0 to 15·8)	Moderate	2 (2)	2	152·1 (NA; 4·2 to 300·0)	Moderate
Supervision plus other management techniques plus any training	5 (2)	4	11·4 (0·7, 11·4; −16·2 to 26·7)	Low	2 (2)	2	30·1 (NA; 28·3 to 31·9)	Low
Patient support plus any training	6 (3)	6	11·2 (2·6 to 15·3; −6·4 to 31·5)	Low	1 (0)	1	73·3	Very low
Any training only	78 (33)	31	10·3 (6·1 to 20·7; −19·9 to 60·8)	Low	16 (8)	10	17·5 (0·1 to 23·7; −25·0 to 81·4)	Low
Strengthening infrastructure plus supervision plus any training	4 (1)	4	8·9 (−0·8 to 39·8; −4·8 to 64·9)	Low	4 (4)	3	64·3 (31·9 to 88·7; 2·6 to 110·1)	High
Supervision plus other management techniques	4 (0)	3	7·7 (−1·3 to 11·7; −7·9 to 13·3)	Very low	2 (0)	2	94·3 (NA; −9·2 to 197·9)	Very low
Group problem solving plus information and communication technology for HCPs	3 (3)	3	6·7 (NA; −3·5 to 32·6)	High	0 (0)	0	NA	NA
Supervision plus printed information or job aid for HCPs	3 (3)	7	2·3 (NA; 2·1 to 24·4)	Moderate	3 (1)	3	−3·7 (NA; −7·1 to 16·7)	Low
Printed information or job aid for HCPs only	8 (5)	7	1·4 (−4·8 to 6·2; −13·7 to 11·6)	Moderate	3 (1)	2	−3·4 (NA; −72·0 to 6·5)	Moderate
Strengthening infrastructure plus supervision plus any training plus information and communication technology for HCPs	3 (2)	3	1·3 (NA; −1·7 to 20·1)	Moderate	0 (0)	0	NA	NA
Health system financing and other incentives only	2 (0)	2	1·2 (NA; −2·6 to 5·0)	Very low	3 (2)	2	20·4 (NA; −23·9 to 72·4)	Moderate
Information and communication technology for HCPs only	4 (4)	3	1·0 (−2·8 to 9·9; −2·9 to 15·1)	Moderate	1 (1)	1	−38·9	Low
Studies predominantly of lay or community health workers*								
Any training only	4 (0)	3	2·4* (−1·1 to 7·4; −1·2 to 9·1)	Low	0	0	NA	NA

Strategies tested by at least three comparisons with percentage outcomes or at least three comparisons with continuous outcomes. GRADE=Grading of Recommendations Assessment, Development, and Evaluation system. MES=median effect size. NA=not applicable. HCP=health-care provider.

* Effect sizes expressed as an absolute percentage-point change. See Methods section for details on adjustment. Effect sizes from studies of predominantly lay or community health workers are not adjusted.

† Unless no studies with percentage outcomes were found, in which case results of continuous outcomes were used.

The effectiveness of the 24 strategies tested by at least three comparisons for percentage or continuous outcomes is shown in table 3. First, effect sizes varied widely in most strategy groups (with greater within-strategy heterogeneity than between-strategy heterogeneity). Thus, one cannot generally conclude that one strategy is definitively more effective than another or predict the effectiveness for any strategy.

Second, two strategies used alone had median MES values close to zero: printed information or job aids for health-care providers, and ICT for health-care providers (low to moderate QOE for percentage and continuous outcomes; table 3). These findings were supported by the sensitivity analysis that only included studies with a low or moderate risk of bias (low to high QOE; appendix 1, pp 131–38). As few studies tested ICT in isolation (and the study with continuous outcomes found unexpectedly large negative effects), we explored a broadened strategy definition of “ICT with or without other strategy components” to increase contextual and implementation diversity (appendix 1, p 62). We found 28 studies with percentage outcomes (the four original “ICT only” studies plus 24 additional ICT studies; median MES 8·4 percentage points [IQR 4·7 to 16·0, range −3·5 to 59·4]) and four studies with continuous outcomes (median MES −0·2 percentage points [–19·6 to 18·2, −38·9 to 36·4]; appendix 1, p 143).

Third, the effects of the frequently used strategies of training or supervision as sole strategies tended to be moderate. Effects for training only were 10·3 percentage points for percentage outcomes and 17·5 percentage points for continuous outcomes (low QOE; table 3), with almost identical results from the sensitivity analysis of low or moderate risk-of-bias studies (10·3 percentage points for percentage outcomes [IQR 7·3 to 20·7, range −7·1 to 50·6] and 17·4 percentage points for continuous outcomes [–7·7 to 23·7, −25·0 to 81·4], with moderate QOE). For supervision only, assessed with percentage outcomes, the effects were 14·8 percentage points (moderate QOE; table 3) from the main analysis and 15·9 percentage points (IQR 5·1 to 25·2, range 0·03 to 40·4; high QOE) from the sensitivity analysis. However, effects from continuous outcomes were inconsistent and highly variable (−3·0 percentage points from three studies; IQR not applicable [NA], range −90·4 to 31·4; low QOE). The combination of training and supervision had somewhat larger effects than either strategy alone when assessed with percentage outcomes: 18·0 percentage points (very low QOE) from the main analysis and 18·8 percentage points (IQR 11·3 to 24·7, range 5·8 to 30·8; moderate QOE) from the sensitivity analysis. Effects from continuous outcomes were lower: 11·1 percentage points (low QOE) from the main analysis and −2·2 percentage points (IQR NA, range −16·3 to 7·3; moderate QOE) from the sensitivity analysis.

Fourth, group problem solving alone typically had large effects when assessed with percentage outcomes: 28·0 percentage points (low QOE) from the main analysis and 37·5 percentage points (IQR NA, range 5·5 to 61·2; moderate QOE) from the sensitivity analysis. However, effects from continuous outcomes were inconsistent and highly variable (−8·1 percentage points; IQR −24·3 to 44·2, range −28·2 to 84·1; low QOE; table 3). To explore a broadened strategy definition (group problem solving with or without other strategy components) to increase contextual and implementation diversity, we found 23 studies with percentage outcomes (median MES 12·1 percentage points, IQR 6·2 to 37·5, range −3·5 to 72·5) and eight studies with continuous outcomes (3·5 percentage points, −2·2 to 84·1, −28·2 to 375·2; appendix 1, p 142).

Fifth, we identified three strategies with very large effect sizes that were tested by few studies that mostly had a high risk of bias: the combination of strengthened infrastructure, financing, supervision, management techniques, and training; group problem solving plus training; and the combination of strengthened infrastructure, supervision, management techniques, and training (table 3). The combination of strengthened infrastructure, financing, supervision, management techniques, and training had a median MES of 57·7 percentage points from three studies with percentage outcomes (moderate QOE; table 3). However, a broadened definition of nine studies, which allowed other components to be added to the core strategy, revealed substantially lower effects of 32·8 percentage points (IQR 6·6 to 58·7, range 4·4 to 60·6; appendix 1, p 141). Median MES for group problem solving plus training was 56·0 percentage points from four studies with percentage outcomes (moderate QOE; table 3). A broadened definition of 14 studies yielded substantially lower effects of 16·1 percentage points (IQR 10·2 to 34·9, range 1·5 to 77·8; appendix 1, p 141). The combination of strengthened infrastructure, supervision, management techniques, and training had effects of 33·1 percentage points from two studies with percentage outcomes (low QOE) and 183·2 percentage points from four studies with continuous outcomes (moderate QOE; table 3). A broadened definition of 17 studies with percentage outcomes led to similar effects of 29·4 percentage points (IQR 10·7 to 36·9, range 4·4 to 60·6), and a broadened definition of nine studies with continuous outcomes found reduced (but still large) effects of 76·1 percentage points (69·4 to 297·1, −7·4 to 615·5; appendix 1, p 141).

Finally, we examined three strategies that were tested by fewer than three studies (appendix 1, pp 111–23) but that have received much attention in recent years: financial incentives for health-care providers, other financing and incentives, and strategies targeting regulation and governance.^2^ Financial incentives for health-care providers as a sole strategy were tested by two studies with percentage outcomes (median MES 26·0 percentage points) and by one study with continuous outcomes (MES 66·7 percentage points; appendix 1, p 114). Effects from a broadened definition of ten studies with percentage outcomes were substantially lower (7·2 percentage points, IQR 6·6 to 40·8, range 5·0 to 60·6), as were effects from a broadened definition of eight studies with continuous outcomes (18·9 percentage points, 3·5 to 66·7, −29·3 to 375·2; appendix 1, p 143). Health system financing and other incentives as a sole strategy were tested by two studies with percentage outcomes (median MES 1·2 percentage points) and by three studies with continuous outcomes (median MES 20·4 percentage points; table 3). Effects from a broadened definition of 38 studies with percentage outcomes were larger (14·2 percentage points, IQR 7·2 to 28·8, range −2·9 to 60·6), and effects from a broadened definition of 23 studies with continuous outcomes were lower (5·9 percentage points, −0·4 to 20·4, −25·4 to 375·2; appendix 1, p 143). We found no studies of regulation and governance as a sole strategy. Effects from a broadened definition of ten studies with percentage outcomes were large (27·6 percentage points, IQR 3·9 to 36·9, range −0·8 to 60·6), as were effects from a broadened definition of five studies with continuous outcomes (20·3 percentage points, 3·5 to 70·0, 3·5 to 121·3; appendix 1, p 143).

To characterise the contexts in which a strategy might be more effective, we stratified results of percentage outcomes according to the level of resources of the setting where the study was done (appendix 1, p 62). Results for strategies tested with at least three comparisons in each resource level are presented in appendix 1 (pp 145, 146). For three strategies (group problem solving only, patient support plus training, and supervision plus training), the effects were larger by at least 10 percentage points in moderate-resource settings compared with low-resource settings. For the remaining three strategies, effect differences between the two resource strata were less than 10 percentage points. A similar analysis found larger effect sizes in studies of inpatient-only settings compared with outpatient-only settings for training only (inpatient median MES 20·7 percentage points [11 study comparisons], outpatient median MES 9·0 percentage points [45 study comparisons]) and for supervision plus training (inpatient median MES 24·7 percentage points [three study comparisons], outpatient median MES 18·8 percentage points [21 study comparisons]).

Results of the fourth sensitivity analysis, in which we analysed effectiveness with unadjusted and unweighted effect sizes, and the meta-analysis are presented in appendix 1 (pp 147–58).

Among the 18 strategies that predominantly targeted lay health workers, only one was tested by at least three study comparisons: any training as a sole strategy (median MES 2·4 percentage points; low QOE; table 3). Strategies that included community support plus training, with or without other components, tended to have larger effect sizes. Five strategies were assessed with percentage outcomes (range 8·2 to 56·2 percentage points; very low to moderate QOE; appendix 1, pp 124–26), and four were assessed with continuous outcomes (range 23·3 to 125·0 percentage points; very low to moderate QOE; appendix 1, pp 124–26).

## Discussion

Our goal was to identify effective strategies for improving health-care provider performance in LMICs. Improved health-care provider performance should lead to stronger health systems and better health outcomes for individuals and populations. To the best of our knowledge, the HCPPR is the most comprehensive review on this topic.

The first main conclusion was that a large number of studies (n=337) exist with relatively robust study designs on the effectiveness of strategies to improve health-care provider practices in LMICs. These studies evaluated a diversity of strategies (n=118) to improve performance for numerous health conditions, which were tested in a wide variety of settings. The task of synthesising the results was greatly complicated by the methodological and contextual heterogeneity of the studies, heterogeneity of interventions within strategy groups, and shortcomings of the evidence base. In particular, the majority of strategies were tested by a single study, which limits generalisability; studies generally had short follow-up times, which reduces their relevance to programmes that require strategies with sustained effect; and most studies had a high risk of bias, which contributed to the low evidence quality for most strategies.

Second, we identified several cross-cutting findings about strategy effectiveness. Two strategy component categories had significant marginal effects: group problem solving and training. We found that effectiveness was unrelated to the number of components in the strategy. We observed substantial variation in effect sizes within most strategies, including inconsistencies between MES values from percentage and continuous outcomes (and sometimes large variability for continuous outcomes), which raises questions about the utility of continuous outcomes in this systematic review. These inconsistencies are likely to be due to differences in outcome definitions, measurement methods, and how effect sizes are calculated (especially the relative change for continuous outcomes [equation 2], which can lead to very large effect sizes when baseline values are small). Potential causes of within-strategy heterogeneity of effect sizes include contextual variability, methodological differences (eg, outcomes for practices with varying degrees of difficulty, and differences in measurement methods), heterogeneity of strategies within our categories (eg, community supports varied substantially; appendix 1, pp 39, 40), implementation heterogeneity (eg, varying strength of implementation, and modification of the strategy over time), study biases (eg, unreliable outcomes, data quality changing over time, and error from non-representative samples), and imprecision from small sample sizes. The variability of effect sizes demonstrates the difficulty in predicting the effectiveness of any strategy and suggests that it is important to monitor actual effects in a given practice setting.

Third, our results support several conclusions about the effectiveness of strategies to improve practices of professional health-care providers. Among strategies assessed by percentage outcomes, most (87 [86·1%] of 101) had median MES values less than 30 percentage points, which means that even after implementing improvement strategies, important performance gaps will probably remain. Assuming typical baseline performance of 40% and an optimistic strategy effect of 30 percentage points, post-intervention performance would be 70% (ie, 40 plus 30 percentage points), or about a third of patients not receiving recommended care.

With regard to specific strategies, we found that printed information or job aids for health-care providers only and ICT for health-care providers (alone or combined with other components) typically had small to modest effects. The often-used strategies of supervision only and training only tended to have moderate effects. Compared with training in isolation, effect sizes were generally larger when training was combined with other strategies, such as supervision and group problem solving. Group problem solving only can have large effects, but a more comprehensive assessment (based on a broadened definition) suggests that it has moderate effectiveness. Two specific multifaceted strategies targeting infrastructure, supervision, other management techniques, and training (with and without financing), and the combination of group problem solving and training often had large effects. Financial incentives for health-care providers had modest to moderate effects, as did health system financing and other incentives. Studies of regulation and governance strategies tended to have large effects, but they were not studied in isolation; thus it is difficult to know how much these improvements were due to the impact of other strategy components (usually including training or supervision or both, which typically have moderate effects of their own). Our analyses also suggest that certain strategies (eg, group problem solving, and training with either patient support or supervision) might be more effective in areas with higher levels of resources than in low-resource settings (appendix 1, pp 145, 146), and other strategies (eg, training only or supervision plus training) might be more effective in inpatient settings than in outpatient settings. However, the types of practices and intervention approaches in inpatient and outpatient settings are quite different.

Several of these conclusions were informed by the sensitivity analysis that broadened a strategy's definition. When effects from a broadened definition with a larger number of studies are lower than those from a narrow definition, they might better reflect typical improvements in a programmatic setting.

Finally, we identified few studies examining strategies to improve lay or community health worker practices, and many of these studies had a high risk of bias. Training lay health workers seemed to have a small effect size, and combining training with community support might be more effective.

For strategies examined by the HCPPR and other systematic reviews, results are generally similar. For example, median effect sizes from the review by Holloway and colleagues,^6^ which only included studies from LMICs, were generally consistent with our results for training only, supervision only, printed information for health-care providers only, health-care provider training and community education, and health-care provider training plus supervision plus patient or community education. Our results matched those of reviews that included studies primarily from high-income countries for training only,^29^ printed information for health-care providers only,^30^ collaborative improvement^14^ (which was in our group problem solving category, if our more comprehensive assessment with broadened definitions is considered), audit with feedback^31^ (which was in our supervision category; panel 1), and the absence of association between number of strategy components and effect sizes.^15^, ^28^ We had difficulty comparing our results on training of lay health workers with those of the review by Sorsdahl and colleagues^32^ because that review only included two studies that had divergent results.

The HCPPR has several important limitations. First, many included studies had notable shortcomings, such as inadequate detail about strategy and context (including how and why strategies were chosen), heterogeneity of strategy implementation approaches and outcomes, difficulty in assessing study precision and strength of implementation, and high risk of bias. Second, our analytical approach, which we intentionally designed to identify broad patterns across all studies, meant that results do not reflect some important nuances. For example, all ICT strategies were considered equivalent. Future analyses will benefit from more specific classification (eg, separating results of different ICT strategies). Third, our analysis of the effect of study context was simplistic, as resource levels based on rurality and national economic category might not represent resource levels at the actual study sites. Fourth, meta-analyses have some well recognised limitations.^33^, ^34^ Finally, as study settings often benefit from extra resources and the attention of investigators, it is difficult to know the degree to which study results can be generalised to non-study settings or replicated when brought to scale.^35^

Practical, evidence-based guidance about improving health-care provider practices in LMICs is presented in panel 2. The statements are worded cautiously because, in addition to the limitations mentioned previously, decisions about which strategies a programme should use in a given setting depend on many factors, such as effectiveness, cost, feasibility, and political and cultural acceptability. This report only presents information about strategy effectiveness. Recommendations for future research are presented in panel 3. These recommendations emphasise the importance of more standardised methods, stronger study designs, replication research, and a better understanding of the influence of context on strategy effectiveness.

Panel 2 **Guidance on improving health-care provider practices in low-income and middle-income countries**

**General guidance on improving health-care provider practices**

• The effects of any strategy should be monitored so that managers can know how well it is working. Monitoring data could be used to adapt strategies to local conditions and to facilitate learning as implementation proceeds, with the aim of increasing effectiveness.
• A general approach to improving health-care provider practices is for programmes to implement an initial strategy (based on research evidence and knowledge of the local context), monitor health-care provider practices, address gaps (which are to be expected) by modifying or abandoning the strategy or layering on a new one, and continue to monitor and modify as needed.*
• Decision makers should not assume that increasing the number of strategy components will increase a strategy's effectiveness.
• Any strategy might benefit from including training or group problem solving as a component, although training by itself is only moderately effective.

**Guidance for professional health-care providers (ie, in settings that do not only include lay health workers)**

• Providing printed information or job aids to health-care providers as a sole strategy is unlikely to substantially change performance.
• Information and communication technology might lead to moderately large improvements or no improvement, but it typically has small-to-modest effects.
• Training only or supervision only might produce large improvements or no improvement, but both strategies generally tend to have moderate effects. It might be more effective to combine training with other strategies, such as supervision or group problem solving.
• Group problem solving only might bring about large or small improvements, but moderate effects are more typical.
• Multifaceted strategies targeting infrastructure, supervision, other management techniques, and training (with and without financing), and the strategy of group problem solving plus training might result in very large or only modest improvements, but such strategies tend to have large effects.
• Financial incentives for health-care providers, and health system financing strategies and other incentives might lead to large or small improvements, but these incentives typically have modest to moderate effects.
• The effects of regulation and governance strategies in isolation are unknown. When combined with other components, they tended to have large effects; however, it is difficult to know how much these improvements were due to the effect of other strategy components.
• Programmes might benefit from considering the influence of context on strategy effectiveness. Certain strategies (eg, group problem solving, and training with either patient support or supervision) might be more effective in areas with higher levels of resources.† Other strategies (eg, training only or supervision plus training) might be more effective in inpatient settings.

**Guidance for settings with only lay health workers**

• Training health-care providers as a sole strategy might produce modest improvements, but effects are usually small.
• Strategies that include community support plus training for health-care providers might lead to large improvements, although the evidence is limited.

Panel 3 **Evidence-based recommendations for research on improving health-care provider practices in low-income and middle-income countries**

Future research should:

• Use more standardised methods, especially for outcomes, strategy description, explanation of how the strategy was implemented (including dose and fidelity), and characterisation of study context
• Prioritise replication studies, especially for strategies with weak supporting evidence and (based on what is known) large effect sizes (eg, training plus group problem-solving approaches such as collaborative improvement)
• Conduct rigorous studies of information and communication technology
• Use more rigorous study designs, such as interrupted time series with a randomised comparison group
• Have longer follow-up periods that match the timeframe that programmes require for improvements to be sustained (eg, at least 12 months)
• Include an assessment of strategy cost and cost-effectiveness
• Identify the attributes of commonly used strategies (especially training and supervision) that are associated with effectiveness, with the goal of designing more effective strategies
• Focus on developing a better understanding (ideally in a quantitative fashion) of how context influences strategy effectiveness
• Include more rigorous studies on strategies to improve the performance of lay or community health workers
• Consider ways to better use routinely collected data to evaluate strategies, as the use of existing data could reduce the time and cost of studies
• If resources allow it, consider replacing true control groups with a comparison group that receives a simple strategy that is likely to improve health-care provider performance, such as training only (with effects similar to the median of all strategies; table 1), to increase the likelihood that all patients and health-care providers benefit from participating in studies

In conclusion, a large evidence base exists to demonstrate the effectiveness of strategies to improve health-care provider practices in LMICs. We found that strategy effectiveness varied substantially, that some strategies seem more effective than others, and that some strategies might be better suited to certain settings. Future analyses will examine strategy effectiveness for other outcomes (eg, patient health outcomes and use of health services), head-to-head comparisons, the effect of time on strategy effect, cost, and strategy attributes associated with effectiveness. We encourage the use of the HCPPR's publicly available database for focused analyses that might be relevant to specific programmes. Results from the HCPPR should inform decision making about improving health-care provider performance in LMICs.

## Supplementary Materials

Supplementary appendix1

Supplementary appendix2

## Footnotes

* These two categories were combined into a single “any training” category after determining that there was no meaningful difference between them in terms of effectiveness.

## Footnotes

* We acknowledge that the step on addressing gaps is partly a hypothesis that should be tested, but it is also partly justified by the relatively large effect sizes found for group problem-solving strategies that involve cycles of monitoring performance and making adjustments for further improvement.

† Hospitals in low-income countries and areas in middle-income countries that are not entirely rural.
