# Supplementary material for: Effectiveness of strategies to improve health-care provider practices in low-income and middle-income countries: a systematic review
Source: Lancet Glob Health. 2018 Oct 8;6(11):e1163–75. doi: 10.1016/S2214-109X(18)30398-X (PMC6185992; doi:10.1016/S2214-109X(18)30398-X)
Supplement: Supplementary appendix1 [file mmc1.pdf]

THE LANCET
Global Health

Supplementary appendix

This appendix formed part of the original submission and has been peer reviewed. We post it as supplied by the authors.

Supplement to: Rowe AK, Rowe SY, Peters DH, et al. Effectiveness of strategies to improve health-care provider practices in low-income and middle-income countries: a systematic review. *Lancet Glob Health* 2018; published online Oct 8. http://dx.doi.org/10.1016/S2214-109X(18)30398-X.

Appendix 1 for:

Rowe AK, Rowe SY, Peters DH, Holloway KA, Chalker J, Ross-Degnan D. A systematic review of the effectiveness of strategies to improve health care provider practices in low- and middle-income countries. *Lancet Global Health*.

Section 1. PRISMA checklist

Section 2. Review protocol

Section 3. Details of the literature search (method and results) and data abstraction methods

Section 4. Methodological details

Section 5. Additional results

Section 1 - PRISMA Checklist

Section/topic	#	Checklist item	Reported on page # of Lancet GH proof
**TITLE**			
Title	1	Identify the report as a systematic review, meta-analysis, or both.	1
**ABSTRACT**			
Structured summary	2	Provide a structured summary including, as applicable: background; objectives; data sources; study eligibility criteria, participants, and interventions; study appraisal and synthesis methods; results; limitations; conclusions and implications of key findings; systematic review registration number.	1
**INTRODUCTION**			
Rationale	3	Describe the rationale for the review in the context of what is already known.	2
Objectives	4	Provide an explicit statement of questions being addressed with reference to participants, interventions, comparisons, outcomes, and study design (PICOS).	3–4
**METHODS**			
Protocol and registration	5	Indicate if a review protocol exists, if and where it can be accessed (e.g., Web address), and, if available, provide registration information including registration number.	1–2
Eligibility criteria	6	Specify study characteristics (e.g., PICOS, length of follow-up) and report characteristics (e.g., years considered, language, publication status) used as criteria for eligibility, giving rationale.	2–3
Information sources	7	Describe all information sources (e.g., databases with dates of coverage, contact with study authors to identify additional studies) in the search and date last searched.	Appendix 1, Sect. 3
Search	8	Present full electronic search strategy for at least one database, including any limits used, such that it could be repeated.	Appendix 1, Sect. 3
Study selection	9	State the process for selecting studies (i.e., screening, eligibility, included in systematic review, and, if applicable, included in the meta-analysis).	2–3
Data collection process	10	Describe method of data extraction from reports (e.g., piloted forms, independently, in duplicate) and any processes for obtaining and confirming data from investigators.	3
Data items	11	List and define all variables for which data were sought (e.g., PICOS, funding sources) and any assumptions and simplifications made.	3
Risk of bias in individual studies	12	Describe methods used for assessing risk of bias of individual studies (including specification of whether this was done at the study or outcome level), and how this information is to be used in any data synthesis.	3 and Appendix 1, Sect. 4
Summary measures	13	State the principal summary measures (e.g., risk ratio, difference in means).	3–4 and Appendix 1, Sect. 4
Synthesis of results	14	Describe the methods of handling data and combining results of studies, if done, including measures of consistency (e.g., I^2) for each meta-analysis.	4–5 and Appendix 1, Sect. 4

Section/topic	#	Checklist item	Reported on page # of Lancet GH proof
Risk of bias across studies	15	Specify any assessment of risk of bias that may affect the cumulative evidence (e.g., publication bias, selective reporting within studies).	3 and Appendix 1, Sect. 4
Additional analyses	16	Describe methods of additional analyses (e.g., sensitivity or subgroup analyses, meta-regression), if done, indicating which were pre-specified.	5 and Appendix 1, Sect. 4
**RESULTS**			
Study selection	17	Give numbers of studies screened, assessed for eligibility, and included in the review, with reasons for exclusions at each stage, ideally with a flow diagram.	5–6 and Fig.1
Study characteristics	18	For each study, present characteristics for which data were extracted (e.g., study size, PICOS, follow-up period) and provide the citations.	Appendix 2
Risk of bias within studies	19	Present data on risk of bias of each study and, if available, any outcome level assessment (see item 12).	Appendix 2
Results of individual studies	20	For all outcomes considered (benefits or harms), present, for each study: (a) simple summary data for each intervention group (b) effect estimates and confidence intervals, ideally with a forest plot.	13 (data on HCPPR website)
Synthesis of results	21	Present results of each meta-analysis done, including confidence intervals and measures of consistency.	Table 3 and Appendix 1, Sect. 5
Risk of bias across studies	22	Present results of any assessment of risk of bias across studies (see Item 15).	6 and Appendix 1, Sect. 5
Additional analysis	23	Give results of additional analyses, if done (e.g., sensitivity or subgroup analyses, meta-regression [see Item 16]).	Appendix 1, Sect. 5
**DISCUSSION**			
Summary of evidence	24	Summarize the main findings including the strength of evidence for each main outcome; consider their relevance to key groups (e.g., healthcare providers, users, and policy makers).	Table 3
Limitations	25	Discuss limitations at study and outcome level (e.g., risk of bias), and at review-level (e.g., incomplete retrieval of identified research, reporting bias).	12
Conclusions	26	Provide a general interpretation of the results in the context of other evidence, and implications for future research.	12–13
**FUNDING**			
Funding	27	Describe sources of funding for the systematic review and other support (e.g., supply of data); role of funders for the systematic review.	13

From: Moher D, Liberati A, Tetzlaff J, Altman DG, The PRISMA Group (2009). Preferred Reporting Items for Systematic Reviews and Meta-Analyses: The PRISMA Statement. PLoS Med 6(6): e1000097. doi:10.1371/journal.pmed1000097

Section 2. Protocol for a systematic review of the effectiveness and costs of interventions to improve health care provider performance and related health outcomes in low- and middle-income countries

I. PROJECT TITLE

A systematic review of the effectiveness and costs of interventions to improve health care provider performance and related health outcomes in low- and middle-income countries

II. ABBREVIATIONS

CI	Confidence interval
EPOC	Effective Practice and Organisation of Care
GRADE	Grading of Recommendations Assessment, Development, and Evaluation
HCP	Health care provider
HCPPR	Health Care Provider Performance Review
ITS	Interrupted time series
JHU-APL	Johns Hopkins University Applied Physics Laboratory
LMICs	Low- and middle-income countries
MES	Median effect size
PRISMA	Preferred Reporting Items for Systematic Reviews and Meta-analyses
WHO	World Health Organization

III. BACKGROUND AND JUSTIFICATION

Each year in low- and middle-income countries (LMICs), millions of children and adults die prematurely [WHO, 2014; GBD, 2015]; although many interventions exist that can prevent such deaths [Jones, 2003; Travis, 2004; Chisholm, 2012; Bhutta, 2014]. Low coverage of these interventions has been identified as a critical public health problem [Jones, 2003; Bhutta, 2014] and a major obstacle to achieving Millennium Development Goals [Travis, 2004] and the Sustainable Development Goals [Tangcharoensathien, 2015].

A key part of almost any strategy for increasing the effective coverage of health interventions involves health care providers (HCPs), including health workers in hospitals, clinics, pharmacies, drug shops, and communities. However, HCP performance in LMICs is often inadequate, as documented in studies of child health [Bryce, 2003; Rowe, 2001], sexually transmitted diseases [Bitera, 2002], obstetrics [Merali, 2014; Saleem, 2014], mental disorders [Abas, 2003], injuries [Bickler, 2002], diabetes [Whiting, 2003], malaria [Zurovac, 2004; Hill, 2014], medicine use [Holloway, 2013], and illnesses managed in hospitals [English, 2014] and by private sector health workers [Holloway, 2013; Morgan, 2016]. The global burden of unsafe medical care in LMICs is high, conservatively estimated at more than 33 million disability-adjusted life years lost annually [Hauri, 2004; Jha, 2013]. Notably, inadequate care occurs despite substantial efforts by governments, non-governmental organizations, and donors.

Improving HCP performance, or competence [WHO, 2006], is not only important to prevent errors of omission (e.g., a patient needing a medicine does not receive it), but also to avoid harmful practices (e.g., giving sedatives to children with pneumonia [Rowe, 2001]). Furthermore, some research suggests that improving performance might increase utilization of health services [Arifeen, 2004].

Numerous studies in LMICs have evaluated a wide variety of strategies to improve HCP performance. Systematic reviews that distill the evidence on effectiveness and cost can be valuable for guiding policy to reduce medical errors, focusing programmatic efforts on strategies with relatively greater effectiveness, and avoiding strategies that are relatively ineffective.

Many existing systematic reviews have focused on specific strategies, such as training [Amaral, 2008; Nguyen, 2013; Opiyo, 2010; Rowe, 2012; Sibley, 2004a; Sibley, 2004b], computer-based training [Knebel, 2000b], distance learning [Knebel, 2001], essential drug programs [Ratanawijitrasin, 2001], integration of services [Briggs, 2001], job aids [Knebel, 2000a; Grace, 2008], lay health workers [Lewin, 2010], self-assessment [Bose, 2001], supervision [Bosch-Capblanch, 2008; Bosch-Capblanch, 2011], incentives [Witter, 2012], and telemedicine [Wootton, 2001]. Some of these reviews focus exclusively on LMICs, while others include studies from LMICs and high-income countries. However, a key limitation of single-strategy reviews is that they only partly address the fundamental programmatic question: what are the most effective and affordable ways to improve HCP performance? To answer this broader question for the LMIC context, all strategies tested in LMICs must be examined and compared.

Several systematic reviews have included multiple, but not all, strategies. The largest of these reviews [Grimshaw, 2004] had few studies from LMICs. Two reviews presented only descriptive or semi-quantitative summaries [Siddiqi, 2005; Shah, 2011]. One review, which was updated twice, focused on strategies to improve medicine use in LMICs [Ross-Degnan, 1997; WHO, 2001; WHO, 2009; Holloway, 2013]. At least four overviews of systematic reviews of single strategies are currently underway [Ciapponi, 2014; Herrera, 2014; Pantoja, 2014; Wiysonge, 2014].

Existing reviews often have important limitations. First, they rarely summarize economic data on strategy cost or cost-effectiveness. Second, some reviews do not use methods that have become standard in the field of systematic reviews. Third, results of strategy-versus-strategy (i.e., head-to-head) comparisons are often not integrated with results of strategy-versus-control comparisons, which underutilizes a large portion of the evidence base. Fourth, the databases on which the reviews are based are either not publicly available or only available as a static table, which limits their usability. Additionally, existing reviews use such heterogeneous methods that it is difficult to synthesize their results. For example, measures of strategy effectiveness have included risk differences, adjusted risk differences, relative risks, and non-quantitative categories.

An updated quantitative systematic review of multiple strategies is needed that includes all strategies tested in LMICs, all facets of HCP performance, economic data, head-to-head studies, a publicly available database in a dynamic format, the use of a single analytic framework, and state-of-the-art methods for systematic reviews. The Health Care Provider Performance Review (HCPPR) is a systematic review designed to help fill this gap. The HCPPR, which will focus on studies from 2006 to 2016, will link with previous efforts that focused on studies up to 2006.

Now is a particularly important time to conduct systematic reviews, such as the HCPPR, on improving HCP performance. The large growth in donor funding in the past decade [Dieleman, 2015] provides an enormous opportunity to improve health in LMICs, and strengthening HCP performance has the potential to increase the effectiveness and efficiency of programs supported by such funding. Improving HCP performance will also be essential for meeting a target of the Sustainable Development Goals that calls for achieving universal health coverage, which requires “access to quality essential health-care services” [UN, 2014]. More

generally, research on improving HCP performance fits within the larger public health priorities of conducting research to strengthen human resources for health [Chen, 2004; Narasimhan, 2004] and health systems [Alliance for Health Policy and Systems Research, 2004; Task Force on Health Systems Research, 2004].

IV. OBJECTIVES

Conduct a systematic review of the effectiveness and costs of strategies to improve HCP performance and related health outcomes in LMICs, and produce the following.

1. A publicly available database of studies on improving HCP performance in LMICs for program managers and other decision-makers, policy analysts, donors, technical agencies, civil society groups, and researchers;
2. Analyses to estimate the effectiveness of a wide variety of strategies to improve HCP performance, and comparisons to identify more and less effective strategies;
3. In-depth analyses of strategies involving training and supervision to identify attributes associated with greater effectiveness;
4. Evidence-based guidance on how to improve HCP performance in LMICs; and
5. Contributions to a research agenda to fill critical knowledge gaps on how to improve HCP performance.

V. INVESTIGATORS

Names and affiliations

1.	Alexander K. Rowe	Malaria Branch, Division of Parasitic Diseases and Malaria, Center for Global Health, Centers for Disease Control and Prevention, Atlanta, United States; email: axr9@cdc.gov.
2.	Samantha Y. Rowe	Malaria Branch, Division of Parasitic Diseases and Malaria, Center for Global Health, Centers for Disease Control and Prevention, Atlanta, United States; email: say9@cdc.gov.
3.	David H. Peters	Department of International Health, Johns Hopkins Bloomberg School of Public Health, Baltimore, United States; email: dpeters@jhu.edu.
4.	Kathleen A. Holloway	World Health Organization, Southeast Asia Regional Office; and Indian Institute of Health Management Research, Delhi, India; email: kaholloway54@gmail.
5.	Dennis Ross-Degnan	Harvard Medical School, Boston, United States; Harvard Pilgrim Health Care Institute, Boston, United States; email: Dennis_Ross-Degnan@hms.harvard.edu.

Publication and authorship

Authorship decisions will be based on Harvard University guidelines (Annex 1). To be a co-author, the individual needs to have: 1) made a substantial intellectual contribution to the conception, design, analysis, and/or interpretation of data; 2) participated in writing a manuscript; and 3) approve the final version of the manuscript. Individuals who have only assisted with data management or data abstraction and individuals who have only provided informal input (e.g., providing comments on a protocol or manuscript) will be a contributor; all such contributors will be named in the Acknowledgment section.

Responsibilities

1. Drs. A. Rowe, S. Rowe, Peters, Holloway, and Ross-Degnan will all help plan, review, and edit the protocol, reports, and manuscripts. All co-investigators will contribute to analyzing data, interpreting results, and resolving technical and general strategic issues.
2. Dr. A. Rowe will be the primary investigator. He will ensure that key information regarding the review will be communicated among all co-investigators. He will be responsible for identifying technical and general strategic issues and ensuring that these issues are resolved through the consensus opinions of all co-investigators. He will be the point of contact with donors who fund the review and contractors who support the review. He will oversee the protocol development, literature search, data abstraction, data management, analysis, and dissemination of results. He will write initial drafts of reports or manuscripts, or delegate this responsibility, as needed.
3. Dr. S. Rowe will provide day-to-day oversight to members of the data abstraction team. She will also be the primary data manager and analyst.

VI. METHODS

General description

This project is a systematic review of published and unpublished studies that meet minimum study design criteria and that quantitatively evaluate a strategy to improve HCP performance in LMICs. The methods are based on those used by Ross-Degnan and colleagues [Ross-Degnan, 1997], WHO [WHO, 2001], Grimshaw and colleagues [Grimshaw, 2004], and the Cochrane Effective Practice and Organisation of Care (EPOC) Group [EPOC, 2015; EPOC, 2013]. The review will adhere to the Preferred Reporting Items for Systematic Reviews and Meta-analyses (PRISMA) guidelines [Moher, 2009] and guidelines for reporting economic studies [Drummond & Jefferson, 1996].

Inclusion and exclusion criteria

1. <u>Timing.</u> The literature search will focus on studies published from 2006 to 2016.
2. <u>Setting.</u> LMICs were defined as countries with a low, lower-middle, or upper-middle income economy, according to the World Bank [World Bank, 2015].

3. Type of health condition. Performance related to any health condition is eligible. Studies aiming to improve performance for activities unrelated to clinical preventive or curative care are excluded (e.g., improving research skills of HCPs [Dodani, 2012]).
4. Type of HCP. Any hospital- or health facility-based health worker, community health worker (anyone who is part of a recognized community health worker program), pharmacist, or shopkeeper who sell medicines. Private sector health workers with at least some medical or para-medical training will also be included. We will exclude studies of: a) household-based providers (e.g., a patient's family or neighbors), unless the provider is a community health worker; and b) traditional healers who were not part of a well-defined program to implement standards of care based on "Western" or allopathic/osteopathic medical principles.
5. Type of intervention. Any intervention strategy with at least 1 component that plausibly could affect HCP performance either directly (e.g., training, supervision, or HCP incentives) or indirectly, by changing the physical, economic, or policy environment in which HCPs work (e.g., providing essential medicines, changing user fees, or implementing new health regulations). We will exclude studies of strategies without any component directly or indirectly targeting HCPs (e.g., only community education by radio broadcasts).
6. Study design. Eligible study designs are shown below. As different outcomes in the same study could be based on different study designs, studies need to have at least one primary outcome based on an eligible study design. For example, a pre- versus post-intervention study with a non-randomized comparison group would be excluded if the primary outcomes were only measured at follow-up.
    a. Randomized controlled before-and-after trials
    b. Non-randomized controlled before-and-after trials
    c. Randomized controlled post-intervention only trials
    d. Interrupted time series (ITS) designs with at least 3 data points before and after the intervention, with or without comparison groups
7. Type of study outcomes. There are no restrictions on type of study outcome. Only primary outcomes are eligible. Primary outcomes are those defined by the study authors. If authors do not designate any outcomes as primary, we will define primary outcomes based on the study objectives (which sometimes means including all outcomes). See the Analysis section for a description of how the heterogeneity of study outcomes will be addressed. The following outcomes will be excluded.
    a. Outcomes with a trend that is difficult to interpret—i.e., it is unclear whether an increase or decrease in the outcome reflects an improvement. For example, the outcome "percent of time spent performing curative care" would be ineligible if it is unclear whether the strategy was designed to increase time spent on curative care, as opposed to other activities, such as immunizations or antenatal care.
    b. Outcomes that are similar to another outcome that is already being included. For example, we will exclude the complement of an already-included outcome (e.g., if an outcome is "% of patients receiving an injection", we would exclude "% of patients not receiving an injection") or subgroup-specific results of an already-included outcome (e.g., if an outcome is "% of patients treated correctly", we would exclude "% of males treated correctly"). The one exception to this rule is for well-accepted outcomes of health impact. For example, we will include both

"deaths among children under 5 years old per 1000 live births" and "neonatal deaths per 1000 live births", even though the latter is a subset of the former.

8. Effect sizes. Eligible effect sizes (defined below, in the Analysis section) are those based on an eligible, primary outcome from an eligible study design. The following effect sizes will be excluded.
    a. Effect sizes based on outcomes that are not compared between two study groups or are not compared over time in one study group (e.g., outcomes that are reported as the combined result of all study groups and thus no effect size can be calculated)
    b. Effect sizes based on <20 observations per study group and time point, for a given comparison
    c. Effect sizes based on a simulation study and not actually observed data
    d. Effect sizes based on measures of 100% at baseline and follow-up in the intervention group, as this indicates that HCP performance in the intervention group had no room for improvement and did not worsen over time. Similarly, for outcomes on HCP practices expressed as a percentage, we excluded effect sizes based on a baseline value of 95% or greater, as there was little room for improvement.
    e. Effect sizes based on outcome measures that were not taken at comparable times between study groups. For example, if the outcome for a control group was measured at –1 month, 3 months, and 9 months since the intervention began, and the outcome for an intervention group was measured at –1 month, 3 months, and 21 months since the intervention began, the effect size based on the 9-month and 21-month outcome measures would be ineligible.
    f. For ITS, we will exclude study outcomes for which the time series was highly unstable and thus could not be reliably modeled, and we will also exclude outlier outcome measures that probably did not represent the true trend in HCP performance (e.g., an unusually high baseline measure just before a strategy was implemented that was likely due to HCPs' anticipation of the strategy).
9. Outcome measures: All outcome measures of eligible outcomes will be abstracted.
10. Sample size. Studies must have at least one eligible effect size (i.e., at least one effect size for an eligible study outcome that is based on at least 20 observations per study group and time point).
11. Adequacy of statistical analysis. No exclusion criteria.
12. Language. No restriction on language of the studies.

Literature search strategy

The literature search strategy will include the following nine components. To assess the sensitivity of the literature search, we will calculate the percentage of a pre-selected group of 82 studies from our library (already known to be eligible) that are identified by the literature search.

1. We will search 16 electronic databases of published studies by: a) applying a filter of search terms to obtain a subset of citations that would likely include eligible studies, b) ranking the resulting citations with a computer algorithm developed by the Johns Hopkins University Applied Physics Laboratory (JHU-APL), and c) screening citations at

the top of the list (we will keep going down the list as long as the search yields a reasonable number of eligible studies). See Table A in Annex 2 for the list of databases, and see Text box 1 in Annex 2 for details of the search terms for the PubMed database. The JHU-APL computer algorithm uses linear kernel support vector machines, which were trained using about 30,000 citations categorized as “include” or “exclude” (from previous systematic review work on health worker performance) to create a hyperplane to separate the new citations into “include” or “exclude” categories. For each citation, the distance between the citation’s vector and the hyperplane will be used to create a rank.

2. We will search 31 electronic databases of published studies (that are different from the 16 databases described above in component 1) by: a) applying a filter of search terms to obtain a subset of citations that would likely include eligible studies, and b) screening all the resulting citations. This group of databases will not be ranked by the JHU-APL algorithm because the JHU-APL staff will not be able to process the databases, given the available resources. See Table B in Annex 2 for the list of databases.
3. We will assess 34 electronic databases of published studies that were recommended by EPOC (EPOC, 2013) (that were different from the 47 databases described above in components 1 and 2), but we decided not to search them because either: a) the database included studies primarily from a high-income country, b) studies in the database were in another database already searched, c) the database required a paid subscription and included few studies from LMICs, or d) the database (including the database’s search engine) was in a language that was difficult for the HCPPR Team to translate and work in (e.g., Turkish or Vietnamese). See Table C in Annex 2 for the list of databases and the reason for exclusion.
4. We will search 15 electronic databases of published studies (largely overlapping the databases in component 1, above) by: a) downloading all studies in the database, b) ranking the citations with the JHU-APL computer algorithm, and c) screening the top citations (we will keep going down the list as long as the search yields a reasonable number of eligible studies). See Table D in Annex 2 for the list of databases.
5. We will search websites of nine scientific conferences and technical meetings to identify unpublished studies. See Table E in Annex 2 for the list of conferences and meetings.
6. We will search websites of 43 organizations that work on HCP performance issues to identify unpublished studies. See Table F in Annex 2 for the list of organizations.
7. We assessed seven other websites, but we decided not to search them either because: the website had not been updated since 2006, the website was no longer active, or we lacked the resources to search them. See Table G in Annex 2 for the list of websites.
8. We will screen the bibliographies of review articles on HCP performance identified in the search that were published in 2006 or later.
9. We have contacted 45 experts for study reports and bibliography lists, and will include any recommended study not already identified. See Table H in Annex 2 for the list of experts.

Screening search results and data abstraction

Results of the literature search will be screened by a team of investigators and trained research assistants. Before beginning, concordance testing will be conducted against a “gold standard” list of reports until at least 80% can be identified by each team member. Titles and

abstracts from the literature search will be reviewed to identify potentially eligible reports. If the title or abstract is insufficient for assessing eligibility, a full text version will be obtained and screened.

Data abstraction will also be performed by a team of investigators and trained research assistants. Before beginning, concordance testing of all team members will be conducted until the percent agreement between individual abstractors and a gold standard set of abstracted data (based on consensus by several investigators) is >80%. We will also assess concordance for "paired abstraction" in which a pair of reviewers independently abstracts data and resolves discrepancies. Data from each study will be abstracted independently by two study team members and entered into a Microsoft Access database (Microsoft Inc. Redmond, Washington). Data abstracted by the two team members will be compared and discrepancies will be reconciled (with consultation with a third team member, if necessary).

Data elements that will be abstracted include: study setting (where, when, HCP types, other contextual factors), study design, health conditions addressed, strategy description (see Annex 3), outcome description, outcome measurements, the timing of outcome measurements in relation to the implementation of the strategy, effect sizes, sample sizes, sampling details, and data elements needed to assess risk of bias (see Annex 4).

For crossover trials, although the classical analysis includes data from before and after the crossover of strategies, we only include data on each study group before the crossover. The justification is that post-crossover data are likely to be biased by the contamination of strategies before the crossover.

In rare situations, a study will be split into different records (i.e., sub-studies), such that the effect sizes in each record perfectly correspond with the strategy components coded in the record. Situations in which a study will be split into sub-studies are:

1. When distinct intervention components in a single study group are implemented far apart in time, not intended to be implemented as a package, with observations between components' implementation (e.g., one sub-study [record 1] examines the effect of training only, and another sub-study [record 2] examines the combined effect of training and supervision)
2. When two intervention groups have different timing of strategies and have observations between components' implementation, we will abstract data as if there are two studies: one sub-study examining the impact of a strategy compared to a non-intervention control (i.e., before a strategy is introduced to an intervention group, it serves as a non-intervention control for the other intervention group), and a second sub-study examining the marginal impact of one strategy over another (a head-to-head comparison). For example, one record examines the effect of training only, and another record examines the marginal effect of adding supervision to training.
3. When a strategy involved health facility- and community-level components that are implemented and evaluated separately over time (e.g., facility components implemented and evaluated in study years 1–2 and community components implemented in study years 3–5 and evaluated during all study years [Arifeen, 2009]), with separate outcomes measured at the facility and community levels, we will abstract data as if there are two studies: one sub-study examining the effect of facility-level components on facility-level outcomes, and a second sub-study examining the effect of both facility- and community-level components on community-level outcomes.

Data collection from study authors

We will request information from study authors to: 1) resolve inconsistencies in a study report (e.g., a result from a table does not match a result in the text of a study report), or 2) obtain information for the HCPPR database that could not be found in a study report. We will make up to four attempts to contact study authors, via email or telephone, with at least one week between attempts.

Analysis

<u>Assessment of risk of bias.</u> Our method is based on guidance from the Cochrane EPOC Group [EPOC, 2015]. Risk of bias at the study level will be categorized as low, moderate, high, or very high. Randomized studies, ITS, and non-randomized studies are initially categorized as low, moderate, and high risk of bias, respectively. We will then assess the following risk of bias domains: number of clusters per study arm, complete dataset, balance in baseline outcome measurements, balance in baseline characteristics, reliable outcome, adequacy of concealment of allocation (where relevant), intervention unlikely to affect data collection, intervention plausibly independent of other changes, and number of data points before and after the intervention. Some domains only apply to certain study designs. A study's risk of bias category will be dropped by one level for every applicable domain that was "not done" and for every two applicable domains that are "unclear" (see Annex 4 for details). Separate analyses will be conducted for all studies and only studies with a low or moderate risk of bias.

<u>Assessment of publication bias.</u> To identify publication bias, we will examine results for studies of all strategies in a particular outcome group with at least 10 comparisons per strategy. We will inspect funnel plots and use Egger's test of asymmetry (significance of $p < 0.1$) [Egger, 1997].

<u>Estimating effect sizes for strategies at the study outcome level.</u> Effect sizes are defined as absolute percentage-point differences and calculated such that positive values mean improvement. Thus, for study outcomes designed to decrease (e.g., percent of patients receiving unnecessary treatments), we will multiply effect sizes by –1. Details on effect size calculations are presented in Annex 4, but the most common formulae are presented below.

1. In non-ITS studies with pre- and post-intervention outcome measures, for outcomes that are dichotomous or expressed as a percentage, the effect size = $(\text{follow-up} - \text{baseline})_{\text{intervention}} - (\text{follow-up} - \text{baseline})_{\text{control}}$
2. In non-ITS studies with pre- and post-intervention outcome measures, for outcomes that are continuous but not obviously bounded (e.g., a mortality rate), the effect size = 100% x $\{[(\text{follow-up} - \text{baseline})/\text{baseline}]_{\text{intervention}} - [(\text{follow-up} - \text{baseline})/\text{baseline}]_{\text{control}}\}$
3. For ITS studies, segmented linear regression modeling [Wagner, 2002] will be performed to estimate a summary effect size that incorporates both the level and trend effects. The summary effect size is the outcome level at the mid-point of the follow-up period as predicted by the regression model minus a predicted counterfactual value that equals the outcome level based on the pre-intervention trend extended to the mid-point of the follow-up period (see Annex 4 for details). This summary effect size will be used because it allows the results of ITS studies to be combined with those of non-ITS studies.

We will explore the possibility of adjusting effect sizes for contextual and methodological factors that might differ among effect sizes and strategy groups. These factors (e.g., baseline performance level) are potential effect modifiers. For example, several studies have found that effect sizes tend to be larger when baseline performance levels are lower [Franco, 2011; Jamtvedt, 2006; Nguyen, 2013; Rowe, 2013; Shojania, 2006]. Random-effects linear regression modeling will be used to identify effect modifiers. If statistically significant effect modifiers are identified, the model results will be used to adjust effect sizes (see Annex 5). Use of the adjusted effect sizes should reduce bias when comparing strategies by creating a partly standardized study context (e.g., a context in which all studies have the same baseline performance level).

Analysis overview. To achieve the HCPPR's objective of developing evidence-based guidance on improving HCP performance by comparing results for many different strategies (from potential heterogeneous studies), three analytic steps are required.

1. Define a series of mutually exclusive strategy groups and categorize each strategy into one strategy group.
2. Determine which studies and which results can be meaningfully compared, and to which settings the results can be generalized.
3. Within the groups of results that can be compared: estimate the effectiveness of the strategy groups, assess the quality of the evidence on effectiveness, and make comparisons among strategies in a way that accounts for or reduces bias from outliers, small numbers of studies per strategy, unequal sample sizes, methodological and contextual differences among the studies, and comparison type (intervention versus control, and head-to-head).

Step 1: Defining strategy groups

To define a series of mutually exclusive strategy groups and categorize each strategy into one strategy group, we will first code the presence of about 200 detailed strategy components for each study arm exposed to an improvement strategy (see Annex 3). These detailed strategy components have been grouped into 13 component categories. We define a unique strategy as any unique combination of the 13 component categories. The 13 component categories were developed based on conceptual considerations (i.e., which strategy components seemed similar in terms of method, target population, mechanism of action, and in the case of training, the intensity of the strategy) and not based on effect sizes. In future analyses, the 13 component categories can be disaggregated to examine strategies defined at a more granular level.

We recognize that strategies are often different, even when they have the same label—even when strategies are defined by the ~200 detailed strategy components. The reasons are that strategies are closely linked to the context in which they are implemented and the stakeholders involved, and that strategies frequently change during implementation, as is common with "quality improvement" interventions. Our approach is to try to compare "like with like". This approach involves standardizing the strategy groups as much as possible, accounting for how strategies change (either by assessing them when they are "fixed" interventions or by defining strategies that include change or continuous learning as part of the strategy), and by considering differing contexts.

Placebo strategy components will be coded as placebos in the review's database and will be ignored in the analysis. For example, control groups that are exposed to the placebo strategy "training on herbal medicine" will be analyzed together with control groups that received no new

intervention. Note that we describe control groups as receiving "no new intervention" because all HCPs are constantly exposed to pre-existing or "business as usual" interventions (e.g., routine supervision and provision of medical supplies).

Step 2: Determining which results can be compared

To determine which results can be compared, four attributes will be used: study type, outcome type, outcome scale, and HCP cadre. We first distinguish between non-inferiority studies with gold standard HCPs in the control group (e.g., a study to determine if trained nurses in the intervention group could perform vasectomies as well as physicians in the control group) and all other studies (e.g., a study of in-service training, with a control group of HCPs without the training). These study types will be analyzed separately because a successful result of the first study type is an effect size close to zero. In contrast, a successful result of the second study type is typically non-zero. For each study type, we will categorize effect sizes into 24 subgroups (Table 1), according to six outcome categories (e.g., processes of care, health outcomes, etc.), two outcome scales (percentages and other continuous outcomes), and two HCP cadres (facility-based HCPs and lay health workers). Comparisons are only made within subgroups (e.g., cell *a* results are not compared with cell *b* results). In future analyses, the outcome categories and HCP cadres can be sub-divided to outcomes and HCP cadres defined at a more granular level.

Table 1. Approach for creating groups of results within which comparisons can be made

Outcome type	Health care providers (HCPs) in the study	
	Predominantly health facility-based HCPs[a]	Predominantly lay health workers[b]
Outcomes expressed as percentage		
Elements that facilitate HCP performance (e.g., supplies)	*a*	*b*
Processes of care (e.g., correct treatment)	*c*	*d*
Health outcomes	*e*	*f*
Utilization of health services or care-seeking	*g*	*h*
Other patients behaviors (e.g., adherence to treatment regimen)	*i*	*j*
Cost	*k*	*l*
Continuous outcomes not expressed as percentage		
Elements that facilitate HCP performance (e.g., supplies)	*m*	*n*
Processes of care (e.g., correct treatment)	*o*	*p*
Health outcomes	*q*	*r*
Utilization of health services or care-seeking	*s*	*t*
Other patients behaviors (e.g., adherence to treatment regimen)	*u*	*v*
Cost	*w*	*x*

Footnotes.

[a] Studies of physicians, nurses, midwives, and other HCPS that typically work in a health facility. Studies in this group could include lay health workers, but other HCPs are also exposed to improvement strategies.

[b] Studies for which improving lay health worker performance is the primary focus. The context might include other HCPs (e.g., village lay health workers might refer seriously ill patients to nurses), but improving the performance of these other HCPs is not the study focus.

Step 3: Estimate strategy effectiveness, assess evidence quality, and compare strategies

To estimate strategy effectiveness from a single study comparison (i.e., a comparison of two study arms[1]), the effect size is defined as the median of all effect sizes in the comparison for outcomes in the same outcome group (i.e., in the same cell in Table 1). Median effect sizes (MES), which have been used in other systematic reviews [Ivers, 2012; Holloway, 2013], simplify the analysis (i.e., one effect size per comparison) and reduce the influence of outliers.

Several methods will be used to estimate strategy effectiveness from multiple studies and make comparisons among comparisons in ways that account for or reduce bias from outliers, small numbers of studies per strategy, unequal sample sizes, methodological and contextual differences among the studies, and comparison type (intervention versus control, and head-to-head). As no single method can satisfy all these requirements, we will use a primary and two secondary analyses—each with advantages and limitations. To assess the quality of the evidence on the effectiveness of each strategy, the Grading of Recommendations Assessment, Development, and Evaluation (GRADE) system will be used (Guyatt, 2011).

The primary analysis will only include comparisons of an intervention versus a control group. Each study comparison will be summarized with a MES, based on adjusted effect sizes if effect modifiers are identified (see Annex 5). The effectiveness of each strategy group will be described with a median MES, interquartile range, minimum, and maximum. Medians for strategy groups that are based on less than five study comparisons will not be weighted, as weighting with small samples might cause the median to be a poor measure of central tendency when outliers are present. Medians for strategy groups with five or more study comparisons will be weighted, with the weight = 1 + the natural logarithm of the number of HCPs or (if the number of HCPs in a study is not reported) the number of service provision sites (e.g., health facilities) or (if the number of service provision sites is not reported) the number of administrative areas (e.g., districts) in the study. Strategy groups tested by at least three study comparisons will be considered to have enough evidence to form generalizations—although caution is increasingly warranted as the minimum of three comparisons is approached. Strategy groups tested by only one or two study comparisons will be interpreted separately.

Secondary analyses

In the first of the two secondary analyses, comparisons will be summarized with an MES, as described above. Standard random-effects meta-analysis will be used to estimate the weighted mean MES and 95% confidence interval (CI) of each strategy group [Borenstein, 2009]. The main advantages of this method are that the influence of a study depends on its size and that comparisons among strategy groups can be made with means and CIs, which better characterizes the role of chance. We will use I^2 as a measure of consistency for each meta-analysis (I^2 "describes the percentage of the variability in effect estimates that is due to heterogeneity rather than sampling error" [Higgins, 2002; Higgins, 2003]). We will also conduct standard meta-regression on MES calculated with unadjusted effect sizes. The key advantages of these methods are: 1) study size is accounted for, 2) comparisons among strategy groups can be made with means and CIs, and 3) the adjustment for effect modifiers reduces bias of strategy-to-strategy comparisons.

---

[1] For ITS studies with one study arm, a comparison is between pre- and post-intervention results.

Sometimes (perhaps often) standard errors of outcome measures reported in study articles, which are needed to perform meta-analysis and meta-regression, are not valid because most studies have correlated data (e.g., patients "clustered" within health facilities) and the correlation is ignored in the analysis. When valid standard errors are not available, we will take sample size information from the studies, apply some conservative assumptions about data correlations (e.g., an intra-class correlation of 0.4), and calculate approximate, conservative estimates of standard errors (see Annex 5 for details).

In the second secondary analysis, we will use network meta-analysis [Jansen, 2011] to quantitatively integrate head-to-head comparisons with strategy-to-control comparisons.

<u>Sensitivity analyses.</u> Four pre-specified sensitivity analyses will be conducted. First, we will repeat the above analyses for only studies with a low or moderate risk of bias. Second, we will perform a meta-analysis for strategies that included training to identify factors associated with greater training effectiveness. A similar analysis will be conducted for supervision. Third, for strategies with large effect sizes, we will examine whether the large effect sizes could be due to limited contextual diversity. This analysis will involve broadening the strategy definition to include strategies with the same set of core components but with other components allowed. For example, for the strategy "group problem solving only", the sensitivity analysis would involve a calculation of the effectiveness of group problem solving with or without additional strategy components. We assume that adding components is unlikely to reduce effectiveness. If the median MES of a strategy group with a broadened definition is lower than that of the original (narrower) strategy group definition, then bias is likely. If bias from limited contextual diversity is likely present, then we will use the median MES from the broadened definition to represent the effectiveness of the strategy group. The fourth sensitivity analysis is designed to better characterize the contexts in which a strategy might be more or less effective. For strategies tested by at least three comparisons each, we will stratify results according to the level of resources and development where the study was conducted. The level of resources and development will be categorized as either "low" (i.e., any non-hospital setting in a low-income country and rural-only settings in middle-income countries) or "moderate" (i.e., hospitals in low-income countries and any urban and mixed urban/rural setting in a middle-income country). Our classification of a country's economy as low versus middle income is based on the World Bank's economy category for that country. For the small number of multi-country studies in both low- and middle-income countries, the categorization of level of resources assumed the study was from a middle-income country.

<u>Other analyses.</u> Other analyses will include: 1) time trends of study quality (i.e., risk of bias) and geographies where studies were conducted, and 2) descriptive analyses of baseline values.

VII. DISSEMINATION

Results will be disseminated via scientific publications; presentations at scientific conferences; and presentations, webinars, and consultations by HCPPR investigators for staff of ministries of health in LMICs, non-governmental organizations engaged in health activities in LMICs, donor agencies, academic institutions, and other public health institutions. In addition, a website will be launched that will include recorded presentations, scientific publications, and

downloadable databases from the review. The website will also have a user-interface that will allow website visitors to perform basic descriptive analyses using a series of pre-specified menu options (e.g., to display results from all studies from Africa or all studies on community-based HCPs). HCPPR staff will also provide technical assistance (to the extent that resources are available) to individuals who want to perform targeted analyses of the data.

VIII. REFERENCES

Abas M, Baingana F, Broadhead J, Iacoponi E, Vanderpyl J. Common mental disorders and primary health care: current practice in low-income countries. *Harvard Review of Psychiatry* 2003; 11: 166–73.

Alliance for Health Policy and Systems Research. Strengthening health systems: the role and promise of policy and systems research. Geneva: Alliance for Health Policy and Systems Research, 2004.

Amaral JJ, Victora CG. The effect of training in Integrated Management of Childhood Illness (IMCI) on the performance and healthcare quality of pediatric healthcare workers: a systematic review. Revista Brasileira de Saúde Materno Infantil 2008;8(2):151–61.

Arifeen SE, Blum LS, Hoque DME, Chowdury EK, Khan R, Black RE, et al. Integrated Management of Childhood Illness (IMCI) in Bangladesh: early findings from a cluster-randomized study. *Lancet* 2004; 364: 1595–1602.

Arifeen SE, Hoque DME, Akter T, et al. Effect of the Integrated Management of Childhood Illness strategy on childhood mortality and nutrition in a rural area in Bangladesh: a cluster randomised trial. Lancet. 2009; 374(9687):393–403.

Bhutta ZA, Das JK, Bahl R, Lawn JE, Salam RA, Paul VK, et al. Can available interventions end preventable deaths in mothers, newborn babies, and stillbirths, and at what cost? Lancet 2014; 384: 347–70.

Bickler SW, Rode H. Surgical services for children in developing countries. *Bull World Health Organ* 2002; 80: 829–835.

Bitera R, Alary M, Masse B, et al. [Quality of disease management of sexually transmitted diseases: investigation of care in six countries in West Africa]. *Sante* 2002; 12: 233–9.

Borenstein M, Hedges LV, Higgins JPT, Rothstein HR. Introduction to meta-analysis. Chichester, United Kingdom: John Wiley & Sons, Ltd., 2009.

Bosch-Capblanch X, Garner P. Primary health care supervision in developing countries. Tropical Medicine and International Health 2008:13:369-383.

Bosch-Capblanch X, Liaqat S, Garner P. Managerial supervision to improve primary health care in low- and middle-income countries. Cochrane Database of Systematic Reviews 2011;Issue 9. Art. No.: CD006413; DOI: 10.1002/14651858.CD006413.pub2.

Bose S, Oliveras E, Edson WN. 2001. How can self-assessment improve the quality of healthcare? Operations Research Issue Paper 2(4). Published for the U.S. Agency for International Development by the Quality Assurance Project, Bethesda, MD, and JHPIEGO Corporation, Baltimore, MD. Available on the Internet at address: http://www.qaproject.org/pubs/PDFs/selfassess402.pdf.

Briggs CJ, Capdegelle P, Garner P. Strategies for integrating primary health services in middle- and low-income countries: effects on performance, costs and patient outcomes. *Cochrane Database of Systematic Reviews* (4):CD003318, 2001.

Bryce J, el Arifeen S, Pariyo G, Lanata CF, Gwatkin D, Habicht JP, et al. Reducing child mortality: can public health deliver? *Lancet* 2003; 362: 159–164.

Chen L, Evans T, Anand S, Boufford JI, Brown H, Chowdhury M, et al. Human resources for health: overcoming the crisis. *Lancet* 2004; 364: 1984–90.

Chisholm D, Baltussen R, Evans DB, Ginsberg G, Lauer JA, Lim S, et al. What are the priorities for prevention and control of non-communicable diseases and injuries in sub-Saharan Africa and South East Asia? BMJ 2012;344:e586.

Ciapponi A, Lewin S, Rada G, Opiyo N, Oxman AD, Bastias G, et al. Delivery arrangements for health systems in low-income countries: an overview of systematic reviews [Protocol]. Cochrane Database of Systematic Reviews in progress, 2014.

Dieleman JL, Graves C, Johnson E, Templin T, Birger M, Hamavid H, et al. Sources and Focus of Health Development Assistance, 1990-2014. JAMA 2015; 313: 2359–68.

Dodani S, Songer T, Ahmed Z, LaPorte RE. Building Research Capacity in Developing Countries: Cost-Effectiveness of an Epidemiology Course Taught by Traditional and Video-Teleconferencing Methods in Pakistan. Telemedicine and e-Health 2012; 18: 621–628.

Drummond MF, Jefferson TO, for the BMJ Working Party on guidelines for authors and peer-reviewers of economic submissions to the *British Medical Journal*. Guidelines for authors and peer-reviewers of economic submissions to the *British Medical Journal*. *BMJ* 1996;313:275–83.

Effective Practice and Organisation of Care (EPOC). A collection of databases, web sites and journals relevant to Low- and Middle-Income Countries (LMICs). Oslo: Norwegian Knowledge Centre for the Health Services; August 2013. Available at Internet address: http://epoc.cochrane.org/sites/epoc.cochrane.org/files/uploads/LMIC%20Databases%20August%202013.pdf, accessed September 15, 2015.

Effective Practice and Organisation of Care (EPOC). Suggested risk of bias criteria for EPOC reviews. EPOC Resources for review authors. Oslo: Norwegian Knowledge Centre for the Health Services; 2015. Available at: http://epoc.cochrane.org/epoc-specific-resources-review-authors. Accessed June 19, 2015.

Egger M, Davey Smith G, Schneider M, Minder C. Bias in meta-analysis detected by a simple, graphical test. BMJ 1997; 315(7109): 629–34.

English M, Gathara D, Mwinga S, Ayieko P, Opondo C, Aluvaala J, et al. Adoption of recommended practices and basic technologies in a low-income setting. Arch Dis Child 2014; 99: 452–456.

Franco LM, Marquez L. Effectiveness of collaborative improvement: evidence from 27 applications in 12 less-developed and middle-income countries. BMJ Qual Saf 2011;20:658–65.

GBD 2013 Mortality and Causes of Death Collaborators. Global, regional, and national age-sex specific all-cause and cause-specific mortality for 240 causes of death, 1990-2013: a systematic analysis for the Global Burden of Disease Study 2013. Lancet 2015;385(9963):117–71.

Grace C, James J, Hadi Y. Selective review of work aids for alternative health care providers in developing countries. Report prepared for the Bill and Melinda Gates Foundation, June 2008.

Grimshaw JM, Thomas RE, MacLennan G, Fraser C, Ramsay C, Vale L et al. Effectiveness and efficiency of guideline dissemination and implementation strategies. *Health Technol Assess* 2004: 8(6).

Guyatt G, Oxman AD, Akl EA, Kunz R, Vist G, Brozek J, et al. GRADE guidelines: 1. Introduction—GRADE evidence profiles and summary of findings tables. Journal of Clinical Epidemiology 2011; 64: 383–394.

Hauri AM, Armstrong GL, Hutin YJ. The global burden of disease attributable to contaminated injections given in health care settings. Int J STD AIDS. 2004 15(1):7–16.

Herrera C, Ciapponi A, Rada G, Oxman AD, Lewin S, Bastias G, et al. Governance arrangements for health systems in low-income countries: an overview of systematic reviews [Protocol]. Cochrane Database of Systematic Reviews in progress, 2014.

Higgins JPT, Thompson SG. Quantifying heterogeneity in a meta-analysis. Statistics in Medicine 2002; 21: 1539-1558.

Higgins JPT, Thompson SG, Deeks JJ, Altman DG. Measuring inconsistency in meta-analyses. BMJ 2003; 327: 557-560.

Hill J, D'Mello-Guyett L, Hoyt J, van Eijk A, ter Kuile F, Webster J. Women's access and provider practices for the case management of malaria during pregnancy: a systematic review and meta-analysis. PLoS Med 2014;11:e1001688.

Holloway KA, Ivanovska V, Wagner AK, Vialle-Valentin C, Ross-Degnan D. Have we improved use of medicines in developing and transitional countries and do we know how to? Two decades of evidence. Tropical Medicine and International Health 2013; 18: 656–664.

Ivers N, Jamtvedt G, Flottorp S, Young JM, Odgaard-Jensen J, French SD, O'Brien MA, JohansenM, Grimshaw J, Oxman AD. Audit and feedback: effects on professional practice and healthcare outcomes. Cochrane Database of Systematic Reviews 2012, Issue 6. Art. No.: CD000259.

Jamtvedt G, Young JM, Kristoffersen DT, O'Brien MA, Oxman AD. Audit and feedback: effects on professional practice and health care outcomes. Cochrane Database of Systematic Reviews 2006, Issue 2. Art. No.: CD000259.

Jansen JP, Fleurence R, Devine B, Itzler R, Barrett A, Hawkins N, et al. Interpreting Indirect Treatment Comparisons and Network Meta-Analysis for Health-Care Decision Making: Report of the ISPOR Task Force on Indirect Treatment Comparisons Good Research Practices: Part 1. Value In Health 2011; 14: 417–428.

Jha A, Larizgoitia I, Audera-Lopez C, Prosopa-Plaizier N, Waters H, Bates D. The global burden of unsafe medical care: analytic modelling of observational studies. BMJ Qual Saf 2013;22:809–815.

Jones G, Steketee RW, Black RE, Bhutta ZA. Morris SS. Bellagio Child Survival Study Group. How many child deaths can we prevent this year? *Lancet* 2003; 362: 65–71.

Knebel E. 2000a. The use of manual job aids by health care providers: What do we know? Operations Research Issue Paper 1(1). Bethesda, MD: Published for the U.S. Agency for International Development by the Quality Assurance Project, Center for Human Services, University Research Co., LLC. Bethesda, MD. Available on the Internet at address: http://www.qaproject.org/pubs/PDFs/ISSUESJA.PDF.

Knebel E. 2000b. The use and effect of computer-based training: What do we know? Operations Research Issues Paper 1(2). Published for the U.S. Agency for International Development by the Quality Assurance Project, Center for Human Services, University Research Co., LLC. Bethesda, MD. Available on the Internet at address: http://www.qaproject.org/pubs/PDFs/researchcbtx.pdf.

Knebel E. 2001. The use and effect of distant education in healthcare: What do we know? Operations Research Issue Paper 2(2). Bethesda, MD: Published for the U.S. Agency for International Development by the Quality Assurance Project, Center for Human Services, University Research Co., LLC. Bethesda, MD. Available on the Internet at address: http://www.qaproject.org/pubs/PDFs/distlrnissue.pdf.

Lewin S, Munabi-Babigumira S, Glenton C *et al*. Lay health workers in primary and community health care for maternal and child health and the management of infectious diseases. *Cochrane Database of Systematic Reviews* 2010, Issue 3. Art. No.: CD004015.

Merali H, Lipsitz S, Hevelone N, Gawande A, Lashoher A, Agrawal P, et al. Audit-identified avoidable factors in maternal and perinatal deaths in low resource settings: a systematic review. BMC Pregnancy and Childbirth 2014:14:280.

Moher D, Liberati A, Tetzlaff J, Altman DG, The PRISMA Group. Preferred Reporting Items for Systematic Reviews and Meta-Analyses: The PRISMA Statement. Plos Medicine 2009; 6(7): e1000097.

Morgan R, Ensor T, Waters H. Performance of private sector health care: implications for universal health coverage. Lancet 2016; 388: 606–12.

Narasimhan V, Brown H, Pablos-Mendez A, Adams O, Dussault G, Elzinga G, Nordstrom A, Habte D, Jacobs M, Solimano G, Sewankambo N, Wibulpolprasert S, Evans T, Chen L. Responding to the global human resources crisis. *Lancet* 2004; 363: 1469–72.

Nguyen DTK, Leung KK, McIntyre L, Ghali WA, Sauve R. Does Integrated Management of Childhood Illness (IMCI) Training Improve the Skills of Health Workers? A Systematic Review and Meta-Analysis. PLoS ONE 2013; 8(6): e66030.

Opiyo N, English M. In-service training for health professionals to improve care of the seriously ill newborn or child in low and middle-income countries (Review). Cochrane Database of Systematic Reviews 2010, Issue 4. Art. No.: CD007071.

Pantoja T, Opiyo N, Ciapponi A, Dudley L, Gagnon MP, Herrera CA, et al. Implementation strategies for health systems in low-income countries: an overview of systematic reviews. Cochrane Database of Systematic Reviews 2014, Issue 5. Art.No.: CD011086. DOI: 10.1002/14651858.CD011086.

Ratanawijitrasin S, Soumerai SB, Weerasuriya K. Do national medicinal drug policies and essential drug programs improve drug use?: a review of experiences in developing countries. *Soc Sci Med* 2001; 53: 831–844.

Ross-Degnan D, Laing R, Santoso B, Ofori-Adjei, D, Lamoureux C, Hogerzeil H. Improving pharmaceutical use in primary care in developing counties: a critical review of experience and lack of experience. Presented at the International Conference on Improving Use of Medicines, Chiang Mai, Thailand, April 1997.

Rowe AK, Onikpo F, Lama M, Cokou, F, Deming MS. Management of childhood illness at health facilities in Benin: problems and their causes. *Am J Public Health* 2001; 91: 1625–1635.

Rowe AK, Rowe SY, Holloway KA, Ivanovska V, Muhe L, Lambrechts T. Does shortening the training on Integrated Management of Childhood Illness guidelines reduce its effectiveness? Results of a systematic review. Health Policy and Planning 2012; 27(3): 179–193.

Rowe AK. The effect of performance indicator category on estimates of intervention effectiveness. International Journal for Quality in Health Care 2013; 25(3): 331–9.

Saleem S, McClure E, Goudar S, Patel A, Esamai F, Garces A, et al. A prospective study of maternal, fetal and neonatal deaths in low- and middle-income countries. Bull World Health Organ 2014 Aug 1;92(8):605–12.

Shah NM, Brieger WR, Peters DH. Can interventions improve health services from informal private providers in low and middle-income countries? A comprehensive review of the literature. Health Policy and Planning 2011; 26 (4): 275–287.

Shojania KG, Ranji SR, McDonald KM, et al. Effects of quality improvement strategies for type 2 diabetes on glycemic control: a meta-regression analysis. JAMA 2006; 296: 427–40.

Sibley L, Sipe TA, Koblinsky M (2004a). Does traditional birth attendant training improve referral of women with obstetric complications: a review of the evidence. *Social Science and Medicine* 2004;59(8):1757–68.

Sibley LM, Sipe TA, Koblinsky M (2004b). Does traditional birth attendant training increase use of antenatal care? A review of the evidence. *Journal of Midwifery and Women's Health* 2004;49(4):298–305.

Siddiqi K, Newell J, Robinson M. Getting evidence into practice: what works in developing countries? Int J Qual Health Care. 2005 Oct;17(5):447–54.

Tangcharoensathien V, Mills A, Palu T. Accelerating health equity: the key role of universal health coverage in the Sustainable Development Goals. BMC Medicine 2015;13:101.

Task Force on Health Systems Research. Informed choices for attaining the Millennium Development Goals: towards an international cooperative agenda for health-systems research. *Lancet* 2004; 364: 997–1003.

Travis P, Bennett S, Haines A, Pang T, Bhutta Z, Hyder AA, Pielemeier NR, Mills A, Evans T. Overcoming health-systems constraints to achieve the Millennium Development Goals. *Lancet* 2004; 364**:** 900–6.

United Nations, 2014. Report of the Open Working Group of the General Assembly on sustainable development goals. http://www.un.org/ga/search/view_doc.asp?symbol=A/68/970. Accessed June 22, 2015.

Wagner AK et al. Segmented regression analysis of interrupted time series studies in medication use research. Journal of Clinical Pharmacy and Therapeutics 2002; 27: 299–309.

Whiting DR, Hayes L, Unwin NC. Diabetes in Africa. Challenges to health care for diabetes in Africa. Journal of Cardiovascular Risk 2003; 10:103–10.

Witter S, Fretheim A, Kessy FL, Lindahl AK. Paying for performance to improve the delivery of health interventions in low- and middle-income countries. Cochrane Database of Systematic Reviews 2012, Issue 2. Art. No.: CD007899. DOI: 10.1002/14651858.CD007899.pub2.

Wiysonge CS, Herrera C, Ciapponi A, Lewin S, Marti SG, Munabi-Babigumira S, et al. Financial arrangements for health systems in low-income countries: an overview of systematic reviews [Protocol]. Cochrane Database of Systematic Reviews, 2014.

Wootton R. Telemedicine and developing countries--successful implementation will require a shared approach. *Journal of Telemedicine and Telecare* 2001; **7** (suppl 1) :1–6.

World Bank. World Bank classification of country economies, July 2015. Available at: http://siteresources.worldbank.org/DATASTATISTICS/Resources/CLASS.XLS, accessed September 18, 2015.

World Health Organization. Interventions and strategies to improve the use of antimicrobials in developing countries. Drug Management Program, World Health Organization, Geneva. 2001. Document number: WHO/CDS/CSR/DRS/2001.9. (Plus a personal communication from J. Chalker on May 17, 2004, which included revised results tables.)

World Health Organization. The World Health Report 2006: Working together for health. World Health Organization, Geneva. 2006.

World Health Organization. Medicines use in primary care in developing and transitional countries: Fact book summarizing results from studies reported between 1990 and 2006. Geneva, World Health Organization, 2009.

World Health Organization. World Health Statistics 2014. World Health Organization, Geneva, 2014.

Zurovac, Rowe AK, Ochola SA, Noor AM, Midia B, English M, et al. Predictors of the quality of health worker treatment practices for uncomplicated malaria at government health facilities in Kenya. *Int J Epidemiol* 2004; 33: 1080–91.

Protocol annexes available from authors (email Alexander Rowe: axr@cdc.gov; Samantha Rowe: say9@cdc.gov)

- ANNEX 1. Authorship Guidelines: Faculty of Medicine, Harvard University
- ANNEX 2. Details of the search strategy
- ANNEX 3. Detailed definitions of the 13 strategy component categories
- ANNEX 4. Detailed methods for assessing risk of bias and calculating effect sizes
- ANNEX 5. Detailed methods on adjusting effect sizes and meta-analysis

Section 3. Details of the literature search (method and results) and data abstraction methods

Two literature searches were conducted: the original search (conducted from 2006–2008) and an updated search (conducted from October 2015–May 2016). Details of the original search are presented in Rowe SY et al., unpublished. Details of the updated search are presented below.

A. Methods of the updated literature search

The methods for the updated literature search were based on those used in the initial version of the HCPPR and guidance from the Cochrane Effective Practice and Organisation of Care study group (EPOC) (EPOC, 2013). Most of the updated search was conducted between October 2015 and May 2016. Some initial searching for unpublished studies was done from March–April 2014 and from June–July 2015. The updated literature search of the HCPPR had the following 11 components (see HCPPR Protocol in Section 2 for details):

1. We searched 16 electronic databases of published studies by: a) applying a filter of search terms to obtain a subset of citations that would likely include eligible studies (Box), b) ranking the resulting citations with a computer algorithm developed by the Johns Hopkins University Applied Physics Laboratory (JHU-APL), and c) screening the top 3600 citations:
    - AfricaBib databases: Africana Periodical Literature and African Women and Kenya Coast
    - BLDS British Library for Development Studies
    - CINAHL: Cumulative Index to Nursing & Allied Health Literature
    - Cochrane Library: Cochrane Central Register of Controlled Trials (CENTRAL), Cochrane Reviews, Economic Evaluations, Methods Studies, Other Reviews, Technology Assessments
    - CRD: Center for Reviews and Dissemination
    - EconLit
    - EPPI DoPHER: EPPI-Database of promoting health effectiveness reviews
    - EPPI TroPHI: EPPI-Trials Register of Promoting Health Interventions
    - ICTRP: International Clinical Trials Registry Platform
    - INRUD Medicines Use Bibliography
    - JOLIS library catalogue - International Monetary Fund, World Bank and International Finance Corporation
    - POPLINE
    - PubMed/MEDLINE
    - SCOPUS
    - Sociological Abstracts
    - WPRIM: Index Medicus for the Western Pacific

2. We searched 34 electronic databases of published studies (two of which were the same as the 16 databases described above in component 1) by: a) applying a filter of search terms to obtain a subset of citations that would like include eligible studies, and b) screening all the resulting citations. This group of databases was not ranked by the JHU-APL algorithm because the JHU-APL staff could not process the databases, given the available resources.
    - 3ie
    - AFROLIB Database
    - AIM: African Index Medicus
    - AJOL: African Journals Online

- BanglaJOL: Bangladesh Journals Online
- Bibliomap: EPPI-Centre database of health promotion research
- BVSCuba (Biblioteca Virtual En Salud De Cuba)/InfoMed
- Campbell Collaboration
- DOAJ
- EAKN: EurasiaHealth AIDS Knowledge Network
- East View Information Service Online Databases
- ELDIS
- Embase
- EPPI-Centre reviews in health systems and international development
- EPPI-Trophi
- ERIC
- Global Health and Global Health Archive
- HERDIN
- HINARI: Health InterNetwork Access to Research Initiative
- ICI: Indian Citation Index
- IDEAS Economics and Finance database (RePEc)
- IndMED
- Indonesian Publication Index
- JOLIS
- LAMJOL: Latin American Journals Online
- NepJOL: Nepal Journals Online
- Pakmedinet
- PhilJOL: Phillipines Journal Online
- PLOS One
- ProQuest Central
- SOURCE: International online resource centre on disability and inclusion
- USAID mHealth Compendium Database
- WHO-INRUD Medicines Use Database
- WHOLIS

3. We assessed 34 electronic databases of published studies that were recommended by EPOC (EPOC, 2013) (that were different from the 47 databases described above in components 1 and 2), but we decided not to search them because either: a) the database included studies primarily from a high-income country (e.g., Greece), b) studies in the database were in another database already searched, c) the database required a paid subscription and included few studies from LMICs, or d) the database (including the database's search engine) were in language that was difficult for the HCPPR Team to translate (e.g., Turkish or Vietnamese).
    - BabelMeSH
    - Biomedicina Croatica
    - Chinese Medicine Premier (Wanfang Data)
    - Chinese Scientific Journal Database
    - Collaboration for Evidence Based Healthcare in Africa (CEBHA)
    - Cochrane Library Cochrane Groups
    - Dissertation Abstracts (now called ProQuest Dissertation & Theses Global (PQDT Global))
    - EPOC Register

- EPOC Reviews
- Essential Health Links
- Global Health Gateway
- Global Health Library (GHL) of WHO
- Hellenic Ph.D. Dissertations Thesis
- HMIC
- Hrcak
- IranMedex
- KoreaMed
- Latindex
- LILACS
- Magyar Orvosi Bibliográfia (Bibliographia Medica Hungarica)
- MedCarib
- Medical Bibliography – Hippocrates
- Medical databases (Russia)
- PAHO Library Catalogue
- Panteleimon
- Psikiyatri Dizini
- Rx for Change
- SciDev Net: Science and Development Network
- Science Citation Index (now Science Citation Index Expanded)
- SSCI: Social Sciences Citation Index
- Turk MEDLINE
- Türk Tıp Veri Tabanı
- University of Zagreb Medical School Repository
- VJOL: Vietnam Journals Online

4. We searched 15 electronic databases of published studies (largely overlapping the databases in component 1, above) by: a) downloading all studies in the database, b) ranking the citations with the JHU-APL computer algorithm, and c) screening the top 2400 citations.
    - AJOL
    - BLDS British Library for Development Studies
    - Campbell Collaboration
    - Center for Reviews and Dissemination
    - Cochrane Library
    - Cumulative Index to Nursing & Allied Health Literature
    - EconLit
    - EMBASE (Jan 2015 sample)
    - EPPI DoPHER
    - EPPI TRoPHI
    - GlobalHealth (2015 sample)
    - INRUD Medicines Use Bibliography
    - JOLIS (2015 sample)
    - POPLINE (2015 sample)
    - PubMed/MEDLINE

5. We searched websites of nine scientific conferences and technical meetings to identify unpublished studies.
    - Global Health and Innovation Conference 2015
    - Global Maternal Newborn Health Conference 2013 and 2015
    - Global Symposium on Health Systems Strengthening 2010, 2012, and 2014
    - ICIUM 2011 Conference Proceedings
    - ICSHIDC: International Conference on Social Health Insurance in Developing Countries 2007
    - ISQua (International Society for Quality in Health Care) International Conference 2009-2015
    - JHPIEGO mHealth Summit 2015
    - Maternal Health Task Force Technical Meetings 2011-2014
    - Trop Med (American Society of Tropical Medicine & Hygiene) Conference 2006-2015

6. We searched websites of 44 organizations that work on HCP performance issues to identify unpublished studies.
    - ACT Consortium
    - BBC Media Action
    - Capacity Project
    - CARE Group
    - CDC websites & publications
    - Center for Global Development
    - COMDIS-HSD
    - CORDAID
    - CORE Group
    - D-tree
    - DANIDA
    - DFID
    - Engender Health
    - Gavi, The Vaccine Alliance
    - Global Fund to Fight AIDS, TB, and Malaria
    - Harvard School of Public Health
    - Health Systems Evidence
    - HealthNet TPO online library
    - HPSA Africa
    - Human Resources of Health Resource Center
    - ICCP (International Cancer Control Partnership) Portal
    - IHI: Institute for Healthcare Improvement
    - INRUD
    - JHPIEGO
    - Malaria Consortium
    - Measles and Rubella Initiative
    - mHealth Evidence
    - mpoweringhealth
    - MSH: Management Sciences for Health
    - Nuffield Center for International Health
    - PAHO

- Partners in Health
- PATH
- PHRPlus
- Population Council
- Respond Project
- Safe Injections Global Network (SIGN)
- STEPS Country Reports
- UNICEF
- USAID
- USAID Assist
- WHO
- World Bank Documents & Reports
- World Bank Open Knowledge Repository

7. We assessed seven other websites, but we decided not to search them either because: the website had not been updated since the literature search for the previous version of the HCPPR, the website was no longer active, or because we lacked the resources to search them.
    - http://www.dktinternational.org/publications-resources
    - http://healthmarketinnovations.org/programs/search
    - Annual Meeting of the Safe Injection Global Network (SIGN) 2007-2010 (e.g., URL for 2010 meeting: http://www.who.int/injection_safety/toolbox/sign2010_meeting.pdf?ua=1)
    - http://www.prime2.org/prime2/section/44.html
    - http://www.malaria.org/PSSMC/publications.html
    - http://www.malaria.org/PSSMC/reports.html
    - http://www.qaproject.org/products.html

8. We contacted 46 experts for study reports, bibliography lists, or website ideas.
    - Smisha Agarwal
    - Ray Arindam
    - Pierre Barker
    - Sebastian Bauhoff
    - Edward Broughton
    - John Chalker
    - Ingrid Chen
    - Mushtque Chowdhury
    - Sian Clarke
    - Valérie D'Acremont
    - Jishnu Das
    - Manuela De Allegri
    - Damien de Walque
    - Clara Delavallade
    - Brian DeRenzi
    - Sabine Gies
    - Christopher Gill
    - Nemat Hajeebhoy
    - Jim Heiby
    - Lisa Hirschhorn

- Kathy Holloway
- Kiersten Israel-Ballard
- Anunaya Jain
- Krishnamurthy Jayanna
- Karin Kallander
- Hnin Su Khin
- Freddy Kitutu
- Christina Marie Braüner Klokkenga
- Alain Labrique
- Sham Lal
- Christopher Lourenco
- Hema Magge
- Manoj Mohanan
- Chris Morgan
- Bright Clement Orji
- Berk Özler
- Henry Perry
- Vikrant Prabhakar
- Clotilde Rambaud-Althaus
- Arindam Ray
- Sreera Sasi
- Freddie Peter Ssengooba
- Sarah Staedke
- May Sudhinaraset
- Jakob Svensonii
- Siddhartha Swarup

9. We screened the bibliographies of 351 review articles on HCP performance that were identified from screening the results of literature search components 1-8.

10. We screened 234 other non-review-article documents that were identified from screening the results of the literature search components 1-8, or identified during the process of abstracting study reports (e.g., reports received from a study author, identified in the main study report's reference list, or identified during an internet search for more information related to a study).

11. We identified 405 potentially relevant review articles, but we decided not to screen their bibliographies because the review's inclusion criteria did not match that of our review (N=61) (e.g., the review only included non-LMIC studies), or because the review articles were published in 2006 or earlier (these "older" reviews likely had titles that we screened already in the previous version of the HCPPR) or because we lacked the resources to screen them (N=344).

Box. Search strategy for PubMed

1. exp health personnel/ not ("coroners and medical examiners"/ or veterinarians/)
2. exp Physicians/
3. (physician* or doctor*).mp.
4. exp Nurses/
5. (nurse* or nursing).mp.
6. (clinical officer* or medical officer*).mp.
7. Midwifery/
8. (midwive* or midwifery).mp.
9. Nurses' Aides/
10. (health auxiliar* or health assistant*).mp.
11. Pharmacists/
12. pharmacist*.mp.
13. Medical Laboratory Personnel/
14. (laboratory worker* or laboratory personnel).mp.
15. ((medic* or drug*) adj3 (vendor* or sell*)).mp.
16. shopkeeper*.mp.
17. Community Health Workers/
18. community health worker*.mp.
19. village health worker*.mp.
20. lay health worker*.mp.
21. birth attendant*.mp.
22. women* group*.mp.
23. Health Educators/
24. health educator*.mp.
25. health worker*.mp.
26. exp hospitals/ not Hospitals, Animal/
27. inpatient ward*.mp.
28. inpatient service*.mp.
29. exp Emergency Service, Hospital/
30. emergency department*.mp.
31. Outpatient Clinics, Hospital/
32. outpatient department*.mp.
33. clinic*.mp.
34. exp Health Facilities/
35. health facilit*.mp.
36. health post*.mp.
37. exp Pharmacy/
38. (pharmacy or pharmacies).mp.
39. (drug adj (shop* or store* or kiosk*)).mp.
40. exp Laboratories/
41. laborator*.mp.
42. ("health care provider*" or "healthcare provider*" or "private provider*").mp.
43. or/1-42
44. exp Quality Assurance, Health Care/
45. (quality adj3 care).mp.
46. Employee Performance Appraisal/
47. (employee* adj3 perform*).mp.

48. ((duty or duties) adj3 perform*).mp.
49. (task* adj3 perform*).mp.
50. (work* adj3 perform*).mp.
51. guideline/ or practice guideline/
52. (practice* adj3 guideline*).mp.
53. guidelines as topic/ or practice guidelines as topic/
54. guideline adherence/
55. (guideline* adj3 adherence).mp.
56. clinical competence/
57. clinical* competen*.mp.
58. quality improvement/
59. (quality adj3 improve*).mp.
60. ((guideline* or practice*) adj3 complian*).mp.
61. Patient Compliance/
62. patient* complian*.mp.
63. ("availability of supplies and equipment" or HCP attitudes or HCP knowledge or HCP satisfaction or supervision or assessment or case management or chemoprophylaxis or consultation time or "counseling and communication" or diagnosis or HCP documentation or referral or treatment or vaccination or morbidity or mortality or patient care-seeking or ((patient or caregiver) adj knowledge) or ((patient or community) adj attitude*) or patient satisfaction).mp.
64. or/44-63
65. infrastructure.mp.
66. financing.mp.
67. Motivation/
68. incentive*.mp.
69. government regulation/
70. regulation*.mp.
71. jurisprudence/
72. legislation, drug/
73. "drug and narcotic control"/
74. mandatory reporting/
75. Decision Making, Organizational/
76. governance.mp.
77. "Codes of Ethics"/
78. (code* adj3 ethic*).mp.
79. exp Licensure/
80. licens*.mp.
81. accreditation/
82. accredit*.mp.
83. certification/
84. certification*.mp.
85. "facility regulation and control"/
86. Decision Making/
87. (decision* adj3 making).mp.
88. management.mp.
89. Problem Solving/
90. (problem* adj3 solv*).mp.
91. exp "Organization and Administration"/
92. supervision.mp.

93. benchmarking/
94. benchmark*.mp.
95. "peer review"/ or peer review, health care/
96. (peer* adj3 review*).mp.
97. training.mp.
98. (job adj3 aid*).mp.
99. reminder systems/
100. (remind* adj3 system*).mp.
101. decision support techniques/
102. (decision* adj3 aid*).mp.
103. cell phones/ or text messaging/
104. ((cell* or mobile*) adj3 phone*).mp.
105. (text adj3 messag*).mp.
106. (audit* or Bamako Initiative* or bulletin* or collaborative improvement* or community case management* or committee* or computer or continuous quality improvement* or CQI or contracting* or data collection or drugs or education or "integrated management of childhood illness" or IMCI* or literature or maintenance or medical record* or monitoring or newsletter* or pamphlet* or performance reporting or poster* or problem-solving).mp.
107. (equipment or essential drug* or feedback* or fees or funds or group meeting or group process* or improvement collaborative* or insurance or integrate or integration or quality improvement* or recognition or registration or reimbursement or repair or standard* or system or technology or total quality management* or TQM* or assessment*).mp.
108. (scorecard* or dashboard* or "pay for performance" or detailing).mp.
109. or/65-108
110. Developing Countries/
111. (Africa or Asia or Caribbean or West Indies or South America or Latin America or Central America).mp.
112. (Afghanistan or Albania or Algeria or Angola or Armenia or Armenian or Azerbaijan or Bangladesh or Benin or Byelarus or Byelorussian or Belarus or Belorussian or Belorussia or Belize or Bhutan or Bolivia or Bosnia or Herzegovina or Hercegovina or Botswana or Brasil or Brazil or Bulgaria or Burkina Faso or Burkina Fasso or Upper Volta or Burundi or Urundi or Cambodia or Khmer Republic or Kampuchea or Cameroon or Cameroons or Cameron or Camerons or Cape Verde or Central African Republic or Chad or China or Colombia or Comoros or Comoro Islands or Comores or Mayotte or Congo or Zaire or Costa Rica or Cote d'Ivoire or Ivory Coast or Cuba or Djibouti or French Somaliland or Dominica or Dominican Republic or East Timor or East Timur or Timor Leste or Ecuador or Egypt or United Arab Republic or El Salvador or Eritrea or Ethiopia or Fiji or Gabon or Gabonese Republic or Gambia or Gaza or Georgia Republic or Georgian Republic or Ghana or Gold Coast or Grenada or Guatemala or Guinea or Guiana or Guyana or Haiti or Honduras or India or Maldives or Indonesia or Iran or Iraq or Jamaica or Jordan or Kazakhstan or Kazakh or Kenya or Kiribati or Korea or Kosovo or Kyrgyzstan or Kirghizia or Kyrgyz Republic or Kirghiz or Kirgizstan or Lao PDR or Laos or Lebanon or Lesotho or Basutoland or Liberia or Libya or Macedonia or Madagascar or Malagasy Republic or Malaysia or Malaya or Malay or Sabah or Sarawak or Malawi or Nyasaland or Mali or Marshall Islands or Mauritania or Mauritius or Agalega Islands or Mexico or Micronesia or Middle East or Moldova or Moldovia or Moldovian or Mongolia or Montenegro or Morocco or Ifni or Mozambique or Myanmar or Myanma or Burma or Namibia or Nepal or Nicaragua or Niger or Nigeria or Pakistan or Palau or Palestine or Panama or Paraguay or Peru or Philippines or Philipines or Phillipines or Phillippines or Romania or Rumania or Roumania or Rwanda or Ruanda or Saint Lucia or St Lucia or Saint Vincent or St Vincent or Grenadines or Samoa or Samoan Islands or Navigator Island or Navigator Islands or Sao Tome or Senegal or Serbia

or Montenegro or Sierra Leone or Sri Lanka or Ceylon or Solomon Islands or Somalia or South Africa or Sudan or Suriname or Surinam or Swaziland or Syria or Tajikistan or Tadzhikistan or Tadjikistan or Tadzhik or Tanzania or Thailand or Togo or Togolese Republic or Tonga or Tunisia or Turkey or Turkmenistan or Turkmen or Uganda or Ukraine or USSR or Soviet Union or Union of Soviet Socialist Republics or Uzbekistan or Uzbek or Vanuatu or New Hebrides or Vietnam or Viet Nam or West Bank or Yemen or Zambia or Zimbabwe or Rhodesia).mp.
113. ((developing or less* developed or under developed or underdeveloped or middle income or low* income or underserved or under served or deprived or poor*) adj (countr* or nation? or population? or world)).ti,ab.
114. ((developing or less* developed or under developed or underdeveloped or middle income or low* income) adj (economy or economies)).ti,ab.
115. (low* adj (gdp or gnp or gross domestic or gross national)).ti,ab.
116. (low adj3 middle adj3 countr*).ti,ab.
117. (lmic or lmics or third world or lami countr*).ti,ab.
118. transitional countr*.ti,ab.
119. or/110-118
120. randomized controlled trial.pt.
121. controlled clinical trial.pt.
122. multicenter study.pt.
123. (randomis* or randomiz* or randomly allocat* or random allocat*).ti,ab.
124. groups.ab.
125. (trial or multicenter or multi center or multicentre or multi centre).ti.
126. (intervention* or controlled or control group or compare or compared or (before adj5 after) or (pre adj5 post) or pretest or pre test or posttest or post test or quasiexperiment* or quasi experiment* or evaluat* or effect or impact or time series or time point? or repeated measur*).ti,ab.
127. or/120-126
128. exp Animals/
129. Humans/
130. 128 not (128 and 129)
131. news.pt.
132. comment.pt.
133. editorial.pt.
134. comment on.cm.
135. or/130-134
136. 127 not 135
137. 43 and 64 and 109 and 119 and 136
138. limit 137 to yr="2006 -Current"
139. 43 and 64 and 109 and 119
140. 139 not 137
141. limit 140 to yr="2006 -Current"
142. 43 and 64 and 109 and 136 and (Western Sahara or Nauru or Tuvalu).mp.
143. 142 not (138 or 141)

B. Results of the literature search

The table below summarizes the title screening and full text screening results for the 11 components of the updated literature search.

	Component	Eligibility filter applied	Ranked by JHU-APL	No. title collections	No. titles screened	No. full text screened
1	Electronic databases of published studies	✓	✓	16	3600	1290
2	Electronic databases of published studies	✓	×	34	12919	1180
3	Electronic databases of published studies not searched	×	×	34	0	0
4	Electronic databases of published studies	×	✓	15	2400	786
5	Websites of scientific conferences or technical meetings	✓	×	9	24036	1059
6	Websites of organizations that work on HCP performance	✓	×	44	44646	1135
7	Websites not searched	×	×	7	0	0
8	Documents from experts	✓	×	46	285	285
9	Bibliographies of review articles	✓	×	351	23058	2248
10	Other documents	✓	×	115	234	234
11	Review articles whose reference lists were not screened	✓	×	405	0	0
	**Total**			**1076**	**111178**	**8217**

A team of investigators and trained research assistants independently screened the results of the literature search. For the update, we screened 1076 new title collections containing 111,178 titles and reviewed the full text of 8217 titles, which after de-duplication was 7484 titles. This full-text screening identified 1445 new reports, which were added to the 824 reports identified in the previous HCPPR. The total number of reports included in the analysis of studies with true controls with at least one primary process-of-care outcome was 670 reports.

C. Data abstraction methods

A team of investigators and trained research assistants also performed data abstraction (see HCPPR Protocol for more details). Data from each study were abstracted independently by two study team members and entered into a Microsoft Access database (Microsoft Inc. Redmond, Washington). Data abstracted by the two team members were compared and discrepancies were reconciled (with consultation with a third team member, if necessary).

REFERENCES

Effective Practice and Organisation of Care (EPOC). A collection of databases, web sites and journals relevant to Low- and Middle-Income Countries (LMICs). Oslo: Norwegian Knowledge Centre for the Health Services; August 2013. Available at Internet address: http://epoc.cochrane.org/sites/epoc.cochrane.org/files/uploads/LMIC%20Databases%20August%202013.pdf, accessed September 15, 2015.

Rowe SY, et al. A systematic review of the effectiveness of strategies to improve health care provider performance in low- and middle-income countries: Methods and descriptive results of included studies. Unpublished manuscript submitted to PLOS ONE.

Section 4. Methodological details

Contents

A. Strategy component categories

B. Risk of bias categorization

C. Implementing the Grading of Recommendations Assessment, Development, and Evaluation (GRADE) system for assessing quality of evidence

D. Details of the analysis of interrupted time series (ITS) studies

E. Definitions of professional and lay health care providers

F. Effect size adjustment

G. Methods for sensitivity analyses

H. Conservative estimates of standard errors

A. Strategy component categories

Part A presents detailed definitions of the 13 strategy component categories used in the original methodology of the Health Care Provider Performance Review update. After each of the 13 strategy component categories, in square brackets, is an example dichotomous variable in the review's database that codes for the presence of the component category in a given strategy. For each of the 13 categories, there is a list of individual strategy components. After each individual strategy component, in square brackets, is an example dichotomous variable in the review's database that codes for the presence of the component in a given strategy. At the end of Part A, a detailed definition of the control group is presented as a 14th category.

Note that after analyses showed that there was no meaningful difference between the effect size distributions of low- and high-intensity training, these two categories were combined into a single "any training" category.

Abbreviations:	HCP	Health care provider
	HF	Health facility
	STG	Standard treatment guideline

1. Community support [QQA1_CommSupp]

- HCPs distributed drugs via mass community-wide campaign [q17i7_MassDrug]
- HCPs distributed immunizations via mass community-wide campaign [q17i7_MassImmun]
- Cash transfers to households conditional on behaviors or use of health services [q17L1]
- Community member received a non-conditional cash transfer [q17L9_CashTransfer]
- Community health education via group meetings (includes theater) [q17L2]
- Community health education via home visits [q17L3]
- Community health education via printed materials [q17L5]
- Community health education via radio [q17L6]
- Community health education via TV [q17L7]
- Community health education via broadcast media [q17L9_CommEduBroad]
- Community health education via children who were exposed to the health messages outside the home [q17L9_CommEduChild]
- Community health education via videos or films [q17L9_CommEduVid]
- Community health education via distribution methods other than group meetings, home visits, internet, printed materials, radio, TV, broadcast media, children, or videos [q17L9_OthCommEduc]
- Community health education via unspecified distribution methods [q17L9_unspecCommEduc]
- Emergency telephone line was set up for consultations and to clarify when to use hospitals [q17L9_EmergPhone]
- Social marketing or promotion of health goods and services [q17o2]
- HF-based HCPs provided care in community settings (outreach) [q17c13_Outreach]
- Community members were allowed to choose their own HCPs [q17L9_CommHCPchoice]
- Communities were given health-related supplies (e.g., bednets) that were not intended to directly impact HCP performance [q17r_ComHlthSuppl]
- Community health education via internet [q17L4]
- Vouchers or in-kind subsidies to households for health services or behaviors [q17L8]
- Communities were exposed to non-health-related educational messages [q17r_nonHlthEdu]
- Strategy to strengthen relationships and cooperation among community members [q17L9_CommCoop]
- Communities were given non-health-related resources (e.g., loan for household) that were neither intended to directly impact HCP performance nor support implementation of health interventions [q17r_ComNonHlthSuppl]

- Community or patient support activities (e.g., health education group meetings, home visits, printed materials) were reduced or removed [q17L9_RedComPtSupp]
- Community members conducted group meetings to discuss health-related problems in their community and to think of possible solutions [q17L9_CommGrpProc]

2. Patient support [QQA1_PatientSupp]

- Patient health education via one-on-one meetings with HCPs (excludes health education during routine consultations) [q17L9_PtEduc1on1]
- Patient health education via home visits [q17L9_PtEducHome]
- Patient health education via group meetings [q17L9_PtEducMtg]
- Patient health education via printed materials [q17L9_PtEducPrint]
- Patient selected a support person [q17L9_PtSocSupp]
- Technology-based reminders or information for patients (patient is a passive recipient). E.g., a system that reminds a patient to take medicine at home or come to a health facility for a follow-up appointment. [q17L9_Pt_ICT_Info]
- Patient health education via distribution methods other than group meetings, home visits, internet, printed materials, radio, TV, broadcast media, children, videos, or technology-based system [q17L9_OthPtEduc]

3. Strengthening infrastructure [QQA1_Infrastruc2]

- Printed management information system form or register [q17g2]
- Health services performance reporting [q17g3]
- Improved data collection system (without details on how data collection was improved) [q17g4_imprDataCol]
- Medical equipment inventory management or maintenance system [q17e1]
- Facilities repair or rebuilding [q17f2]
- Standard HF specifications were introduced [q17f4_StdHFSpecs]
- Provision of drugs [q17d12]
- Provision of medical equipment [q17e4]
- Provision of facility or new service provision point (e.g., in a school) [q17f3]
- Non-medical equipment or supplies to support implementation of health interventions [q17r_nonMedEquip]
- Structured stock ordering [q17d11]
- Essential drug list [q17d2]
- Drug therapeutic committee [q17d3]
- Standardized procurement systems [q17d8]
- At the district level, intermediary drug wholesalers were introduced [q17d13_DrWholesale]
- Improved medicine logistics [q17d13_imprMedLogis]
- Unnecessary HFs were removed (e.g., by demolishing, renting out, or changing use of HF) [q17f4_RemoveXSHF]
- A restricted drug list was implemented at HF [q17d13_RestrDrugList]
- Automatic stop order [q17d1]
- Kit systems [q17d6]
- Medical technology assessment systems [q17e2]
- Standardized medical technology list [q17e3]
- Facilities maintenance [q17f1]
- Hiring additional HCPs into pre-existing HCP cadre [q17r_HireHCP]
- HF or HCP kept records of drug purchases, sales, or stock [q17d13_MedRecKeep]
- Introduction or revision of non-medical equipment management system [q17r_nonMedMgmtSys]

4. HCP-directed financial incentives [QQA1_Financial]

- Payment/incentive: Performance-based financial incentives for staff [q17p6]
- Payment/incentive: Non-performance-based financial incentives for HCP [q17p10_FinIncent]
- Payment/incentive: Salaries [q17p9]
- Payment/incentive: Performance-based financial incentives for HFs [q17p10_HFPerfFinIncen]

- Payment/incentive: Non-performance-based financial incentives for HFs [q17p10_HFNonPerfFinInc]

5. Health system financing and other incentives [QQA1_Oth_Incent_Fin]

- The government exonerated importation taxes for essential medicines [q17d13_NoTax]
- Private sources of revenue [q17m3]
- Payment/incentive: Unspecified user fees were reduced or removed [q17m5_RedUserFee]
- Government funds were released for purchase of drugs [q17m6_GovDrugFund]
- Government funds were allocated to health sector [q17m6_GovHlthAlloc]
- Social health insurance [q17n4]
- Revolving drug funds [q17n5]
- A loan fund for HCPs or HFs was set up [q17n7_LoanFund]
- A fund for patients was set up [q17n7_PatientFund]
- Payment/incentive: Patients who were dispensed medicines were charged the cost of the drug packaging [q17p10_PkgFee]
- Payment/incentive: Consultation fee was introduced or increased [q17p2_IncConsFee]
- Payment/incentive: Consultation fee was reduced or removed [q17p2_RedConsFee]
- Payment/incentive: Fee per drug/health commodity item was introduced or increased [q17p3_IncDrugFee]
- Payment/incentive: Fee per drug/health commodity item was reduced or removed [q17p3_RedDrugFee]
- Payment/incentive: Prescription fee was introduced or increased [q17p8_IncPresFee]
- Payment/incentive: Fee per service was introduced or increased [q17p4_IncServFee]
- Payment/incentive: Fee per service was reduced or removed [q17p4_RedServFee]
- Payment/incentive: Performance-based non-financial incentives for staff [q17p7]
- Payment/incentive: Non-performance-based non-financial incentives for HCP [q17p10_NonFinIncent]
- Retail prices of essential medicines and consumables were set by government [q17q7_SetPrice]
- HF received recognition after meeting certain criteria [q17q7_HFaward]
- HF owners were given incentives [q17p10_HFownerIncent]
- Contracting-in services [q17j1]
- Contracting-out services [q17j2]
- Other contracting [q17j4_OthContract]
- Foreign countries/multinational org/bilateral organization (e.g., World Bank) donated funds to support strategies [q17m6_ForeignDonat]
- Funds were donated to support strategies, source unspecified [q17m6_UnspecFund]
- Different insurers were unified into a single insurer [q17n7_SingleInsurer]
- Contracting with incentives at contractor’s level [q17j3]
- Donations as a source of revenue for health [q17m1]
- Insurance premiums [q17m2]
- Taxes as a source of revenue for health [q17m4]
- Community-based health insurance [q17n1]
- Enterprise-based health insurance [q17n2]
- Private/voluntary health insurance [q17n3]
- Personal health savings [q17n6]
- Capitation fee [q17p1]
- Global budgets to HFs or organizations [q17p5]
- Payment/incentive: Unspecified user fees were introduced or increased [q17m5_IncUserFee]
- Reimbursement (e.g., by insurance) or subsidy to HF or HCP was revised [q17p10_RevisReimbur]
- Promotion of brand name competitor drug [q17O3_BrCompPromo] (initial version of HCPPR only)
- Pharmaceutical promotion activities that do not involve reducing drug price [q17O3_PharmaPromo] (update of HCPPR only)
- Reduction in price of a brand name competitor drug [q17p3_BrCompRed]
- Reduction in price of a generic competitor drug [q17p3_GenCompRed]
- Reduction in price of a drug named in study outcome [q17p3_StudyDrugRed]
- Managers of HCPs received performance-based incentives [q17p10_HCPMgrPerfInc]

6. Regulation and governance [QQA1_InstituApp2]

- Standard drug quality requirements were introduced [q17q7_StdDrugQual]
- Franchising or branding private HCPs [q17o1]
- Enforcement approach [q17q2]
- Accreditation [q17q3_Accreditation]
- Certification [q17q3_Certification]
- Licensing [q17q3_Licensing]
- Registration [q17q3_Registration]
- Redress mechanism [q17q4]
- Sanctions based on HCP qualifications or facility structural factor [q17q5]
- Sanctions based on services or negligence of HCP [q17q6]
- A code of ethics for HCPs or HFs was created [q17q7_CodeOfEthics]
- Public-private partnerships not involving contracting [q17i1]
- Resource control for health services given to civil society organizations [q17i4]
- Resource control for health services given to local governments [q17i5]
- Civil society organization oversight of HCPs [q17k1]
- HFs were now in control of fee revenues [q17i7_HFcontrol]
- Responsibility or authority for HCP decisions to local governments [q17i2]
- Responsibility or authority for HCP decisions to local health agencies [q17i3]
- Resource control for health services given to local public health agencies [q17i6]
- Community scorecards and community reporting [q17i7_CommScoreRep]
- Patient ratings of HCPs that are not community scorecards [q17k2]
- Patient bill of rights [q17k3]
- Banning drug or formulation [q17q1]
- Approval of a brand name competitor drug by committee at HF level or higher [q17d13_BrCompApprov]
- Approval of a generic competitor drug by committee at HF level or higher [q17d13_GenCompApprov]
- Approval of a drug named in study outcome by committee at HF level or higher [q17d13_StudyDrugApprov]
- Community members and HCPs made a formal agreement on the responsibilities of HCPs who serve the communities [q17q7_CommHCPagree]

7. Group problem solving [QQA1_GrpProbSol]

- Continuous quality improvement [q17c12]
- Collaborative Improvement [q17c13_ImprovCol]
- HCPs held meetings to discuss problems and solutions (but not formal teams) [q17c13_ProbSolv]
- Team-based problem solving [q17c3]

8. Supervision [QQA1_Supervisin]

- Benchmarking [q17c1]
- Supervision [q17c11]
- HCPs sought second opinion from peer or higher level HCP [q17c13_2ndOpinion]
- HCP received instructions from higher level HCP [q17c13_InstrHCP]
- Managers of HCPs received supervision [q17c13_MgrSup]
- Managers of HCPs received training [q17c13_MgrTrain]
- HCP received support from non-supervisory staff [q17c13_nonSupHelpHCP]
- Audit with in-person feedback [q17c4]
- Audit with written feedback [q17c5]
- Monitoring of HCP practice parameters [q17c6]
- Peer review [q17c8]
- HFs were inspected to monitor for deviations from regulations [q17q7_Inspection]
- Drug utilization review/evaluation [q17d4]
- Performance appraisal practices [q17h3]

9. Other management techniques (i.e., not group problem solving or supervision) [QQA1_OtherMgmnt2]

- Risk management [q17c10]
- HCP group process that is neither group training nor team-based problem solving [q17c13_GroupProc]
- Group meeting of HCPs and community members or patients [q17c13_GroupHCPcom]
- HCPs were given job descriptions [q17c13_HCPjobDesc]
- HCP self-assessment [q17c13_SelfAssess]
- Each HF set its own monthly performance targets [q17c13_SetTarget]
- HCPs participated in unspecified group meeting [q17c13_unspecGrpMtg]
- Reorganized management structure [q17c9]
- Hiring and selection process [q17h2]
- One or more new health services was integrated into the regular activities of HFs [q17i7_IntegServ]
- HF and HCPs were linked in a referral network [q17r_HCP_HF_link]
- Structured prescribing [q17d10]
- Change in process of care to improve accessibility of medicines [q17d13_imprMedAcces]
- Generic substitution [q17d5]
- Pre-packaging drugs [q17d7]
- Change in process of care to improve utilization of health services (including pharmaceuticals and follow-up care) [q17d13_imprMedUtil]
- Management of HCPs was decentralized to HF level (e.g., management committee was created at HF that gave staff more decision-making rights regarding management of HF) [q17c13_DecentralMgmt]
- HCPs received training on management skills (business planning, record-keeping, financial reporting, credit management, marketing) [q17c13_HCPMgmtTrain]
- Prior authorization of pharmaceuticals [q17d9]
- Operations research [q17c7]
- Regular monitoring on parameters not related to HCP clinical practice (e.g., HCP knowledge, patient outcomes) [q17c13_MonitorNonPrac]
- Reorganization of how existing HCPs are deployed (e.g., Higher-level-HF-based HCPs provided care in lower-level HFs) [q17c13_HRHReorg]
- HCPs received feedback that was not based on data collected from an audit or supervisory visit [q17c13_NonAudSupFdbk]
- Restrictions for prescribing a brand name competitor drug [q17d13_BrCompRestr]
- Restrictions on making referrals [q17c13_RestrRef]
- Disciplinary action within HCP organization [q17h1]
- Personnel development practices [q17h4]
- Promotion practices [q17h5]
- Managers of HCPs received feedback [q17c13_HCPMgrFeedbk]
- Managers of HCPs participated in group meetings [q17c13_HCPMgrMtg]

10. High-intensity training [QQA1_TrainHiInt]

- Training with a duration greater than 5 days (or ongoing training) and at least one interactive education method (i.e., clinical practice, role play, or interactive sessions) [(q17a1_InservTrain = -1 or q17a1_PreservTrain = -1 {update of HCPPR only} or q17a1 = -1 {initial version of HCPPR only}) AND (QQA1_TR_DUR >5 OR q17a1bi = -1) AND (QQA1_TR_INTERACT = 1)]
- Academic detailing (i.e., one-on-one training by an opinion leader) [q17a1_AcademDet]

11. Low-intensity training [QQA1_TrainLoInt]

- Any training not categorized as high-intensity training (above) [(q17a1_InservTrain = -1 or q17a1_PreservTrain = -1 {update of HCPPR only} or q17a1 = -1 {initial version of HCPPR only} or q17a1_AcademDet = -1 or q17r_PeerEduc = -1) AND QQA1_TrainHiInt = 0]
- Informal education of HCPs by their peers [q17r_PeerEduc]

12. Printed information or job aids for HCPs that is not an integral part of another component [QQA1_HCP_Print_Info]

- Printed pamphlet for HCP (excludes STGs) [q17b4]
- Printed patient recording form [q17b5]
- Printed STG [q17b6]
- Poster for HCP [q17b7]
- Other printed job aid for HCP (e.g., counseling cards for nurses, map of referral centers) [q17b8_OthJobaid]
- Label or stamp that HCP uses to depict dosing [q17d13_DrugLabel]
- Printed newsletter for HCP [q17b3]
- Printed literature for HCPs (not considered a job aid and not part of distinct self-study intervention) [q17b8_Literature]
- Bulletin for HCP [q17b1]
- Formulary manual [q17b2]
- Printed educational materials for HCPs to study on their own (i.e., a distinct self-study intervention) [q17b8_SelfStudy]

13. Information and communication technology (includes mHealth and eHealth) for HCPs [QQA1_HCP_ICT]

- Electronic copies of literature for HCPs (not considered a job aid; and not actively sent to HCPs, as in "q17r_HCP_ICT_remind"; and not part of distinct self-study intervention) [q17r_electronLit]
- Computerized decision aid (i.e., not simply a generic reminder sent to all HCPs) [q17c2]
- Data were collected in new or modified electronic management information system and used to improve services [q17g4_newDataCol]
- Electronic medical record system that is not a decision aid [q17g1]
- Reminders or information sent to HCPs (HCP is passive recipient). For example, SMS text message reminders to all HCP phones (not a specific reminder about a specific patient) [q17r_HCP_ICT_remind]
- Facilitates communication with a HCP peer or higher-cadre HCP (e.g., a supervisor). For example, a mobile phone-based system that a HCP can use to ask for help with a patient, facilitate a referral for a patient, or ask for more drugs or supplies if a stock-out is imminent. [q17r_HCP_com_HCP]
- Facilitates communication with between HCPs and patients. For example, a mobile phone-based system that a HCP can use to actively answer patient question or remind a patient of follow-up appointment. [q17r_HCP_com_Pt]
- Electronic educational materials for HCPs to study on their own (e.g., via cell phone or web-based) that is a distinct self-study intervention [q17r_ElectrSelfStudy]

14. Control group components

- No intervention [q17s]
- Placebo training [q17a1_PlaceboTr]
- Placebo printed STG [q17b8_PlaceboSTG]
- Placebo supervision [q17c13_PlaceboSup]
- Placebo community education group meetings [q17L9_PlaceboComMtg] (initial version of HCPPR only)
- Placebo community education home visit [q17L9_PlaceboComVis] (initial version of HCPPR only)
- Placebo community education [q17L9_PlaceboCom] (update of HCPPR only)

B. Risk of bias categorization

This method, which was designed to be an automated approach for assessing risk of bias (ROB) at the study level for use by the Health Care Provider Performance Review (HCPPR), was based on guidance from the Cochrane Effective Practice and Organisation of Care (EPOC) Group and discussions and emails with Andy Oxman and Simon Lewin in April–May 2014.

The final ROB categories are: very high ROB, high ROB, moderate ROB, and low ROB.

Note about handling ROB criteria that are unclear. For each ROB criterion that is unclear, downgrade by 0.5 ROB levels. The final number of ROB downgrades ignores fractions. Thus, it takes two unclear ROB criteria for a ROB downgrade of 1 level.

Note about studies with mixed study designs (e.g., a given study has some outcomes with an "interrupted time series (ITS) with non-randomized controls" design and some outcomes with a "pre-post with non-randomized controls" design).

- To determine the final study-level ROB category, choose the category with the highest ROB. E.g., in the example above (assuming >1 cluster per study arm), the outcomes with an "ITS with non-randomized controls" design would have a ROB category of "moderate", and the outcomes with an "pre-post with non-randomized controls" design would have a ROB category of "high". Thus, the final overall ROB category for the entire study would be "high".
- If a study with a mixed design has outcomes of different general outcome categories (e.g., process outcomes expressed as a percentage [POPs] and health impact outcomes) and one is doing an analysis of only one general outcome category (e.g., only POPs), then the above process would only apply to the POPs. E.g., in the above example (assuming >1 cluster per study arm), if all the POPs had "ITS with non-randomized controls" design, then for a POPs-only analysis, the final overall ROB category for the entire study would be "moderate".

For randomized controlled trials (RCTs), excluding controlled interrupted time series (ITS) studies

- Automatically give initial code of low ROB
- If 1 cluster per study arm, then automatically code as high ROB
- If 2–3 clusters per study arm, then downgrade by 1 ROB level
- If 4–5 clusters per study arm, then downgrade by 0.5 ROB level
- If incomplete dataset, then downgrade by 1 ROB level
- If completeness of dataset is unclear, then downgrade by 0.5 ROB level
- If imbalance in baseline outcome measurements, then downgrade by 1 ROB level
- If similarity in baseline outcome measurements is unclear, then downgrade by 0.5 ROB level
- If no baseline outcome measurements (i.e., post-only RCTs), then check for imbalance in baseline characteristics.
    - If there's an imbalance, then downgrade by 1 ROB level.
    - If it was unclear where there was an imbalance, then downgrade by 0.5 ROB level.
- If outcome was not reliable, then downgrade by 1 ROB level. Note that "not reliable" means something like self-reported health worker practices.
- If outcome reliability was unclear, then downgrade by 0.5 ROB level.

- For studies randomized at the individual level, then check for adequacy of concealment of allocation. (Note: We assumed concealment was adequate if allocation was done at the district (or some similar higher level) only, health facility only, or village only.)
    - If inadequate concealment of allocation, then downgrade by 1 ROB level.
    - If adequacy of concealment of allocation was unclear, then downgrade by 0.5 ROB level.

For non-randomized studies with controls (excluding controlled ITS studies)

- Automatically give initial code of high ROB
- If 1 cluster per study arm, then downgrade to very high ROB
- If incomplete dataset, then downgrade 1 ROB level
- If completeness of dataset is unclear, then downgrade by 0.5 ROB level
- If intervention likely to affect data collection, then downgrade 1 ROB level
- If it is unclear whether the intervention was likely to have affected data collection, then downgrade 0.5 ROB level.
- If outcome was not reliable, then downgrade by 1 ROB level. Note that “not reliable” means something like self-reported health worker practices.
- If outcome reliability was unclear, then downgrade by 0.5 ROB level.

For ITS without controls

- Automatically give initial code of moderate ROB
- If intervention not independent of other changes, then downgrade by 1 ROB level
- If it was unclear whether the intervention was independent of other changes, then downgrade by 0.5 ROB level.
- If <6 data points before or <6 data points after the intervention, then downgrade by 1 ROB level
- If intervention likely to affect data collection, then downgrade 1 ROB level
- If it is unclear whether the intervention was likely to have affected data collection, then downgrade 0.5 ROB level.
- If incomplete dataset, then downgrade 1 ROB level
- If completeness of dataset is unclear, then downgrade by 0.5 ROB level
- If outcome was not reliable, then downgrade by 1 ROB level. Note that “not reliable” means something like self-reported health worker practices.
- If outcome reliability was unclear, then downgrade by 0.5 ROB level.

For ITS with non-randomized controls

- Automatically give initial code of moderate ROB
- If control group has only 1 cluster, then reanalyze as ITS without controls (and apply ROB algorithm shown above).
- If <6 data points before or <6 data points after the intervention, then downgrade by 1 ROB level
- If intervention likely to affect data collection, then downgrade 1 ROB level
- If it is unclear whether the intervention was likely to have affected data collection, then downgrade 0.5 ROB level.
- If incomplete dataset, then downgrade 1 ROB level

- If completeness of dataset is unclear, then downgrade by 0.5 ROB level
- If outcome was not reliable, then downgrade by 1 ROB level. Note that “not reliable” means something like self-reported health worker practices.
- If outcome reliability was unclear, then downgrade by 0.5 ROB level.

For ITS with randomized controls

- Automatically give initial code of low ROB
- If control group has only 1 cluster, then reanalyze as ITS without controls (and apply ROB algorithm shown above).
- If <6 data points before or < 6 data points after the intervention, then downgrade by 1 ROB level
- If intervention likely to affect data collection, then downgrade 1 ROB level
- If it is unclear whether the intervention was likely to have affected data collection, then downgrade 0.5 ROB level.
- If incomplete dataset, then downgrade 1 ROB level
- If completeness of dataset is unclear, then downgrade by 0.5 ROB level.
- If outcome was not reliable, then downgrade by 1 ROB level. Note that “not reliable” means something like self-reported health worker practices.
- If outcome reliability was unclear, then downgrade by 0.5 ROB level.

Limitations

1. ROB assessment for non-randomized studies might be overly conservative for studies that were well done and had many clusters.
2. ROB assessment for randomized studies might be overly generous for studies with only a few clusters per arm.

Other EPOC criteria not used

1. The “blinding of outcome assessment” criterion was not used because it was assumed that a lack of blinding might only introduce bias if data collectors were prejudiced
2. The “baseline characteristics similar” criterion was not used because the HCPPR assessed this for all baseline characteristics, and an imbalance would only introduce bias if the imbalance was for factors that would have been effect modifiers. At present, it wouldn’t be feasible to go back to the studies to determine this.
3. The “contamination” criterion was not used because contamination would lead to an underestimation of effects.

C. Implementing the Grading of Recommendations Assessment, Development, and Evaluation (GRADE) system for assessing quality of evidence

Abbreviations

EPOC	Effective Practice and Organisation of Care (Cochrane Study Group)
GRADE	Grading of Recommendations Assessment, Development, and Evaluation
HCPPR	Health Care Provider Performance Review
OIS	Optimal information size
QOE	Quality of evidence
ROB	Risk of bias

C1. Assessing the quality-of-evidence (QOE) of the effectiveness of a single strategy

- **Step 1.** Use study design and risk of bias (ROB) domains to assign an initial QOE category for a given strategy
    - Based on EPOC guidance, the HCPPR used study design and other study attributes (e.g., dataset completeness, outcome reliability, etc.) to assign a ROB category for each study. The HCPPR's ROB categories are: low, moderate, high, and very high. These categories correspond to GRADE's four QOE categories [Guyatt, 2011a]. For details, see Part B of this appendix section.
    - To assign an initial QOE category for a given strategy, the study-specific ROB categories were converted into numerical scores (low = 3, moderate = 2, high = 1, and very high = 0), a simple average of the ROB scores for all study comparisons that tested the strategy was calculated, and the average ROB score was used to assign an initial QOE category (see below).
        - High QOE: $2.5 <$ average ROB score ≤ 3
        - Moderate QOE: $1.5 <$ average ROB score ≤ 2.5
        - Low QOE: $0.5 <$ average ROB score ≤ 1.5
        - Very low QOE: $0 \leq$ average ROB score ≤ 0.5

- **Step 2**. Indirectness: use the directness of study results to modify a strategy's initial QOE category
    - If a strategy had been tested by less than three study comparisons, the QOE was downgraded by one level. This step reflects the fact that a strategy's effectiveness is likely to depend on the context in which it is implemented, and thus the confidence in the magnitude of a strategy's effectiveness is related to the number of contexts in which the strategy has been tested. The choice of three study comparisons as a minimum for defining "acceptable" generalizability is acknowledged to be somewhat arbitrary.

- **Step 3**. Publication bias: assess the likelihood of publication bias and use the result to modify a strategy's QOE category
    - To identify publication bias for a given strategy, we first checked if there were results in a particular outcome group from at least 10 study comparisons per strategy (e.g., at least 10 study comparisons that each had at least one process-of-care outcome expressed as a percentage). We inspected funnel plots and used Egger's test of asymmetry (significance of $p < 0.10$) [Egger, 1997].

- If the funnel plot was asymmetric or if there was statistically significant ($p < 0.10$) evidence of asymmetry from Egger's test, then publication bias was considered "likely"; and the QOE category was reduced by one level. The HCPPR does not have an operational definition for publication bias being "very likely".

- **Step 4**. Large effect: use the summary effect size to modify a strategy's QOE category
  - If the summary effect size was 40 percentage-points or more, then the QOE category was increased by one level. This rule is based on the GRADE recommendation to increase the QOE assessment by one level if the relative risk is 2–5 [Guyatt, 2011e]. In the HCPPR, the mean baseline performance level was about 40% for process-of-care outcome expressed as a percentage. Thus, if the relative risk of a strategy were two, then mean health worker performance would increase from 40% to 80%, which is an improvement of 40 percentage points.
  - In the HCPPR, effect sizes were sometimes adjusted for contextual and methodological factors. If effect sizes for a group of outcomes were adjusted (e.g., health worker practice outcomes expressed as a percentage), then the decision to upgrade the QOE category because of an effect size of 40 percentage-points or more was based on the adjusted effect size, for consistency. If a user of the HCPPR database prefers not to use the adjusted effect sizes, the user is free to use the unadjusted effect sizes in Step 4 of this GRADE method.

- **Step 5**. Dose response: assess the likelihood of a dose-response gradient and use the result to modify a strategy's QOE category
  - If a dose-response relationship was found between the "dose" of a strategy and the strategy's effectiveness (e.g., the effectiveness of training is positively associated with the duration of training), then the QOE category was increased by one level [Guyatt, 2011e].

- **Additional cross-cutting methodologic details**
  - During the 5-step process, if upgrades or downgrades led to a QOE category higher than "high" or lower than "very low", the "out of bounds" category was considered if further upgrading or downgrading was needed. For example, if a strategy started with a "very low" level because of study design (Step 1), and then was downgraded because it was tested by less than three studies (for indirectness, in Step 2), but then was upgraded because of a very large effect size (Step 4), the final QOE category would be "very low" (assuming no change in category for Steps 3 and 5). In other words, after Step 2, the QOE category was conceptually "very, very low" (i.e., one level below "very low"). However, the final QOE category was always constrained to be one of the four GRADE levels (i.e., high, moderate, low, or very low). For example, if after Step 4, the QOE category was "high", and Step 5 led to an upgrade (because of a dose-response gradient), the final QOE category would be "high" (i.e., there is no "very high" category).

C2. Assessing the QOE of comparisons of the effectiveness of different strategies

- **Step 1**. When the effectiveness of two strategies were compared, and the evidence was a mix of indirect comparisons (i.e., comparisons of a study arm exposed to a new strategy vs. a "no new strategy" control arm, for each of the two strategies) and direct comparisons (i.e., head-to-head comparisons of the two strategies) and the results from the indirect and direct comparisons were demonstrably inconsistent, the QOE category of the comparison was downgraded by one level.

C3. GRADE-recommended attributes not used by the HCPPR to assess QOE

1. The effect of plausible confounding, which could be used to increase a strategy's QOE category [Guyatt, 2011a] was not used by the HCPPR. The effect of confounding (as used in the GRADE system) was not an element in the HCPPR data abstraction form and was not systematically assessed. It was not feasible to abstract this information retrospectively from the large number of studies in the HCPPR. Therefore, as the effect of confounding was not used, the HCPPR's QOE categorization might have underestimated the QOE for some strategies.

2. Imprecision [Guyatt, 2011b] was not used by the HCPPR because GRADE recommends judging precision with the optimal information size (OIS). OIS, however, would have been difficult to calculate because most studies in the HCPPR had correlated data (e.g., patients clustered within health facilities) but used analytic methods that did not account for this correlation. Moreover, studies did not always report sample sizes in a standard fashion (e.g., some reported numbers of patients, some reported numbers of health workers, and some reported numbers of health facilities). Therefore, as imprecision was not used, the HCPPR's QOE categorization might have overestimated the QOE for some strategies.

3. Inconsistency was not used by the HCPPR because GRADE specifies that its guidance applies to relative measures of effect [Guyatt, 2011c], and the HCPPR uses absolute measures of effect (e.g., adjusted risk difference). Therefore, as inconsistency was not used, the HCPPR's QOE categorization might have overestimated the QOE for some strategies.

References

Egger M, Davey Smith G, Schneider M, Minder C. Bias in meta-analysis detected by a simple, graphical test. BMJ 1997; 315(7109):629–34.

Guyatt 2011a. Guyatt G, Oxman AD, Akl EA, Kunz R, Vist G, Brozek J, et al. GRADE guidelines: 1. Introduction—GRADE evidence profiles and summary of findings tables. Journal of Clinical Epidemiology 2011; 64: 383–394.

Guyatt 2011b. Guyatt GH, Oxman AD, Kunz R, Brozek J, Alonso-Coello P, Rind D, et al. GRADE guidelines 6. Rating the quality of evidence—imprecision. Journal of Clinical Epidemiology 2011;64:1283–1293.

Guyatt 2011c. Guyatt GH, Oxman AD, Kunz R, Woodcock J, Brozek J, Helfand M, et al. GRADE guidelines: 7. Rating the quality of evidence—inconsistency. Journal of Clinical Epidemiology 2011;64: 1294–1302.

Guyatt 2011d. Guyatt GH, Oxman AD, Kunz R, Woodcock J, Brozek J, Helfand M. GRADE guidelines: 8. Rating the quality of evidence—indirectness. Journal of Clinical Epidemiology 2011;64: 1303–1310.

Guyatt 2011e. Guyatt GH, Oxman AD, Sultan S, Glasziou P, Aklf EA, Alonso-Coello P. GRADE guidelines: 9. Rating up the quality of evidence. Journal of Clinical Epidemiology 2011;64:1311–1316.

Rowe SY, et al. A systematic review of the effectiveness of strategies to improve health care provider performance in low- and middle-income countries: Methods and descriptive results of included studies. Unpublished manuscript submitted to PLOS ONE.

D. Details of the analysis of interrupted time series (ITS) studies

Estimation of a single ITS effect size

A. Estimation of single ITS effect size for ***1-arm ITS study***

Figure 1.

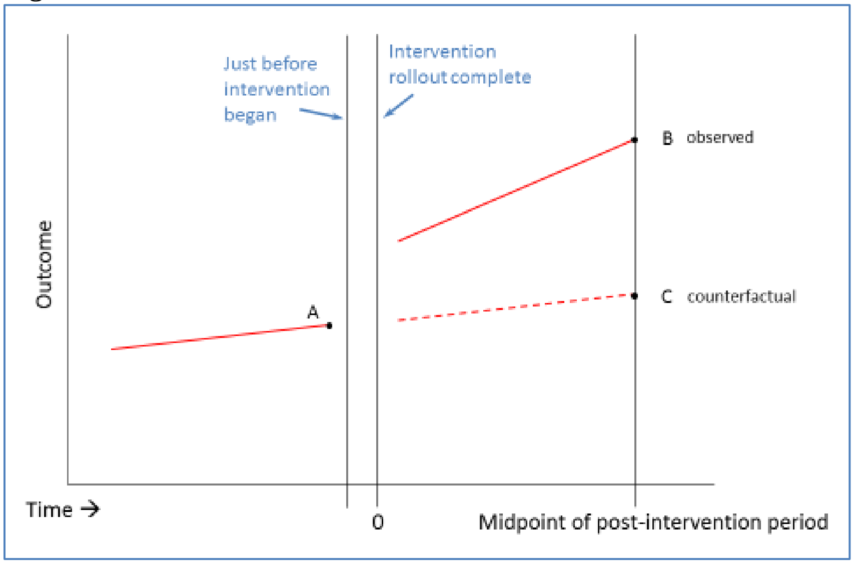

The general equation for the segmented linear regression model is

Outcome = $\beta_0 + (\beta_1)(G) + (\beta_2)(time) + (\beta_3)(Gtime)$

Where

- Outcome = either percentage or continuous, unbounded outcome
- G = 0 if before the intervention began (time<0), 1 if after roll-out is complete (time≥0)
- Time = months since intervention, equals than zero if before the intervention began, 0 or higher if after intervention roll-out is complete
- Gtime = G x time interaction

A.1. Outcome is a ***percentage***

If the outcome is a percentage, the formula for the single ITS effect size is (see Figure 1):

Single ITS effect size = B – A – (C – A) = B – C

Expressing the single ITS effect size formula in terms of the regression model coefficients, we get:

B =

$$\beta_0 + (\beta_1)(G) + (\beta_2)(time) + (\beta_3)(Gtime) = \beta_0 + (\beta_1)(1) + (\beta_2)(time) + (\beta_3)(1)(time) = \beta_0 + \beta_1 + (\beta_2 + \beta_3)(time)$$

C =
$$\beta_0 + (\beta_1)(G) + (\beta_2)(time) + (\beta_3)(Gtime) = \beta_0 + (\beta_1)(0) + (\beta_2)(time) + (\beta_3)(0)(time) = \beta_0 + (\beta_2)(time)$$

Thus, B – C =
$$\beta_0 + \beta_1 + (\beta_2 + \beta_3)(time) - (\beta_0 + (\beta_2)(time)) = \beta_1 + (\beta_3)(time)$$

Thus,

Single ITS effect size for percentage outcome for a 1-arm ITS study = $\beta_1 + (\beta_3)(time)$

A.2. Outcome is ***continuous, unbounded***

If the outcome is continuous, unbounded, the formula for the single ITS effect size is (see Figure 1):

Single ITS effect size = $\left(\frac{B-A}{A}\right) - \left(\frac{C-A}{A}\right) = \left(\frac{B-C}{A}\right)$

Expressing the single ITS effect size formula in terms of the regression model coefficients, we get:

B – C (from the previous derivation) = $\beta_1 + (\beta_3)(time)$
A = β_0

Thus, $\left(\frac{B-C}{A}\right) = \left(\frac{\beta_1 + (\beta_3)(time)}{\beta_0}\right)$

Thus,

Single ITS effect size for continuous, unbounded outcome for a 1-arm ITS study =
$$\left(\frac{\beta_1 + (\beta_3)(time)}{\beta_0}\right)$$

B. Estimation of single ITS effect size for ***2-arm ITS study***

B.1. Outcome is a ***percentage***

If the outcome is a percentage, the formula for the single ITS effect size is (see Figure 2):

Single ITS effect size = $(\text{observed} - \text{counterfactual})_{\text{intervention}}$ - $(\text{observed} - \text{counterfactual})_{\text{control}}$
= C – A – (D – A) – [E – B – (F – B)] = C – E – (D – F)

Which is equal to (J – K) in Figure 3, where the y-axis is the difference between the 2 arms' measures (intervention measure minus the control measure).

Figure 2.

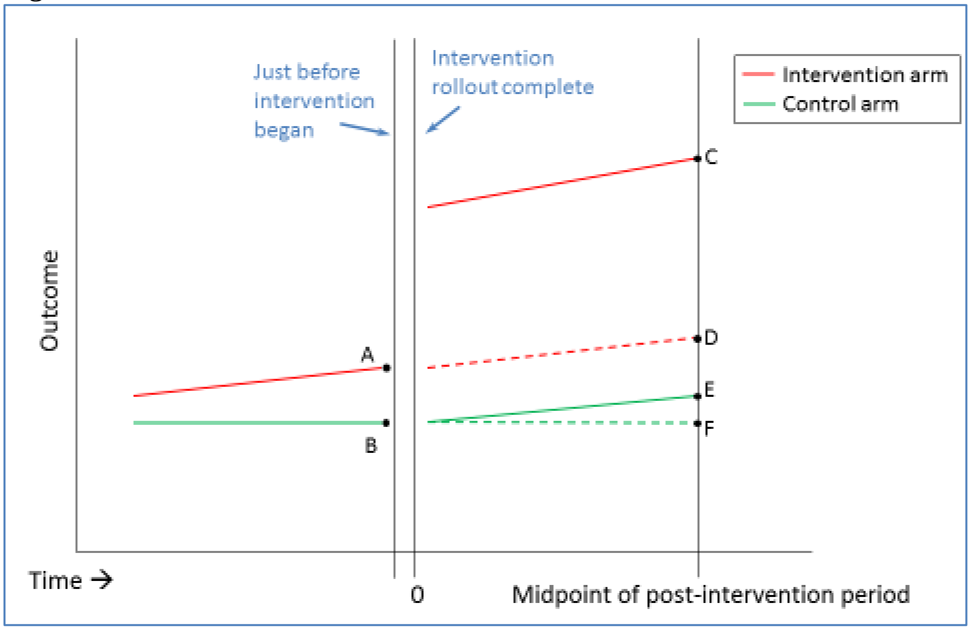

Figure 3.

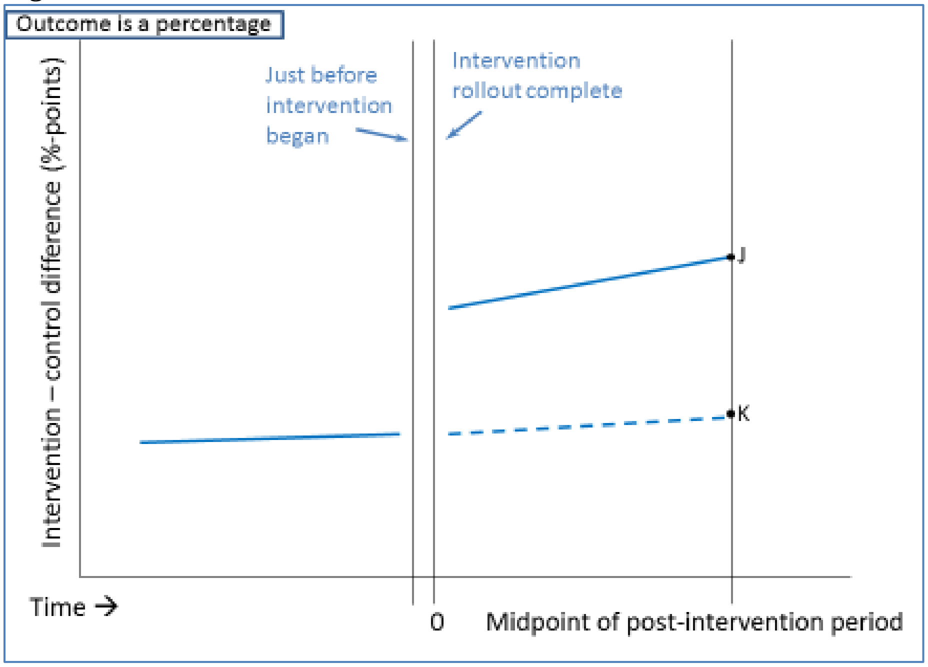

The general equation for the segmented linear regression model when the outcome is a difference between measures is

Difference = $\beta_0 + (\beta_1)(G) + (\beta_2)(time) + (\beta_3)(Gtime)$

Where

- Difference = intervention arm measure minus the control arm measure
- G = 0 if before the intervention began (time<0), 1 if after roll-out is complete (time≥0)
- Time = months since intervention, equals than zero if before the intervention began, 0 or higher if after intervention roll-out is complete
- Gtime = G x time interaction

Expressing the single ITS effect size formula in terms of the regression model coefficients, we get:

J =

$$\beta_0 + (\beta_1)(G) + (\beta_2)(time) + (\beta_3)(Gtime) = \beta_0 + (\beta_1)(1) + (\beta_2)(time) + (\beta_3)(1)(time) = \beta_0 + \beta_1 + (\beta_2 + \beta_3)(time)$$

K =

$$\beta_0 + (\beta_1)(G) + (\beta_2)(time) + (\beta_3)(Gtime) = \beta_0 + (\beta_1)(0) + (\beta_2)(time) + (\beta_3)(0)(time) = \beta_0 + (\beta_2)(time)$$

Thus, J – K =

$$\beta_0 + \beta_1 + (\beta_2 + \beta_3)(time) - (\beta_0 + (\beta_2)(time)) = \beta_1 + (\beta_3)(time)$$

Thus,

Single ITS effect size for percentage outcome for 2-arm ITS study = $\beta_1 + (\beta_3)(time)$

Where β_1 and β_3 are from the linear regression model of the difference in the outcome measures between arms.

B.2. Outcome is ***<u>continuous, unbounded</u>***

If the outcome is continuous, unbounded, the formula for the single ITS effect size is (see Figure 2):

Single ITS effect size = $\left(\frac{C-D}{A}\right) - \left(\frac{E-F}{B}\right)$

Expressing the single ITS effect size formula in terms of each arm's linear regression model coefficients, we get:

C – D = $\beta_1 + (\beta_3)(time)$ from the intervention arm's regression model
A = β_0 from the intervention arm's regression model
E – F = $\beta_1 + (\beta_3)(time)$ from the control arm's regression model
B = β_0 from the control arm's regression model

Thus, $\left(\frac{C-D}{A}\right)-\left(\frac{E-F}{B}\right)=\left(\frac{\beta_1+(\beta_3)(time)}{\beta_0}\right)_{intervention}-\left(\frac{\beta_1+(\beta_3)(time)}{\beta_0}\right)_{control}$

Thus,

Single ITS effect size for continuous, unbounded outcome for 2-arm ITS study =

$$\left(\frac{\beta_1+(\beta_3)(time)}{\beta_0}\right)_{intervention}-\left(\frac{\beta_1+(\beta_3)(time)}{\beta_0}\right)_{control}$$

Which is the difference in the single ITS effect sizes between arms (intervention minus control).

E. Definitions of professional and lay health care providers

The following table describes the attributes of professional versus lay health care providers (HCPs). We used these attributes to classify HCPs and thus determine if a study comparison was predominantly focused on lay HCPs (i.e., the study tested the effect of a strategy on the performance of lay HCPs, even if some professional HCPs were present in the study setting) or not (i.e., the study tested the effect of a strategy on the performance of only professional HCPs or a mix of professional and lay HCPs). The label "lay HCPs" was used instead of "community health worker" because sometimes lay HCPs work in health facilities, and some professional HCPs work in communities. Thus the label "community health worker" conflates the career and educational background of the HCP with the setting where he or she typically works. The classification system used in the HCPPR explicitly separates these two elements, and focuses on the former.

The attributes of professional and lay HCPs are shown in the left and right columns, respectively; although professional HCPs could sometimes have attributes of lay HCPs and vice-versa. Some HCP types were difficult to classify (e.g., Ethiopian Health Extension Workers, non-pharmacist drug vendors, informal providers in the private sector). In the primary analysis, these were classified as professional HCPs because providing health services was their career. However, as we recognize that these HCPs could be considered as either community health workers (i.e., Ethiopian Health Extension Workers, and other similar HCPs) or lay health workers (drug vendors or informal providers), we created an alternative, expanded definition of lay HCPs that included these worker types (see Section 5, Table K). We emphasize that our use of the labels "professional" and "lay" HCPs do not in any way imply that lay HCPs act unprofessionally, do not work hard, or do not take their work seriously. Rather, these labels relate to the primary career of the HCP (see row 1 of the Table below).

Table. Attributes of professional versus lay health care providers

Attribute	Professional health care providers	Lay health care providers
Career	Providing "Western" health care	Providing "Western" health care is not their career
Selection	Usually not selected by the communities that they serve	Usually selected by the specific community they serve
General education (not health related)	Typically, the minimum is a high school graduate or equivalent	Might not be a high school graduate
Pre-service training on providing health care	Most receive at least 1–2 years	Most receive less than 1 year (often a few weeks to a few months)
Full- vs. part-time work	Usually provide health care as a full-time job	Usually provide health care as a part-time job
Remuneration	Typically receive a regular salary	Typically do not receive a regular salary (but might receive other financial or non-financial incentives)
Work site	Most work in health facilities (buildings dedicated to the provision of health services)	Most do not work in health facilities (e.g., work at home or the homes of patients or clients)

F. Effect size adjustment

Contextual and methodological factors that we considered are shown in Box 1. While not an exhaustive list of all possible factors, these were the ones that we thought we could abstract from study reports with reasonably good validity. For some factors, however, while we could be fairly certain that a factor existed in a study when it was mentioned (e.g., full implementation of the strategy as planned), if the presence was not mentioned, that did not necessarily mean that the factor was absent. This uncertainty is linked to a lack of reporting standards, which is a fundamental limitation of the evidence base.

We used 3 modeling approaches to identify factors for practice outcomes, each with a somewhat different goal:

- Model 1, to identify factors that could be used to adjust effect sizes to reflect a partly standardized study context and thus reduce bias of between-strategy comparisons
- Model 2, to identify predictors of effect sizes
- Model 3, to estimate the marginal effect of each strategy component category

We attempted this set of modeling approaches for the studies focusing on professional health care providers separately from the studies focused on predominantly lay health workers, and separately for percentage versus continuous process-of-care outcomes.

Model 1: Identify factors to adjust effect sizes

- Step 1: A linear regression model was created using the SAS MIXED procedure with a random intercept to account for the clustering of effect sizes within studies. In the base model, the dependent variable was effect size, and the independent variables were 12 dummy variables that indicated the presence of the 12 strategy component categories (here, the two training categories are combined). We created a series of "univariable" models in which the variable for each potential factor was added to the base model.
- Step 2: All factor-related variables with a univariable p-value < 0.20 in Step 1 were retained in a multivariable model with a random intercept and 12 dummy variables for strategy components. Backwards elimination was done until all factor-related variables had $p < 0.025$ in multivariable model (Model A).
- Step 3: All factor-related variables with a univariable $p \geq 0.2$ in Step 1 were entered into Model A one at a time.
- Step 4: All factor-related variables with $p < 0.2$ when included in Model A were retained in a multivariable model (Model B). Backwards elimination on Model B was done until all factor-related variables had $p < 0.025$. Heretofore, this final Model B is referred to as the “adjustment model”.

Model 2: Identify predictors of effect sizes

- Step 1: A base linear regression model was created in which the dependent variable was effect size, and the independent variables were 12 dummy variables that indicated the presence of the 12 strategy component categories (here, the two training categories are combined). We created a series of "univariable" models in which the variable for each potential factor was added to the base model.
- Step 2: All factor-related variables with a univariable $p < 0.20$ in Step 1 were retained in a multivariable model with a random intercept and 12 dummy variables for strategy

components (Model A). We proceeded to the next step even if all factor-related variables did not have $p < 0.025$ in multivariable model.

- Step 3: All factor-related variables with a univariable $p \geq 0.2$ in Step 1 were entered into Model A one at a time.
- Step 4: All factor-related variables when added to Model A were retained in a multivariable model (Model B) if they met one of the following criteria:
  - The variable had a p-value < 0.05.
  - Its inclusion in Model A caused another factor (strategy component dummy variable or other covariate) to switch statistical significance (not significant to significant, or vice versa).
  - Its inclusion in Model A caused the regression coefficient of a statistically significant factor to change by >20%-points.

Model 3: Estimate marginal effect of each strategy component category

- Step 1: A base linear regression model was created in which the dependent variable was effect size, and the independent variables were 12 dummy variables that indicated the presence of the 12 strategy component categories (here, the two training categories are combined). We created a series of "univariable" models in which the variable for each potential factor was added to the base model.
- Step 2: All factor-related variables were retained in a multivariable model with a random intercept and 12 dummy variables for strategy components (Model A) if they caused either of the following when added to the base model:
  - The regression coefficient of $\geq$1 dummy variable for strategy components that had $p<0.05$ changed by $\geq$20%-points.
  - $\geq$1 dummy variable for strategy components switched statistical significance (non-significant to significant, or vice versa).

  Starting with the factor-related variable with the highest p-value, remove the variable and see if it changes by $\geq$10%-points the regression coefficient of $\geq$1 dummy variable for strategy components that had $p<0.05$, or it causes the dummy variable for a strategy component to switch statistical significance and it changes the regression coefficient by $\geq$10%-points. Retain the factor-related variable in a multivariable model (Model A) if either of these conditions are met.
- Step 3: All factor-related variables that were not retained in Step 2 were entered into Model A one at a time.
- Step 4: All factor-related variables when added to Model A were retained in a multivariable model if they caused the regression coefficient of $\geq$1 dummy variable for strategy components that had $p<0.05$ to change by $\geq$10%.

To adjust effect sizes, for each adjustment factor in the final model of Model 1 above, we subtracted the effect size-specific value of the factor from a constant (which was the mean value of the factor for all studies) and multiplied the difference by the model coefficient. This method adjusts effect sizes to reflect typical (i.e., mean) values of the factors. For example, consider the hypothetical situation in which baseline performance level was an adjustment factor. Assume the mean baseline for all studies is 50%, and the model coefficient is –0.2 (i.e., for every increase in baseline by 1 %-point, effect size decreased, on average, by 0.2 %-points). Adjusted effect size = unadjusted effect

size + [(50% – baseline of the effect size) x (–0.2)]. Thus, if an effect size is 18 %-points and the baseline is 10%, then the adjusted effect size = 18 %-points + [(50% – 10%) x (–0.2)] = 18 %-points – 8 %-points = 10 %-points. In this example, the adjustment reduces the effect size by 8 %-points because the adjustment model predicts that effect sizes with a baseline of 10% have an "advantage" of 8 %-points simply because of the low baseline value (e.g., it is easier to have larger effect sizes when the baseline is low, regardless of the strategy). The adjusted 10 %-point effect size is an estimate of what the effect size would have been if the baseline was the typical value of 50% (the mean of all studies). The adjustment of effect sizes in this manner should reduce some bias in strategy-to-strategy comparisons caused by differences in the distribution of adjustment factor values in the model. While the adjustment can potentially reduce bias, one possible limitation is that certain strategies might only be appropriate for certain settings; thus adjusting effect sizes to reflect mean values of the factors might represent an artificial situation.

The general mathematical formula to calculate the adjusted effect size was the following:

Adjusted effect size = ESrecode2 + (beta_ESbaseline2) * (mean ESbaseline2 – ESbaseline2) + (beta_Setting_Public_Only) * (mean Setting_Public_Only – Setting_Public_Only) + (beta_QQ_Asia) * (mean QQ_Asia – QQ_Asia)

Where
ESrecode2 = unadjusted effect size on -2 to +2 scale
ESbaseline2 = baseline measure for a specific unadjusted effect size on 0-1 scale (no imputed values)
Setting_Public_only = 1 if study setting is in public HFs, 0 otherwise
QQ_Asia = 1 if study country is in Asia, 0 otherwise
beta_ESbaseline2 = regression coefficient for ESbaseline2
beta_Setting_Public_only = regression coefficient for Setting_Public_only
beta_QQ_Asia = regression coefficient for QQ_Asia
mean ESbaseline2 = overall average ESbaseline2 value in dataset
mean Setting_Public_Only = proportion of effect sizes from studies set in public HFs only
mean QQ_Asia = proportion of effect sizes from studies set in Asia

Using the results from the linear regression adjustment model when HCP training was coded as either low- or high-intensity training, the formula for adjusted effect size is:

Adjusted effect size = ESrecode2 + (-0.1677)*(0.40090064 - ESbaseline2) + (0.06667)*(0.5448 - Setting_Public_Only) + (-0.05478)*(0.43078113 - QQ_Asia)

Using the results from the linear regression adjustment model when HCP training was coded as any training (either low- or high-intensity training), the formula for adjusted effect size is:

Adjusted effect size = ESrecode2 + (-0.1681)*(0.40090064 - ESbaseline2) + (0.06775)*(0.5448 - Setting_Public_Only) + (-0.05292)*(0.43078113 - QQ_Asia)

Box 1. Study attributes that are potentially associated with strategy effect sizes

1. No. of components in strategy, regardless of what they are
2. The strategy was fully implemented as planned (yes vs. no)
3. New STG in study setting (yes vs. no)
4. New HCP (i.e., new cadre of HCP was created, such as CHWs)
5. HCP type (only CHWs vs. not only CHWs)
6. New HCP responsibility (i.e., existing HCP was given a new responsibility)
7. Baseline outcome value
8. Study setting
    a. Low-income vs. middle income country
    b. WHO Region (Africa vs. Latin America vs. Asia [Southeast Asia or Western Pacific] vs. other regions)
    c. Rural-urban (rural only vs. all other categories)
    d. For "not CHW only studies": public HFs only vs. other settings
9. Study design
    a. RCT vs. other designs
    b. ITS without a control group vs. other designs
10. Risk of bias
    a. Low or moderate vs. high
    b. Data complete (yes vs. no or unclear)
11. Follow-up time
12. Whether or not HCPs helped develop strategy
13. Strategy designed to overcome specific performance problem in study area
14. Outcome choice
15. Calendar year of mid-point of field work
16. Published vs. grey literature
17. Patient/client attributes
    a. Age (only < 5 years old vs. other categories, which might include under-5s)
    b. Health condition (communicable diseases, non-communicable diseases, reproductive health, other)
18. Study sample size of clusters
19. Data collection methods (record review vs. other methods)
20. Outcome interpretability (easy vs. potentially difficult)

G. Methods for sensitivity analyses

Four sensitivity analyses were performed to examine the influence of study bias, limited numbers of comparisons, different economic and health facility settings, and the effects of weighting and adjustment.

1. First, we analyzed only studies with a low or moderate risk of bias (Section 5, Tables L1 and L2).

2. Second, for strategies tested by at least three comparisons each with large effect sizes, we examined whether the large effect sizes could be due to limited contextual diversity (Section 5, Table M). This analysis involved broadening the strategy definition to include strategies with the same set of core components but with other components allowed. For example, for the strategy group problem solving only, the sensitivity analysis involved a calculation of the effectiveness of group problem solving with or without additional strategy components. We assumed that adding components did not reduce effectiveness. If the median MES of a strategy group with a broadened definition was lower than that of the original (narrower) strategy group definition, then bias seemed likely. We used the number of countries where a given strategy group was tested as an index of contextual diversity.

3. The third sensitivity analysis was designed to better characterize the contexts in which a strategy might be more effective. For strategies tested by at least three comparisons each, we stratified results according to the level of resources and development of the setting where the study was conducted, which had two categories: low (i.e., studies from low-income countries not done in only hospital settings and studies from only rural settings in middle-income countries) and moderate (i.e., hospital-only studies from low-income countries and studies from middle-income countries in which the setting was not only rural) (Section 5, Table N1). Our classification of a country's economy as low versus middle income was based on the World Bank's economy category for that country in 2015 (the year of our literature search). For the small number of multi-country studies in both low- and middle-income countries, the categorization of level of resources assumed the study was from a middle-income country. We also performed an analysis in which the low versus moderate resource categories were more simply defined as low- and middle-income countries, respectively (Section 5, Table N2).

4. Fourth, strategy effectiveness was estimated with unadjusted and unweighted effect sizes (Section 5, Table O).

Although not a formal, a priori sensitivity analysis, an additional analysis was performed. As we recognize that some HCPs that we classify as professional could be considered as either community health workers (e.g., Ethiopian Health Extension Workers, and other similar HCPs) or lay health workers (drug vendors or informal providers), we created an alternative, expanded definition of lay HCPs that included these health worker types (see Section 5, Table K).

H. Conservative estimates of standard errors

H1. Estimation of variance of unadjusted effect sizes for non-ITS outcomes

Variance of unadjusted effect sizes for non-ITS ***percentage*** outcomes

Start with the formula for effect size for a pre-post study with controls:

Effect size = ES = (PY2 – PX2) – (PY1 – PX1)

where

PX1 = proportion with outcome A at baseline for study arm 1
PX2 = proportion with outcome A at baseline for study arm 2
PY1 = proportion with outcome A at follow-up for study arm 1
PY2 = proportion with outcome A at follow-up for study arm 2

Assuming statistical independence between study arms, then:

Variance of ES = Var(ES) = Var(PY2 – PX2) + Var(PY1 – PX1)

In general, the formula for Var(PY – PX) for any study arm is:

Var(PY – PX) = Var(PX) + Var(PY) – 2*Cov(PX,PY)

where
Var(PX) = PX * (1 – PX)/n1
Var(PY) = PY * (1 – PY)/n2
Cov(PX, PY)
= (correlation coefficient between baseline and follow-up) * square root[Var(PX) * Var(PY)]

and

PX = proportion with outcome A at baseline
PY = proportion with outcome A at follow-up
n1 = effective sample size at baseline = number of people observed / design effect at baseline
n2 = effective sample size at follow-up = number of people observed / design effect at follow-up
design effect
= {(intra-class correlation coefficient) * ((no. of people observed) / (no. of clusters observed) – 1)} + 1

Note that the estimate of effective sample size (i.e., n1 and n2) might often not be the true effective sample size of a study, because study reports rarely included the information needed to estimate the true effective sample size. We used several conservative assumptions to produce a conservative estimation of effective sample size, which was needed for the meta-analyses. Thus, it might be more

accurate to consider the effective sample size estimates as “analysis weights” (or a component of the final analysis weight).

We assumed a value of 0.40 for the intra-class correlation coefficient. This ICC value, which was designed to be conservative, was chosen to be twice the median ICC value from a study of ICCs [Rowe, 2002]. For a simple validity check, we found ICC estimates of 0.071 to 0.388 from a study in the review [Rowe, 2009], which suggested that a value of 0.4 would be a reasonable conservative estimate.

We assumed a value of 0.50 for the correlation coefficient between the baseline and follow-up measures. This correlation value was based on the methodology of Cochrane systematic reviews, which suggests if the outcome of interest is something where there is little evidence available to be able to assign a correlation coefficient, a value of 0.5 should be assumed (see http://heart.cochrane.org/Files/Handling%20continuous%20variables.pdf).

Thus,

Var(PX – PY) = PX * (1 – PX)/n1 + PY * (1 – PY)/n2 – 2 * 0.5 * square root[Var(PX) * Var(PY)]

In conclusion, sum together the Var(PX – PY) for each study arm to calculate the variance of the effect size.

<u>Variance of unadjusted effect sizes for non-ITS ***continuous, unbounded*** outcomes</u>

For non-ITS continuous, unbounded outcomes, we estimated the variance of the unadjusted effect size if the variance of each study arm’s outcome measure was available.

Estimation of variance of effect size for 2-arm, non-ITS, pre-post study with controls

Start with the formula for effect size for a pre-post study with controls:

$$effect\ size = (relative\ difference_i - relative\ difference_c) \times 100$$
$$= \left(\frac{Followup_i - Baseline_i}{Baseline_i} - \frac{Followup_c - Baseline_c}{Baseline_c}\right) \times 100$$

Let $X = \frac{Followup_i - Baseline_i}{Baseline_i} = \frac{Followup_i}{Baseline_i} - 1$

And $Y = \frac{Followup_c - Baseline_c}{Baseline_c} = \frac{Followup_c}{Baseline_c} - 1$

Use the delta method to estimate the variance of the effect size, assuming the control and intervention groups are statistically independent (i.e., covariance between them is zero):

$$Var[effect\ size] = Var[(X-Y)(100)] = (100^2)Var[X-Y] = (100^2)(Var[X] + Var[Y])$$

Then use the delta method to estimate Var[X] and Var[Y] (the variances of the relative baseline-to-follow-up differences for the intervention and control groups, respectively):

$$\begin{aligned}
Var[X] = Var\left[\frac{Followup_i}{Baseline_i} - 1\right]& \\
&= Var[Followup_i]\left(\frac{\partial\left(\frac{Followup_i}{Baseline_i} - 1\right)}{\partial Followup_i}\right)^2 \\
&+ \ Var[Baseline_i]\left(\frac{\partial\left(\frac{Followup_i}{Baseline_i} - 1\right)}{\partial Baseline_i}\right)^2 \\
&+ 2Cov(Baseline_i, Followup_i)\left(\frac{\partial\left(\frac{Followup_i}{Baseline_i} - 1\right)}{\partial Followup_i}\right)\left(\frac{\partial\left(\frac{Followup_i}{Baseline_i} - 1\right)}{\partial Baseline_i}\right) \\
&= Var[Followup_i]\left(\frac{1}{Baseline_i}\right)^2 \\
&+ Var[Baseline_i]\left((-1)(Followup_i)(Baseline_i)^{-2}\right)^2 \\
&+ 2Cov(Baseline_i, Followup_i)\left(\frac{1}{Baseline_i}\right)\left((-1)(Followup_i)(Baseline_i)^{-2}\right)
\end{aligned}$$

Where

$$\begin{aligned}
Cov(Baseline_i, Followup_i) &= \left(\rho_{Baseline_i, Followup_i}\right)\sqrt{(Var[Baseline_i])(Var[Followup_i])} \\
&= (0.5)\sqrt{(Var[Baseline_i])(Var[Followup_i])}
\end{aligned}$$

We assumed a value of 0.50 for the correlation coefficient between the baseline and follow-up measures. This correlation value was based on the methodology of Cochrane systematic reviews, which suggests if the outcome of interest is something where there is little evidence available to be able to assign a correlation coefficient, a value of 0.5 should be assumed (see http://heart.cochrane.org/Files/Handling%20continuous%20variables.pdf).

Similarly for Var[Y]:

$$Var[Y] = Var\left[\frac{Followup_c}{Baseline_c} - 1\right]$$

$$= Var[Followup_c]\left(\frac{\partial\left(\frac{Followup_c}{Baseline_c} - 1\right)}{\partial Followup_c}\right)^2 + Var[Baseline_c]\left(\frac{\partial\left(\frac{Followup_c}{Baseline_c} - 1\right)}{\partial Baseline_c}\right)^2$$

$$+ 2Cov(Baseline_C, Followup_c)\left(\frac{\partial\left(\frac{Followup_c}{Baseline_c} - 1\right)}{\partial Followup_C}\right)\left(\frac{\partial\left(\frac{Followup_c}{Baseline_c} - 1\right)}{\partial Baseline_C}\right)$$

$$= Var[Followup_C]\left(\frac{1}{Baseline_c}\right)^2 + Var[Baseline_c]\left((-1)(Followup_c)(Baseline_c)^{-2}\right)^2$$

$$+ 2Cov(Baseline_c, Followup_c)\left(\frac{1}{Baseline_c}\right)\left((-1)(Followup_c)(Baseline_c)^{-2}\right)$$

$$= Var[Followup_C]\left(\frac{1}{Baseline_c}\right)^2 + Var[Baseline_c]\left((-1)(Followup_c)(Baseline_c)^{-2}\right)^2$$

$$+ 2(0.5)\sqrt{(Var[Baseline_c])(Var[Followup_c])}\left(\frac{1}{Baseline_c}\right)\left((-1)(Followup_c)(Baseline_c)^{-2}\right)$$

In conclusion, sum together Var[X] and Var[Y] and multiply that sum by 100^2 to calculate the variance of the effect size.

Estimation of variance of effect size for 2-arm, non-ITS, post-only study with controls

Start with the formula for effect size for a post-only study with controls:

$$effect\ size = \left(\frac{Followup_i - Followup_c}{Followup_c}\right) \times 100$$

Let $X = \frac{Followup_i - Followup_c}{Followup_c} = \frac{Followup_i}{Followup_c} - 1$

$$Var[effect\ size] = Var[(X)(100)] = (100^2)Var[X]$$

Then use the delta method to estimate Var[X] (the variance of the relative difference between the follow-up measures for the intervention and control groups):

$$Var[X] = Var\left[\frac{Followup_i}{Followup_c} - 1\right]$$

$$= Var[Followup_i]\left(\frac{\partial\left(\frac{Followup_i}{Followup_c} - 1\right)}{\partial Followup_i}\right)^2$$

$$+ \ Var[Followup_C]\left(\frac{\partial\left(\frac{Followup_i}{Followup_c} - 1\right)}{\partial Followup_r}\right)^2$$

$$+ 2Cov(Followup_i, Followup_C)\left(\frac{\partial\left(\frac{Followup_i}{Followup_c} - 1\right)}{\partial Followup_i}\right)\left(\frac{\partial\left(\frac{Followup_i}{Followup_C} - 1\right)}{\partial Followup_c}\right)$$

$$= Var[Followup_i]\left(\frac{1}{Followup_c}\right)^2$$

$$+ Var[Followup_C]\left((-1)(Followup_i)(Followup_c)^{-2}\right)^2$$

$$+ 2(0)\left(\frac{1}{Followup_c}\right)\left((-1)(Followup_i)(Followup_c)^{-2}\right)$$

$$= Var[Followup_i]\left(\frac{1}{Followup_C}\right)^2$$

$$+ Var[Followup_C]\left((-1)(Followup_i)(Followup_c)^{-2}\right)^2$$

In conclusion, multiply Var[X] by 100^2 to calculate the variance of the effect size.

<u>How we dealt with missing components of variance of unadjusted effect sizes</u>

The variance formulas above assume that the following data are available for each study arm: a baseline and follow-up measure and numbers of people and clusters observed at those points. For example, for a two-armed study, 12 data elements are needed to calculate the variance of an effect size: two baseline measures, two follow-up measures, four sample sizes of people observed, and four sample sizes of clusters observed. However, studies did not always report all of these data. If any component in the variance formula was missing for an effect size, the variance for that effect size could not be calculated. When a data element was missing, we first attempted to estimate the data using information from study reports or authors; if this was not successful, we attempted to impute data using summary measures of all percentage process-of-care outcomes from the entire dataset of percentage process-of-care studies; missing data elements from studies identified through the original literature search were imputed based on the dataset from the original literature search, while missing data elements from studies identified through the updated literature search were imputed based on the dataset from the updated literature search. If this was not successful, we imputed

plausible values based on our general understanding of the literature. The following describes our estimation or imputation process.

For percentage outcomes in post-only studies with randomized controls, we assumed that: (1) the ‘hypothetical’ baseline measures for all study arms were equal to the follow-up measure for the control arm, (2) the ‘hypothetical’ number of people (or clusters) observed at baseline for the control arm was equal to that at follow-up for the control arm, and (3) the ‘hypothetical’ number of people (or clusters) observed at baseline for the intervention arm was equal to that at follow-up for the intervention arm.

For pre-post studies with controls, within each study group, if the number of people (or clusters) observed at baseline was missing, we assumed that the number of people (or clusters) observed at baseline was equal to the number of people (or clusters) at follow-up. If the number of people (or clusters) observed at follow-up was missing, we assumed that the number of people (or clusters) observed at follow-up was equal to the number of people (or clusters) at baseline.

For percentage process-of-care outcomes, if the number of people observed or the number of clusters was missing at a particular time point (e.g., baseline or follow-up), we imputed a value for the sample size of people observed (or sample size of clusters) that equaled the overall average sample size from the database of percentage process-of-care outcomes at that time point.

If neither the proportion with the outcome at baseline (PX) nor the proportion with the outcome at follow-up (PY) were reported, but the baseline-to-follow-up difference in the proportion (PY – PX) was reported, we assumed the proportions were centered at 50%, and imputed a value for the proportion as:

- baseline measure = 50% - 2*(half-width of baseline-to-follow-up difference for each arm
- follow-up measure = 50% + 2*(half-width of baseline-to-follow-up difference for each arm

For percentage process-of-care outcomes, if the variance of a baseline measure was missing or zero, it was predicted from a linear regression model in which the dependent variable was the variance of the baseline measure, and the independent variable was the inverse of the effective sample size at baseline.

For percentage process-of-care outcomes, if the variance of a follow-up measure was missing or zero, it was predicted from a linear regression model in which the dependent variable was the variance of the follow-up measure, and the independent variable was the inverse of the effective sample size at follow-up.

References

Rowe AK, Lama M, Onikpo F, Deming MS. Design effects from a health facility cluster survey in Benin. International Journal for Quality in Health Care 2002;14:521–523.

Rowe AK, Onikpo F, Lama M, Osterholt DM, Rowe SY, Deming MS. A multi-faceted intervention to improve health worker adherence to Integrated Management of Childhood Illness guidelines in Benin. American Journal of Public Health 2009; 99: 837–846.

H2. Estimation of variance of unadjusted effect sizes for ITS outcomes

Estimation of variance of single ITS effect size for ***1-arm ITS study***

Outcome is a ***percentage***

To derive the general formula for calculating the variance of the single ITS effect size for a percentage outcome, the delta method is used, with

Z = $\beta_1 + (\beta_3)(time)$

If we take the first derivative of Z with respect to each regression coefficient β_1 and β_3, and treat time as a constant (i.e., time just has one value, the midpoint of the post-intervention period), we get:

$$\frac{\partial Z}{\partial \beta_1} = 1 \qquad \frac{\partial Z}{\partial \beta_3} = time$$

Using the delta method approximation, we estimate Var(Z):

$$Var(Z) \approx [Var(\beta_1)]\left(\frac{\partial Z}{\partial \beta_1}\right)^2 + [Var(\beta_3)]\left(\frac{\partial Z}{\partial \beta_3}\right)^2 + 2[Cov(\beta_1, \beta_3)]\left(\frac{\partial Z}{\partial \beta_1}\right)\left(\frac{\partial Z}{\partial \beta_3}\right)$$

$$= [Var(\beta_1)](1)^2 + [Var(\beta_3)](time)^2 + 2[Cov(\beta_1, \beta_3)](1)(time)$$

$$= Var(\beta_1) + [Var(\beta_3)](time)^2 + 2[Cov(\beta_1, \beta_3)](time)$$

Thus, the variance of a single ITS effect size for a percentage outcome in a 1-arm ITS study is:

$$Var(\beta_1) + [Var(\beta_3)](time)^2 + 2[Cov(\beta_1, \beta_3)](time)$$

Each of the variance and covariance estimates are available from the regression model (e.g., SAS PROC AUTOREG variance-covariance matrix output). Time is equal to the midpoint in months of the post-intervention period (i.e., the last months since intervention divided by 2).

Outcome is ***continuous, unbounded***

To derive the general formula for calculating the variance of the single ITS effect size for a continuous, unbounded outcome, the delta method is used, with

Z = $\left(\frac{\beta_1 + (\beta_3)(time)}{\beta_0}\right)$

If we take the first derivative of Z with respect to each regression coefficient β_0, β_1, and β_3, and treat time as a constant (i.e., time just has one value, the midpoint of the post-intervention period), we get:

$$\frac{\partial Z}{\partial \beta_0} = \frac{(-1)[\beta_1 + (\beta_3)(time)]}{\beta_0^{\,2}} \qquad \frac{\partial Z}{\partial \beta_1} = \frac{1}{\beta_0} \qquad \frac{\partial Z}{\partial \beta_3} = \frac{time}{\beta_0}$$

Using the delta method approximation, we estimate Var(Z):

$$Var(Z) \approx$$

$$[Var(\beta_0)]\left(\frac{\partial Z}{\partial \beta_0}\right)^2 + [Var(\beta_1)]\left(\frac{\partial Z}{\partial \beta_1}\right)^2 + [Var(\beta_3)]\left(\frac{\partial Z}{\partial \beta_3}\right)^2 + 2[Cov(\beta_0,\beta_1)]\left(\frac{\partial Z}{\partial \beta_0}\right)\left(\frac{\partial Z}{\partial \beta_1}\right)$$

$$+\,2[Cov(\beta_0,\beta_3)]\left(\frac{\partial Z}{\partial \beta_0}\right)\left(\frac{\partial Z}{\partial \beta_3}\right) + 2[Cov(\beta_1,\beta_3)]\left(\frac{\partial Z}{\partial \beta_1}\right)\left(\frac{\partial Z}{\partial \beta_3}\right)$$

$$= [Var(\beta_0)]\left(\frac{(-1)[\beta_1 + (\beta_3)(time)]}{\beta_0^{\,2}}\right)^2 + [Var(\beta_1)]\left(\frac{1}{\beta_0}\right)^2 + [Var(\beta_3)]\left(\frac{time}{\beta_0}\right)^2$$

$$+\,2[Cov(\beta_0,\beta_1)]\left(\frac{(-1)[\beta_1 + (\beta_3)(time)]}{\beta_0^{\,2}}\right)\left(\frac{1}{\beta_0}\right) + 2[Cov(\beta_0,\beta_3)]\left(\frac{(-1)[\beta_1 + (\beta_3)(time)]}{\beta_0^{\,2}}\right)\left(\frac{time}{\beta_0}\right)$$

$$+\,2[Cov(\beta_1,\beta_3)]\left(\frac{1}{\beta_0}\right)\left(\frac{time}{\beta_0}\right)$$

Thus, the variance of a single ITS effect size for a continuous, unbounded outcome in a 1-arm ITS study is:

$$[Var(\beta_0)]\left(\frac{(-1)[\beta_1 + (\beta_3)(time)]}{\beta_0^{\,2}}\right)^2 + [Var(\beta_1)]\left(\frac{1}{\beta_0}\right)^2 + [Var(\beta_3)]\left(\frac{time}{\beta_0}\right)^2$$

$$+\,2[Cov(\beta_0,\beta_1)]\left(\frac{(-1)[\beta_1 + (\beta_3)(time)]}{\beta_0^{\,2}}\right)\left(\frac{1}{\beta_0}\right) + 2[Cov(\beta_0,\beta_3)]\left(\frac{(-1)[\beta_1 + (\beta_3)(time)]}{\beta_0^{\,2}}\right)\left(\frac{time}{\beta_0}\right)$$

$$+\,2[Cov(\beta_1,\beta_3)]\left(\frac{1}{\beta_0}\right)\left(\frac{time}{\beta_0}\right)$$

Each of the variance and covariance estimates are available from the regression model (e.g., SAS PROC AUTOREG variance-covariance matrix output). Time is equal to the midpoint in months of the post-intervention period (i.e., the last months since intervention divided by 2).

Estimation of variance of single ITS effect size for ***<u>2-arm ITS study</u>***

Outcome is a ***<u>percentage</u>***

To derive the general formula for calculating the variance of the single ITS effect size for a percentage outcome from a 2-arm ITS study, the delta method is used, with

Z = $\beta_1 + (\beta_3)(time)$

Where the regression coefficients β_1 and β_3 come from the linear regression model of the difference between the 2 arms' measures (intervention measure minus the control measure), and time is treated as a constant (i.e., time just has one value, the midpoint of the post-intervention period). If we take the first derivative of Z with respect to each regression coefficient β_1 and β_3, we get:

$$\frac{\partial Z}{\partial \beta_1} = 1 \qquad \frac{\partial Z}{\partial \beta_3} = time$$

Using the delta method approximation, we estimate Var(Z):

$$Var(Z) \approx$$

$$[Var(\beta_1)]\left(\frac{\partial Z}{\partial \beta_1}\right)^2 + [Var(\beta_3)]\left(\frac{\partial Z}{\partial \beta_3}\right)^2 + 2[Cov(\beta_1, \beta_3)]\left(\frac{\partial Z}{\partial \beta_1}\right)\left(\frac{\partial Z}{\partial \beta_3}\right)$$

$$= [Var(\beta_1)](1)^2 + [Var(\beta_3)](time)^2 + 2[Cov(\beta_1, \beta_3)](1)(time)$$

$$= Var(\beta_1) + [Var(\beta_3)](time)^2 + 2[Cov(\beta_1, \beta_3)](time)$$

Thus, the variance of a single ITS effect size for a percentage outcome from a 2-arm ITS study is:

$$Var(\beta_1) + [Var(\beta_3)](time)^2 + 2[Cov(\beta_1, \beta_3)](time)$$

Where the variances and covariance are available from the linear regression model of the <u>difference</u> in the outcome measures between arms (e.g., SAS PROC AUTOREG variance-covariance matrix output). Time is equal to the midpoint in months of the post-intervention period (i.e., the last months since intervention divided by 2).

Outcome is ***<u>continuous, unbounded</u>***

For the variance of the single ITS effect size for a continuous, unbounded outcome from a 2-arm ITS study, the delta method is used, with

Z = (Single ITS effect size)$_{intervention}$ – (Single ITS effect size)$_{control}$

If we take the first derivative of Z with respect to each arm's single ITS effect size, we get:

$$\frac{\partial Z}{\partial SingleITSeffectsize_{\text{int}}} = 1 \qquad \frac{\partial Z}{\partial SingleITSeffectsize_{con}} = -1$$

Using the delta method approximation, we estimate Var(Z):

$Var(Z) \approx$

$$[Var(SingleITSeffectsize_{\text{int}})]\left(\frac{\partial Z}{\partial SingleITSeffectsize_{\text{int}}}\right)^2$$

$$+[Var(SingleITSeffectsize_{con})]\left(\frac{\partial Z}{\partial SingleITSeffectsize_{con}}\right)^2$$

$$+2[Cov(SingleITSeffectsize_{\text{int}}, SingleITSeffectsize_{con})]\left(\frac{\partial Z}{\partial SingleITSeffectsize_{\text{int}}}\right)\left(\frac{\partial Z}{\partial SingleITSeffectsize_{con}}\right)$$

$$= [Var(SingleITSeffectsize_{\text{int}})](1)^2 + [Var(SingleITSeffectsize_{con})](-1)^2 + 0$$

$$= Var[Single\ ITS\ effect\ size_{int}] + Var[Single\ ITS\ effect\ size_{con}]$$

Since the covariance between the single ITS effect sizes for each arm is equal to 0 because the 2 arms are assumed to be statistically independent.

Thus, the variance of a single ITS effect size for a continuous, unbounded outcome from a 2-arm ITS study is:

$$Var[Single\ ITS\ effect\ size_{int}] + Var[Single\ ITS\ effect\ size_{con}]$$

Which is the sum of the variances of the single ITS effect size for each arm.

H3. Estimation of variance of adjusted effect sizes

Variance of adjusted effect size = Var[ESrecode2] +
(A^2) Var[beta_ESbaseline2] +
(B^2) Var[beta_Setting_Public_only] +
(C^2) Var[beta_QQ_Asia] +
(2)(A)(B) Covar[beta_ESbaseline2, beta_Setting_public_only] +
(2)(A)(C) Covar[beta_ESbaseline2, beta_QQ_Asia] +
(2)(B)(C) Covar[beta_Setting_Public_Only, beta_QQ_Asia]
Where
Adjusted effect size = adjusted effect size on -2 to +2 scale
ESrecode2 = unadjusted effect size on -2 to +2 scale
ESbaseline2 = baseline measure for a specific unadjusted effect size on 0-1 scale (no imputed values)
Setting_Public_only = 1 if study setting is in public HFs, 0 otherwise
QQ_Asia = 1 if study setting is in Asia, 0 otherwise
beta_ESbaseline2 = regression coefficient for ESbaseline2
beta_Setting_Public_only = regression coefficient for Setting_Public_only
beta_QQ_Asia = regression coefficient for QQ_Asia
mean ESbaseline2 = overall average ESbaseline2 value in dataset
mean Setting_Public_Only = proportion of effect sizes from studies set in public HFs only
mean QQ_Asia = proportion of effect sizes from studies set in Asia
A = (mean ESbaseline2 – ESbaseline2)
B = (mean Setting_Public_Only – Setting_Public_Only)
C = (mean QQ_Asia – QQ_Asia)
Var[ESrecode2] = Variance of unadjusted effect size, which is a function of the effective sample sizes and measures at baseline and post-intervention.

The following linear regression adjustment model was run in SAS PROC MIXED with the “method=reml” procedure option, the “covb” model option, and the random intercept option to estimate regression coefficients and their variance-covariance matrix:

ESrecode2 = intercept + QQ_CommSupp + QQ_PatientSupp + QQ_Infrastruc2 + QQ_Financial + QQ_Oth_Incent_Fin + QQ_InstituApp2 + QQ_GrpProbSol + QQ_Supervisin + QQ_OtherMgmnt2 + QQ_AnyTrain + QQ_HCP_Print_Info + QQ_HCP_ICT + ESbaseline2 + Setting_Public_Only + QQ_Asia

Where the first “QQ_” variables were the 12 dummy variables for the 12 strategy components.

Using the results from the linear regression adjustment model, the formula for variance of the adjusted effect size is:

Variance of adjusted effect size = Var[ESrecode2] + A*A*0.000996+ B*B*0.000451 + C*C*0.000404 + 2*A*B*(-0.00002) + 2*A*C*(-0.00008) + 2*B*C*(-0.00007)
Where A = (0.40090064 - ESbaseline2), B = (0.5448 - Setting_Public_Only), and
C = (0.43078113 - QQ_Asia)

H4. Estimation of variance of median effect sizes

The formula for the variance of a median effect size (Var(MES)) was based on the theory of order statistics (http://www.math.ntu.edu.tw/~hchen/teaching/LargeSample/notes/noteorder.pdf) and was the following:

Var(MES) = π * (sum of the variances of effect sizes within each comparison) / (2 * number of effect sizes within each comparison * number of effect sizes within each comparison)

Section 5. Additional results

Contents

Table A. General study attributes

Table B. Number of studies stratified by publication status and risk of bias category

Table C. Study setting: places where services were delivered, who owns or operates the service delivery points, and types of health care providers

Table D. Health conditions addressed by studies in the review

Table E. Categories of all 1688 effect sizes from all 337 included studies

Table F. Distribution of strategy component categories across all intervention arms

Table G. Time trends in study attributes

Table H1. Model to estimate strategy component effectiveness and identify contextual and methodological factors associated with effect size for practice outcome expressed as a percentage (including both professional health care providers and lay health workers): Low- and high-intensity training analyzed separately

Table H2. Model to estimate strategy component effectiveness and identify contextual and methodological factors associated with effect size for practice outcome expressed as a percentage (including both professional health care providers and lay health workers): All training combined into one category

Table I. The effectiveness of strategies to improve health care provider performance for studies of professional health care providers with at least one practice outcome with low- and high-intensity training analyzed separately

Table J1. The effectiveness of strategies to improve health care provider performance for studies of professional health care providers with at least one practice outcome: strategies tested by one or two study comparisons

Table J2. The effectiveness of strategies to improve health care provider performance for studies predominantly of lay or community health workers with at least one practice outcome: strategies tested by one or two study comparisons

Table K. The effectiveness of strategies to improve health care provider performance for studies predominantly of lay or community health workers with at least one practice outcome: sensitivity analysis

Table L1. The effectiveness of strategies to improve health care provider performance for studies of professional health care providers with at least one practice outcome and that had a low or moderate risk of bias

Table L2. The effectiveness of strategies to improve health care provider performance for studies of lay or community health workers with at least one practice outcome and that had a low or moderate risk of bias

Table M. Assessing strategy effectiveness by broadening strategy definitions to increase contextual and implementation diversity

Table N1. The effectiveness of strategies tested by at least three study comparisons each, for studies of professional health care providers with at least one practice outcome expressed as a percentage: stratification by low-resource versus moderate-resource setting where the study was conducted

Table N2. The effectiveness of strategies tested by at least three study comparisons each, for studies of professional health care providers with at least one practice outcome expressed as a percentage: stratification by low- versus middle-income country where the study was conducted

Table O. Sensitivity and secondary analyses on the effectiveness of strategies to improve health care provider performance for studies of professional health care providers with at least one practice outcome expressed as a percentage: meta-analyses of adjusted and unadjusted effect sizes, median analysis of unadjusted weighted effect sizes, and median analysis of unadjusted unweighted effect sizes

Table P. The effectiveness of strategies evaluated in equivalency studies with a gold standard control group

Figure A. Study follow-up times for 317 studies that reported study duration

Figure B. Effect size for practice outcome expressed as a percentage as a function of baseline performance level

Figure C1. Effect size distributions for practice outcomes expressed as a percentage as a function of the number of components in the strategy

Figure C2. Effect size distributions for practice outcomes expressed as a percentage as a function of the number of component categories in the strategy

Table A. General study attributes

Study attribute	All studies (N=337)
Number of study arms	
1	50 (14.8%)
2	247 (73.3%)
3	31 (9.2%)
4	9 (2.7%)
Total number of study arms across all studies	673
Total number of comparisons across all studies	
Strategy vs. true (no intervention) control group	379 (99.5%)
Strategy vs. placebo control group	2 (0.5%)
Total number of effect sizes across all studies	
Median number of effect sizes per study (range)	2 (1–106)
Median number of effect sizes per comparison (range)	2 (1–106)
Study designs	
Pre-post study with randomized controls	112 (33.2%)
Pre-post study with non-randomized controls	108 (32.1%)
Interrupted time series with no controls	51 (15.1%)
Post-only study with randomized controls	48 (14.2%)
Interrupted time series with non-randomized controls	10 (3.0%)
Interrupted time series with randomized controls	8 (2.4%)
Economy of country where study was done	
Low income	133 (39.5%)
Lower-middle income	117 (34.7%)
Upper-middle income	84 (24.9%)
Combination of lower-middle and upper-middle income	3 (0.9%)
Risk of bias	
Low	54 (16.0%)
Moderate	84 (24.9%)
High	98 (29.1%)
Very high	101 (30.0%)

Table continued on next page.

Table A. General study attributes, continued.

Study attribute	All studies (N=337)
WHO region where study was conducted	
Africa	140 (41.5%)
Southeast Asia	68 (20.2%)
America	53 (15.7%)
Western Pacific	44 (13.1%)
Eastern Mediterranean	23 (6.8%)
Europe	9 (2.7%)
Year of publication (or date of document for unpublished reports), by decade	
2010 or later (latest year was 2017)[a]	143 (42.4%)
2000–2009	141 (41.8%)
1990–1999	49 (14.5%)
1980–1989	3 (0.9%)
Before 1980 (earliest year was 1974)	1 (0.3%)
Data collection methods (multiple responses allowed per study)	
Record or chart review	220 (65.3%)
Interview with patient or patient's caretaker	90 (26.7%)
Observation of HCP-patient interaction	57 (16.9%)
Interview with HCP	44 (13.1%)
Questionnaire for HCP (any administration method)	29 (8.6%)
Simulated client	28 (8.3%)
Questionnaire for patient or patient's caretaker	20 (5.9%)
Observation of facility	18 (5.3%)
Physical exam of patient by study team	16 (4.7%)
Observation of HCP practices not involving real patients	6 (1.8%)
Interview with administrator	5 (1.5%)
Case scenario	4 (1.2%)
Observation of patient's behaviors	3 (0.9%)
Exam for HCP (e.g., written test for HCP)	3 (0.9%)
Observation of patient's home	2 (0.6%)
HCP self-assessment	2 (0.6%)
Questionnaire for an administrator	1 (0.3%)
Questionnaire for supervisor	1 (0.3%)

Table continued on next page.

Table A. General study attributes, continued.

Study attribute	All studies (N=337)
Urban vs. rural study setting	
Urban +/- peri-urban areas	110 (32.6%)
Rural areas only	83 (24.6%)
Mix of urban and rural areas	78 (23.2%)
Town +/- rural areas	16 (4.8%)
Peri-urban areas only	10 (3.0%)
Mix of peri-urban and town areas	1 (0.3%)
Mix of peri-urban, town, and rural areas	1 (0.3%)
Unclear or not stated	38 (11.3%)
Data available on strategy cost or other economic evaluation (from either the study reports or responses from investigators)	125 (37.1%)

[a] Many reports from 2016 and all from 2017 either were originally identified as unpublished, but were published by the time of the analysis, or were reports that authors or experts provided after the formal literature search had ended.

Table B. Number of studies stratified by publication status and risk of bias category[a]

Risk of bias category	Publication status		Total
	At least 1 study report published in scientific journal No. (row %)	No study reports published in scientific journal No. (row %)	
Low	50 (92.6)	4 (7.4)	54
Moderate	70 (83.3)	14 (16.7)	84
High	82 (83.7)	16 (16.3)	98
Very high	82 (81.2)	19 (18.8)	101
Total	284 (84.3)	53 (15.7)	337

Footnote.

[a] The Cochran–Mantel–Haenszel test for the association between publication status and risk of bias category yields a p-value of 0.11.

Table C. Study setting: places where services were delivered, who owns or operates the service delivery points, and types of health care providers

Study attribute	All studies (N=337)
Places where services were delivered (multiple responses allowed)	
Outpatient health facility	196 (58.2%)
Hospital outpatient department	94 (27.9%)
Hospital inpatient wards	88 (26.1%)
Household or community setting	47 (14.0%)
Pharmacy	27 (8.0%)
Drug shop	21 (6.2%)
Non-hospital health facility inpatient ward	14 (4.2%)
School	4 (1.2%)
Laboratory	3 (0.9%)
Site in transit to hospital or health facility	1 (0.3%)
Other outpatient setting	5 (1.5%)
Who owns or operates the place where services were delivered (multiple responses allowed per study)	
Public or government	247 (73.3%)
Private, for profit	58 (17.2%)
Community	51 (15.1%)
Private, not for profit	34 (10.1%)
Private, profit status unknown or not reported	17 (5.0%)
Public-private partnership	8 (2.4%)
Unclear or not reported	16 (4.8%)
Type of health care providers (multiple responses allowed per study)	
Physician	186 (55.2%)
Nurse	153 (45.4%)
Midwife	69 (20.5%)
Lay health worker	57 (16.9%)
Nurse aide	56 (16.6%)
Clinical officer	42 (12.5%)
Pharmacist	39 (11.6%)
Pharmacist assistant or non-pharmacist drug vendor	39 (11.6%)
Paramedic or unspecified non-physician	39 (11.5%)
Health educator or information officer	22 (6.5%)
Laboratorian	21 (6.2%)
Midwife aide	17 (5.0%)
Student	7 (2.1%)
Health care providers with nonstandard labels who had 1-3 years pre-service training	1 (0.3%)
Health care provider, type unspecified	24 (7.1%)
Lay health worker was the predominant type of health care provider	24 (7.1%)

Table D. Health conditions addressed by studies in the review

Health condition (multiple responses allowed per study)	No. of studies with at least one effect size related to the health condition, among all 337 studies
Multiple (or all) health conditions	91 (27.0%)
Pregnancy	58 (17.2%)
Acute respiratory infections	47 (13.9%)
HIV/AIDS +\- other sexually transmitted diseases	43 (12.8%)
Malaria	41 (12.2%)
Diarrhea	36 (10.7%)
Reproductive health (not pregnancy related)	28 (8.3%)
Tuberculosis	22 (6.5%)
Newborn health conditions	19 (5.6%)
Non-communicable diseases (not covered by other categories, such as asthma)	17 (5.0%)
General medicine use	16 (4.7%)
Malnutrition	12 (3.6%)
Sexually transmitted diseases (HIV/AIDS not specifically included)	11 (3.3%)
Vaccine-preventable illnesses	11 (3.3%)
Other infectious diseases (not covered by other categories, such as appendicitis)	8 (2.4%)
Child health (not covered by other categories, such was well-baby checks)	7 (2.1%)
Heart disease	4 (1.2%)
Mental health	4 (1.2%)
Infection prevention	4 (1.2%)
Dental health	4 (1.2%)
Hypertension	3 (0.9%)
Non-malaria parasite	1 (0.3%)
Injuries and trauma	1 (0.3%)

Table E. Categories of all 1688 effect sizes from all 337 included studies

Outcome	HCP practice outcome scale		Totals for percentage and continuous outcomes combined
	Percentage	Continuous	
Assessment	59 studies<br>71 comparisons<br>251 effect sizes	8 studies<br>8 comparisons<br>9 effect sizes	63 studies<br>75 comparisons<br>260 effect sizes
Case management[a]	75 studies<br>81 comparisons<br>163 effect sizes	2 studies<br>2 comparisons<br>2 effect sizes	77 studies<br>83 comparisons<br>164 effect sizes
Chemoprophylaxis	5 studies<br>5 comparisons<br>6 effect sizes	0 studies<br>0 comparisons<br>0 effect sizes	5 studies<br>5 comparisons<br>6 effect sizes
Consultation time	1 studies<br>1 comparisons<br>1 effect sizes	13 studies<br>14 comparisons<br>30 effect sizes	14 studies<br>15 comparisons<br>31 effect sizes
Counseling and communication	67 studies<br>74 comparisons<br>360 effect sizes	5 studies<br>5 comparisons<br>21 effect sizes	69 studies<br>76 comparisons<br>381 effect sizes
Diagnosis	18 studies<br>19 comparisons<br>25 effect sizes	21 studies<br>22 comparisons<br>28 effect sizes	38 studies<br>40 comparisons<br>53 effect sizes
Dispensing time by HCP	0 studies<br>0 comparisons<br>0 effect sizes	1 studies<br>1 comparisons<br>1 effect sizes	1 studies<br>1 comparisons<br>1 effect sizes
Documentation by HCP	24 studies<br>26 comparisons<br>55 effect sizes	2 studies<br>2 comparisons<br>4 effect sizes	24 studies<br>26 comparisons<br>59 effect sizes
Information accessed by HCP	2 studies<br>2 comparisons<br>6 effect sizes	0 studies<br>0 comparisons<br>0 effect sizes	2 studies<br>2 comparisons<br>6 effect sizes
Non-health-related task by HCP	1 studies<br>1comparisons<br>2 effect sizes	0 studies<br>0 comparisons<br>0 effect sizes	1 studies<br>1 comparisons<br>2 effect sizes
Patient dignity	3 studies<br>3 comparisons<br>10 effect sizes	0 studies<br>0 comparisons<br>0 effect sizes	3 studies<br>3 comparisons<br>10 effect sizes
Patient visit by HCP	3 studies<br>3 comparisons<br>6 effect sizes	3 studies<br>3 comparisons<br>3 effect sizes	6 studies<br>6 comparisons<br>9 effect sizes
Referral	20 studies<br>23 comparisons<br>39 effect sizes	6 studies<br>8 comparisons<br>9 effect sizes	25 studies<br>30 comparisons<br>48 effect sizes

Outcome	HCP practice outcome scale		Totals for percentage and continuous outcomes combined
	Percentage	Continuous	
Reporting time by HCP	1 studies<br>2 comparisons<br>2 effect sizes	1 studies<br>1 comparisons<br>2 effect sizes	2 studies<br>3 comparisons<br>4 effect sizes
Treatment	165 studies<br>195 comparisons<br>531 effect sizes	50 studies<br>56 comparisons<br>87 effect sizes	181 studies<br>211 comparisons<br>618 effect sizes
Universal precautions by HCP	7 studies<br>7 comparisons<br>27 effect sizes	0 studies<br>0 comparisons<br>0 effect sizes	7 studies<br>7 comparisons<br>27 effect sizes
Vaccination	3 studies<br>3 comparisons<br>3 effect sizes	3 studies<br>3 comparisons<br>6 effect sizes	6 studies<br>6 comparisons<br>9 effect sizes
Total	287 studies<br>328 comparisons<br>1486 effect sizes	105 studies<br>115 comparisons<br>202 effect sizes	337 studies<br>381 comparisons<br>1688 effect sizes

Footnote.

HCP = health care provider

[a] Outcomes that include multiple steps of the case-management pathway (e.g., correct diagnosis and treatment).

Table F. Distribution of strategy component categories across all intervention arms

Strategy component	No. (%) of intervention arms that included the strategy component (N = 381 study arms)
Any training (e.g., high-intensity and low-intensity combined; includes informal education of HCPs by their peers, academic detailing)	240 (63.0%)
Supervision (e.g., improving routine supervision)	162 (42.5%)
Management techniques, excluding group problem solving and supervision (e.g., changing processes of care to improve utilization of health services)	91 (23.9%)
Strengthening infrastructure (e.g., provision of drugs)	88 (23.1%)
Community support (e.g., community health education)	60 (15.8%)
Health system financing and other incentives (e.g., user fees)	52 (13.7%)
Group problem solving (eg., continuous quality improvement)	45 (11.8%)
Information and communication technology (e.g., text messages)	37 (9.7%)
Printed or electronic information or job aid for HCPs that is not an integral part of another component[a]	34 (8.9%)
Patient support (e.g., patient health education)	33 (8.7%)
Regulation and governance (e.g., accreditation)	29 (7.6%)
HCP-directed financial incentives (e.g., salary)	18 (4.7%)

Footnotes.

HCP = Health care provider.

[a] Other strategy components (especially training) often include printed information for HCPs; and in these cases, the printed information was not considered a separate component. This category includes printed or electronic information for HCPs when the information is not an integral part of another component. For example, a strategy that only consists of distributing pamphlet to HCPs.

Table G. Time trends in study attributes

Study attribute	Year of publication					Percent annual change	P-value of annual change
	1970s	1980s	1990s	2000	2010s[a]		
All studies							
No. of studies	1	3	49	141	143		
Mean no. of studies per year	0.1	0.3	4.9	14.1	20.5	13.4[b]	<0.0001
Risk of bias							
Low risk of bias							
No. of studies (% of all studies)	0 (0)	1 (33.3)	7 (14.3)	20 (14.2)	26 (18.2)	1.7[c]	0.44
Mean no. of studies per year	0	0.1	0.7	2.0	3.5	11.8	<0.0001
Low or moderate risk of bias							
No. of studies (% of all studies)	0 (0)	2 (66.7)	18 (36.7)	53 (37.6)	65 (45.5)	0.9	0.59
Mean no. of studies per year	0	0.2	1.8	5.3	9.0	11.2	<0.0001
Country income classification, as defined by the World Bank (2015)							
Low income							
No. of studies (% of all studies)	0 (0)	0 (0)	18 (36.7)	65 (46.1)	50 (35.0)	1.0	0.47
Mean no. of studies per year	0	0	1.8	6.5	6.5	14.6	<0.0001
Lower-middle income							
No. of studies (% of all studies)	1 (100)	2 (66.7)	26 (53.1)	44 (31.2)	47 (32.9)	–5.8	0.0003
Mean no. of studies per year	0.1	0.2	2.6	4.4	6.7	10.1	<0.0001
Upper-middle income							
No. of studies (% of all studies)	0 (0)	1 (33.3)	6 (12.2)	34 (24.1)	46 (32.2)	6.0	0.003
Mean no. of studies per year	0	0.1	0.6	3.4	7.3	15.8	<0.0001
Geographic region, as defined by the World Health Organization							
Africa							
No. of studies (% of all studies)	0 (0)	0 (0)	12 (24.5)	44 (31.2)	84 (58.7)	11.1	<0.0001
Mean no. of studies per year	0	0	1.2	4.3	10.3	16.3	<0.0001
America							
No. of studies (% of all studies)	1 (100)	1 (33.3)	13 (26.5)	27 (19.2)	11 (7.7)	–7.5	0.0001
Mean no. of studies per year	0.1	0.1	1.3	2.7	1.8	8.5	<0.0001
Eastern Mediterranean							
No. of studies (% of all studies)	0 (0)	0 (0)	1 (2.0)	9 (6.4)	13 (9.1)	5.1	0.16
Mean no. of studies per year	0	0	0.1	0.9	2.2	16.6	<0.0001
Europe							
No. of studies (% of all studies)	0 (0)	0 (0)	1 (2.0)	4 (2.8)	4 (2.8)	0.8	0.88
Mean no. of studies per year	0	0	0.1	0.4	0.5	10.8	0.01
Southeast Asia							
No. of studies (% of all studies)	0 (0)	2 (66.7)	19 (38.8)	34 (24.1)	13 (9.1)	–8.4	<0.0001
Mean no. of studies per year	0	0.2	1.9	3.4	1.8	9.2	<0.0001
Western Pacific							
No. of studies (% of all studies)	0 (0)	0 (0)	3 (6.1)	24 (16.9)	18 (12.6)	1.3	0.58
Mean no. of studies per year	0	0	0.3	2.3	2.8	14.2	<0.0001

Table G, continued. Time trends in study attributes

Study attribute	Year of the study's mid-point of data collection					Percent annual change	p-value of annual change
	1970s	1980s	1990s	2000s	2010s[a]		
Ownership of the place where services were delivered							
Public							
No. of studies (% of all studies)	0 (0)	1 (33.3)	37 (75.5)	99 (70.2)	110 (76.9)	2.7	0.12
Mean no. of studies per year	0	0.1	3.7	9.9	16.0	13.7	<0.0001
Private[d]							
No. of studies (% of all studies)	0 (0)	1 (33.3)	10 (20.4)	47 (33.3)	44 (30.8)	2.5	0.16
Mean no. of studies per year	0	0.1	1.0	4.7	5.2	12.2	<0.0001
Community setting							
No. of studies (% of all studies)	1 (100)	2 (66.7)	9 (18.4)	19 (13.5)	20 (14.0)	–2.3	0.27
Mean no. of studies per year	0.1	0.2	0.9	1.9	2.5	9.3	<0.0001
Publication in a scientific journal[e]							
No. of studies (% of all studies)	1 (100)	3 (100)	37 (75.5)	114 (80.9)	129 (90.2)	4.7	0.02
Mean no. of studies per year	0.1	0.3	3.7	11.4	18.8	12.0	<0.0001

Footnotes.

[a] For analyses of number of studies per year, this category includes 2000–2015. For analyses of the proportion of studies with a particular attribute, this category includes 2000–2017.

[b] Annual change in the number of studies per year. For example, between 1970 and 2015, the number of studies per year increased by 13.4% per year.

[c] Annual change in the odds that a study will have the attribute. For example, between 1970 and 2015, the odds of a study having a low risk of bias increased by 1.7% per year.

[d] Includes private for profit, private non-profit, private with profit status unknown, and public-private partnership.

[e] For studies with results in multiple reports, a study was considered published if at least one report was published in a scientific journal.

Table H1. Model to estimate strategy component effectiveness and identify contextual and methodological factors associated with effect size for practice outcome expressed as a percentage (including both professional health care providers and lay health workers): Low- and high-intensity training analyzed separately

Factor	Model 1, to identify contextual factors to adjust effect sizes		Model 2, to identify predictors of effect sizes		Model 3, to estimate the marginal effect of each strategy component category	
	Parameter estimate (95% CI)	P-value[a]	Parameter estimate (95% CI)	P-value[a]	Parameter estimate (95% CI)	P-value[a]
Intercept	7.8 (2.2, 13.3)	0.01	4.6 (-2.0, 11.3)	0.18	7.8 (2.2, 13.3)	0.01
Dummy variables[b] that each code for a strategy component category						
Community support	0.2 (-5.1, 5.5)	0.94	1.8 (-3.4, 7.0)	0.50	0.2 (-5.1, 5.5)	0.94
Patient support	-4.0 (-9.9, 1.8)	0.18	-4.4 (-10.4, 1.5)	0.15	-4.0 (-9.9, 1.8)	0.18
Strengthening infrastructure	-1.2 (-7.4, 4.9)	0.70	-0.8 (-6.8, 5.2)	0.80	-1.2 (-7.4, 4.9)	0.70
HCP-directed financial incentives	6.2 (-6.2, 18.6)	0.33	7.4 (-4.3, 19.2)	0.22	6.2 (-6.2, 18.6)	0.33
Health system financing and other incentives	2.8 (-3.2, 8.7)	0.36	1.3 (-4.3, 7.0)	0.65	2.8 (-3.2, 8.7)	0.36
Regulation and governance	2.5 (-4.5, 9.6)	0.48	3.1 (-4.0, 10.3)	0.39	2.5 (-4.5, 9.6)	0.48
Group problem solving	13.5 (5.5, 21.4)	0.001	12.4 (4.5, 20.3)	0.002	13.5 (5.5, 21.4)	0.001
Supervision	1.1 (-1.8, 3.9)	0.45	1.3 (-1.6, 4.1)	0.38	1.1 (-1.8, 3.9)	0.45
Other management techniques	3.3 (-1.9, 8.5)	0.21	3.0 (-2.3, 8.3)	0.27	3.3 (-1.9, 8.5)	0.21
High-intensity training	4.8 (-0.6, 10.3)	0.08	3.8 (-1.8, 9.3)	0.18	4.8 (-0.6, 10.3)	0.08
Low-intensity training	7.0 (2.0, 12.1)	0.01	6.3 (1.2, 11.4)	0.02	7.0 (2.0, 12.1)	0.01
Printed information or job aid for HCPs	-0.6 (-6.3, 5.0)	0.83	-1.2 (-6.8, 4.5)	0.69	-0.6 (-6.3, 5.0)	0.83
Information and communication technology for HCPs	-2.6 (-8.8, 3.6)	0.41	-3.1 (-9.3, 3.0)	0.31	-2.6 (-8.8, 3.6)	0.41
Contextual and methodological factors (all mean-centered)						
Baseline performance level	-0.2 (-0.2, -0.1)	<0.0001	-0.2 (-0.2, -0.1)	<0.0001	-0.2 (-0.2, -0.1)	<0.0001
Public health facility setting (versus other settings)	6.7 (2.5, 10.8)	0.002	6.0 (1.8, 10.3)	0.01	6.7 (2.5, 10.8)	0.002
Country was in Asia	-5.5 (-9.4, -1.5)	0.01	-5.4 (-9.3, -1.5)	0.01	-5.5 (-9.4, -1.5)	0.01
Study data were complete	NA	NA	1.8 (-2.5, 6.2)	0.40	NA	NA
Performance outcome was easy to interpret	NA	NA	3.3 (-0.1, 6.6)	0.06	NA	NA
Natural log of clusters involved	NA	NA	-1.5 (-3.0, -0.04)	0.04	NA	NA

Footnotes.

Abbreviation: CI = Confidence interval, NA = not applicable.

[a] Based on score statistics for type 3 tests of fixed effects, which tends to give conservative estimates. The one exception is the p-value of the intercept, which was based on the t test, which tends to give less conservative estimates. This is the only p-value provided in the SAS output for the intercept. Note that the conclusion of the test (significant or not, based on a 0.05 cutoff) from the two sets of p-values (t test vs. type 3 test) always agreed.

[b] Dichotomous variable with a value of one if the strategy included a component from a given strategy component category (e.g., low-intensity training), otherwise the variable has a value of zero. The parameter estimate is the mean effect of the strategy component category, adjusted for other components in the strategy and other factors in the model.

Note: The adjusted R-square of the model without any contextual or methodological factors was 0.0550. The adjusted R-square of Model 1, Model 2, and Model 3, was 0.2151, 0.2381, and 0.2151, respectively.

Table H2. Model to estimate strategy component effectiveness and identify contextual and methodological factors associated with effect size for practice outcome expressed as a percentage (including both professional health care providers and lay health workers): All training combined into one category

Factor	Model 1, to identify contextual factors to adjust effect sizes		Model 2, to identify predictors of effect sizes		Model 3, to estimate the marginal effect of each strategy component category	
	Parameter estimate (95% CI)	P-value[a]	Parameter estimate (95% CI)	P-value[a]	Parameter estimate (95% CI)	P-value[a]
Intercept	7.6 (2.1, 13.1)	0.01	4.3 (-3.7, 12.3)	0.29	5.1 (-1.1, 11.2)	0.11
Dummy variables[b] that each code for a strategy component category						
Community support	0.3 (-5.0, 5.6)	0.92	-0.5 (-6.4, 5.3)	0.86	0.4 (-5, 5.7)	0.90
Patient support	-3.6 (-9.5, 2.2)	0.22	-4.3 (-10.6, 2.1)	0.19	-2.7 (-8.7, 3.3)	0.38
Strengthening infrastructure	-1.2 (-7.2, 4.9)	0.71	-1.5 (-8.3, 5.2)	0.66	-1.5 (-7.6, 4.7)	0.64
HCP-directed financial incentives	6.1 (-6.5, 18.6)	0.34	6.1 (-6.4, 18.6)	0.34	6.9 (-5.5, 19.4)	0.27
Health system financing and other incentives	2.5 (-3.4, 8.5)	0.40	2.0 (-4.5, 8.6)	0.55	2.6 (-3.6, 8.8)	0.41
Regulation and governance	2.7 (-4.3, 9.7)	0.45	2.6 (-4.4, 9.5)	0.47	2.5 (-4.6, 9.6)	0.50
Group problem solving	13.6 (5.7, 21.6)	0.001	12.7 (4.5, 20.8)	0.003	14.5 (6.4, 22.6)	0.001
Supervision	1.0 (-1.8, 3.9)	0.48	0.3 (-3.4, 4.0)	0.88	1.5 (-1.3, 4.3)	0.30
Other management techniques	3.3 (-1.8, 8.5)	0.21	2.8 (-3.0, 8.6)	0.34	2.9 (-2.4, 8.1)	0.28
Any training	6.4 (1.5, 11.2)	0.01	5.5 (-0.2, 11.2)	0.06	6.1 (1.1, 11.2)	0.02
Printed information or job aid for HCPs	-1 (-6.6, 4.6)	0.72	-1.7 (-8.1, 4.7)	0.61	-1.4 (-7.1, 4.2)	0.61
Information and communication technology for HCPs	-2.4 (-8.6, 3.8)	0.44	-3.3 (-10.1, 3.4)	0.33	-2.1 (-8.5, 4.2)	0.51
Contextual and methodological factors (all mean-centered)						
Baseline performance level	-0.2 (-0.2, -0.1)	<0.0001	-0.2 (-0.2, -0.1)	<0.0001	-0.2 (-0.2, -0.1)	<0.0001
Public health facility setting (versus other settings)	6.8 (2.6, 10.9)	0.002	7.3 (3.1, 11.6)	0.001	5.6 (1.5, 9.8)	0.01
Country was in Asia	-5.3 (-9.2, -1.4)	0.01	-5.2 (-9.1, -1.3)	0.01	NA	NA
Study data were complete	NA	NA	2.8 (-1.7, 7.3)	0.21	NA	NA
Performance outcome was easy to interpret	NA	NA	3.1 (-0.3, 6.4)	0.07	NA	NA
Number of components of strategy involved in comparison	NA	NA	0.3 (-1.1, 1.8)	0.65	NA	NA
Data collected through record review	NA	NA	NA	NA	2.1 (-1.9, 6.2)	0.30

Footnotes.

Abbreviation: CI = Confidence interval, NA = not applicable.

[a] Based on score statistics for type 3 tests of fixed effects, which tends to give conservative estimates. The one exception is the p-value of the intercept, which was based on the t test, which tends to give less conservative estimates. This is the only p-value provided in the SAS output for the intercept. Note that the conclusion of the test (significant or not, based on a 0.05 cutoff) from the two sets of p-values (t test vs. type 3 test) always agreed.

[b] Dichotomous variable with a value of one if the strategy included a component from a given strategy component category (e.g., training), otherwise the variable has a value of zero. The parameter estimate is the mean effect of the strategy component category, adjusted for other components in the strategy and other factors in the model.

Note: The adjusted R-square of the model without any contextual or methodological factors was 0.05567. The adjusted R-square of Model 1, Model 2, and Model 3, was 0.2155, 0.2345, and 0.2081, respectively.

Table I. The effectiveness of strategies to improve health care provider performance for studies of professional health care providers with at least one practice outcome with low- and high-intensity training analyzed separately

Strategy	Practice outcomes expressed as a percentage				Continuous practice outcomes		
	No. of study comparisons (no. with low or moderate risk of bias)	No. of countries studied	Median MES based on adjusted effect sizes[a] (interquartile range; minimum, maximum)		No. of study comparisons (no. with low or moderate risk of bias)	No. of countries studied	Median MES based on unadjusted effect sizes (interquartile range; minimum, maximum)
Strategies tested by at least three comparisons with percentage outcomes or at least three comparisons with continuous outcomes (descending order of effect size for percentage outcomes)							
Strengthening infrastructure + health system financing and other incentives + supervision + other management techniques + low-intensity training	3 (1)	2	57.5 (NA, NA; 4.3, 58.5)		0 (0)	0	NA
Group problem solving + low-intensity training	4 (1)	2	55.9 (40.8, 68.5; 29.2, 77.8)		1 (1)	1	52.4
Strengthening infrastructure + supervision + other management techniques + low-intensity training	1 (1)	1	29.4		4 (1)	4	183.2 (NA, NA; 56.9, 615.5)
Group problem solving only	12 (3)	10	27.9 (12.0, 41.7; 5.5, 61.2)		4 (0)	3	-8.1 (-24.3, 44.2; -28.2, 84.1)
Community support + supervision + low-intensity training	4 (2)	4	20.6 (7.4, 24.2; -3.0, 25.1)		0 (0)	0	NA
Other management techniques + printed information or job aid for HCPs	2 (2)	2	18.3 (NA, NA; 4.6, 31.9)		3 (3)	3	11.8 (NA, NA; 0.3,16.5)
Supervision + high-intensity training	9 (5)	7	18.9 (3.0, 26.1; -2.0, 30.8)		3 (2)	2	7.3 (NA, NA; -16.3,101.1)
Other management techniques only	4 (3)	3	16.5 (2.2, 21.4; -11.2, 25.3)		0 (0)	0	NA

Strategy	Practice outcomes expressed as a percentage				Continuous practice outcomes		
	No. of study comparisons (no. with low or moderate risk of bias)	No. of countries studied	Median MES based on adjusted effect sizes[a] (interquartile range; minimum, maximum)		No. of study comparisons (no. with low or moderate risk of bias)	No. of countries studied	Median MES based on unadjusted effect sizes (interquartile range; minimum, maximum)
Other management techniques + low-intensity training	3 (0)	3	15.9 (NA, NA; -1.7, 24.0)		1 (0)	1	8.3
Supervision only	16 (8)	12	14.8 (6.4, 25.2; -6.1, 56.2)		3 (1)	3	-3.0 (NA, NA; -90.4, 31.4)
Supervision + low-intensity training	17 (6)	13	13.8 (6.1, 23.9; -2.7, 67.0)		5 (1)	3	11.1 (10.5, 20.4; -2.2, 60.4)
Strengthening infrastructure only	3 (3)	3	13.0 (NA, NA; -7.0, 15.8)		2 (2)	2	152.1 (NA, NA; 4.2, 300.0)
Patient support + low-intensity training	6 (3)	6	11.1 (2.4, 15.3; -6.5, 31.5)		1(0)	1	73.3
Low-intensity training only	56 (23)	28	10.4 (5.0, 23.5; -20.0, 60.8)		14 (7)	9	17.5 (-4.6, 23.7; -25.0, 81.4)
High-intensity training only	22 (10)	14	9.1 (6.3, 14.5; -3.9, 37.2)		2 (1)	1	25.3 (NA, NA; 17.4, 33.3)
Supervision + other management techniques	4 (0)	3	7.6 (-1.4, 11.6; -7.9, 13.1)		2 (0)	2	94.3 (NA, NA; -9.2, 197.9)
Group problem solving + information and communication technology for HCPs	3 (3)	3	6.5 (NA, NA; -3.6, 32.4)		0 (0)	0	NA
Supervision + printed information or job aid for HCPs	3 (3)	7	2.3 (NA, NA; 2.1, 24.3)		3 (1)	3	-3.7 (NA, NA; -7.1, 16.7)
Health system financing and other incentives only	2 (0)	2	1.4 (NA, NA; -2.4, 5.2)		3 (2)	2	20.4 (NA, NA; -23.9,72.4)

Strategy	Practice outcomes expressed as a percentage			Continuous practice outcomes		
	No. of study comparisons (no. with low or moderate risk of bias)	No. of countries studied	Median MES based on adjusted effect sizes[a] (interquartile range; minimum, maximum)	No. of study comparisons (no. with low or moderate risk of bias)	No. of countries studied	Median MES based on unadjusted effect sizes (interquartile range; minimum, maximum)
Strengthening infrastructure + supervision + low-intensity training + information and communication technology for HCPs	3 (2)	3	1.3 (NA, NA; -1.5, 20.1)	0 (0)	0	NA
Printed information or job aid for HCPs only	8 (5)	7	1.3 (-4.9, 6.1; -13.8, 11.8)	3(1)	2	-3.4 (NA, NA;-72.0, 6.5)
Supervision + other management techniques + high-intensity training	3 (1)	2	0.9 (NA, NA; -6.6, 11.4)	0 (0)	0	NA
Information and communication technology for HCPs only	4 (4)	3	0.8 (-3.0, 9.8; -3.0, 15.0)	1 (1)	1	-38.9
Strengthening infrastructure + supervision + low-intensity training	2 (1)	2	-0.9 (NA, NA; -4.8, 3.1)	4 (4)	3	64.3 (31.9, 88.7; 2.6, 110.1)
Strategies tested by less than three comparisons with percentage outcomes and less than three comparisons with continuous outcomes (descending order of effect size for percentage outcomes[b])						
Community support + HCP-directed financial incentives + health system financing and other incentives + group problem solving + other management techniques	0 (0)	0	NA	1 (0)	1	375.2
Community support + strengthening infrastructure + supervision + other management techniques + high-intensity training	0 (0)	0	NA	1 (0)	1	153.0

Strategy	Practice outcomes expressed as a percentage			Continuous practice outcomes		
	No. of study comparisons (no. with low or moderate risk of bias)	No. of countries studied	Median MES based on adjusted effect sizes[a] (interquartile range; minimum, maximum)	No. of study comparisons (no. with low or moderate risk of bias)	No. of countries studied	Median MES based on unadjusted effect sizes (interquartile range; minimum, maximum)
Community support + strengthening infrastructure + health system financing and other incentives + regulation and governance + supervision + other management techniques + low-intensity training	0 (0)	0	NA	1 (0)	1	121.3
Health system financing and other incentives + other management techniques	0 (0)	0	NA	1 (0)	1	83.0
Strengthening infrastructure + group problem solving	1 (1)	1	72.3	0 (0)	0	NA
Community support + strengthening infrastructure + health system financing and other incentives + regulation and governance + supervision + low-intensity training	0 (0)	0	NA	1 (0)	1	70.0
Community support + strengthening infrastructure + HCP-directed financial incentives + health system financing and other incentives + regulation and governance + supervision+ other management techniques + low-intensity [illegible]	1 (0)	1	60.5	0 (0)	0	NA
Supervision + low-intensity training + information and communication technology for HCPs	1 (0)	1	59.4	0 (0)	0	NA

Strategy	Practice outcomes expressed as a percentage				Continuous practice outcomes		
	No. of study comparisons (no. with low or moderate risk of bias)	No. of countries studied	Median MES based on adjusted effect sizes[a] (interquartile range; minimum, maximum)		No. of study comparisons (no. with low or moderate risk of bias)	No. of countries studied	Median MES based on unadjusted effect sizes (interquartile range; minimum, maximum)
Group problem solving + supervision + high-intensity training	0 (0)	0	NA		1 (1)	1	58.1
Patient support + other management techniques + high-intensity training	0 (0)	0	NA		1 (0)	1	57.8
Community support + HCP-directed financial incentives + other management techniques + low-intensity training + information and communication technology for HCPs	1 (1)	1	56.1		0 (0)	0	NA
Community support + regulation and governance + low-intensity training	1 (0)	1	40.1		0 (0)	0	NA
Strengthening infrastructure + supervision + high-intensity training	2 (0)	2	39.8<br>(NA, NA;14.7, 64.9)		0 (0)	0	NA
Health system financing and other incentives + supervision + high-intensity training	1 (0)	1	39.3		0 (0)	0	NA
Community support + regulation and governance + supervision	0 (0)	0	NA		1 (0)	1	38.1
Strengthening infrastructure + supervision + other management techniques + high-intensity training	1 (1)	1	36.9		0 (0)	0	NA

Strategy	Practice outcomes expressed as a percentage				Continuous practice outcomes		
	No. of study comparisons (no. with low or moderate risk of bias)	No. of countries studied	Median MES based on adjusted effect sizes[a] (interquartile range; minimum, maximum)		No. of study comparisons (no. with low or moderate risk of bias)	No. of countries studied	Median MES based on unadjusted effect sizes (interquartile range; minimum, maximum)
Group problem solving + supervision + other management techniques + high-intensity training + information and communication technology for HCPs	1 (1)	2	34.9		0 (0)	0	NA
Community support + regulation and governance + supervision + other management techniques + low-intensity training	1 (0)	1	34.4		0 (0)	0	NA
Strengthening infrastructure + health system financing and other incentives + group problem solving + supervision + other management techniques + low-intensity training	1 (0)	1	32.9		0 (0)	0	NA
Community support + strengthening infrastructure + health system financing and other incentives + supervision + other management techniques + high-intensity training	1 (0)	1	32.4		0 (0)	0	NA
Community support + patient support + strengthening infrastructure + regulation and governance + supervision + other management techniques + low-intensity training	2 (1)	1	32.1 (NA, NA; 27.5, 36.7)		0 (0)	0	NA

Strategy	Practice outcomes expressed as a percentage				Continuous practice outcomes		
	No. of study comparisons (no. with low or moderate risk of bias)	No. of countries studied	Median MES based on adjusted effect sizes[a] (interquartile range; minimum, maximum)		No. of study comparisons (no. with low or moderate risk of bias)	No. of countries studied	Median MES based on unadjusted effect sizes (interquartile range; minimum, maximum)
Health system financing and other incentives + high-intensity training	1 (0)	1	30.0		0 (0)	0	NA
Patient support + other management techniques + printed information or job aid for HCPs	1 (0)	1	29.2		0 (0)	0	NA
Patient support + strengthening infrastructure + supervision + other management technique + low-intensity training	1 (1)	1	29.0		0 (0)	0	NA
Health system financing and other incentives+ regulation and governance + supervision + other management techniques	1 (0)	1	28.9		0 (0)	0	NA
Other management techniques + high-intensity training	2 (1)	2	28.4<br>(NA, NA; 2.8, 54.0)		1 (0)	1	9.9
Community support + strengthening infrastructure + health system financing and other incentives + regulation and governance + high-intensity training	1 (1)	1	27.1		0 (0)	0	NA
Community support + strengthening infrastructure + supervision + other management techniques + low-intensity training	1 (0)	1	26.8		2 (1)	2	75.0

Strategy	Practice outcomes expressed as a percentage				Continuous practice outcomes		
	No. of study comparisons (no. with low or moderate risk of bias)	No. of countries studied	Median MES based on adjusted effect sizes[a] (interquartile range; minimum, maximum)		No. of study comparisons (no. with low or moderate risk of bias)	No. of countries studied	Median MES based on unadjusted effect sizes (interquartile range; minimum, maximum)
Community support + strengthening infrastructure + health system financing and other incentives + supervision + low-intensity training	2 (1)	2	26.2 (NA, NA; 14.0, 38.3)		0 (0)	0	NA
HCP-directed financial incentives only	2 (1)	2	25.9 (NA, NA; 11.1, 40.7)		1 (1)	1	66.7
Low-intensity training + information and communication technology for HCPs	2 (0)	2	25.8 (NA, NA; 8.2,43.3)		0 (0)	0	NA
Health system financing and other incentives + regulation and governance + supervision	1 (0)	1	24.6		0 (0)	0	NA
Group problem solving + other management techniques	2 (0)	2	24.3 (NA, NA; 9.2, 39.3)		0 (0)	0	NA
Strengthening infrastructure + other management techniques + printed information or job aid for HCPs	1 (1)	1	23.1		0 (0)	0	NA
Community support + strengthening infrastructure + health system financing and other incentives + high-intensity training	1 (0)	1	21.1		0 (0)	0	NA

Strategy	Practice outcomes expressed as a percentage				Continuous practice outcomes		
	No. of study comparisons (no. with low or moderate risk of bias)	No. of countries studied	Median MES based on adjusted effect sizes[a] (interquartile range; minimum, maximum)		No. of study comparisons (no. with low or moderate risk of bias)	No. of countries studied	Median MES based on unadjusted effect sizes (interquartile range; minimum, maximum)
Community support + strengthening infrastructure + health system financing and other incentives + regulation and governance + supervision + high-intensity training	0 (0)	0	NA		1 (1)	1	20.3
Patient support + HCP-directed financial incentives + regulation and governance + supervision + other management techniques + low-intensity training	0 (0)	0	NA		1 (0)	1	18.9
Community support + low-intensity training	2 (0)	2	18.8 (NA, NA; 8.0, 29.6)		1 (1)	1	4.5
Patient support + supervision + other management techniques + low-intensity training	2 (1)	2	17.7 (NA, NA; 3.2, 32.1)		0 (0)	0	NA
Strengthening infrastructure + high-intensity training	2 (1)	2	17.5 (NA, NA; 2.0, 33.0)		0 (0)	0	NA
Patient support + strengthening infrastructure + regulation and governance + supervision + other management techniques + low-intensity training	1 (0)	1	17.1		0 (0)	0	NA
Supervision + information and communication technology for HCPs	2 (2)	1	16.8 (NA, NA; 13.8, 19.7)		1 (1)	1	36.4
Community support + other management techniques	1 (1)	1	15.9		1 (1)	1	-6.0

Strategy	Practice outcomes expressed as a percentage				Continuous practice outcomes		
	No. of study comparisons (no. with low or moderate risk of bias)	No. of countries studied	Median MES based on adjusted effect sizes[a] (interquartile range; minimum, maximum)		No. of study comparisons (no. with low or moderate risk of bias)	No. of countries studied	Median MES based on unadjusted effect sizes (interquartile range; minimum, maximum)
Community support + strengthening infrastructure + health system financing and other incentives + supervision + high-intensity training + information and communication technology for HCPs	1 (1)	1	15.8		0 (0)	0	NA
Patient support + printed information or job aid for HCPs	2 (0)	2	15.3 (NA, NA; 2.3, 28.3)		0 (0)	0	NA
Community support + patient support + other management techniques + printed information or job aid for HCPs	1 (0)	1	15.2		0 (0)	0	NA
Community support + high-intensity training	2 (0)	2	15.1 (NA, NA; 9.8, 20.4)		0 (0)	0	NA
Strengthening infrastructure + group problem solving + supervision + low-intensity training	2 (0)	1	14.8 (NA, NA; 12.4, 17.2)		0 (0)	0	NA
Community support + patient support + strengthening infrastructure + group problem solving + other management techniques + low-intensity training	1 (1)	1	14.7		0 (0)	0	NA

Strategy	Practice outcomes expressed as a percentage				Continuous practice outcomes		
	No. of study comparisons (no. with low or moderate risk of bias)	No. of countries studied	Median MES based on adjusted effect sizes[a] (interquartile range; minimum, maximum)		No. of study comparisons (no. with low or moderate risk of bias)	No. of countries studied	Median MES based on unadjusted effect sizes (interquartile range; minimum, maximum)
Patient support + health system financing and other incentives + group problem solving + supervision + other management techniques + high-intensity training	1 (1)	1	14.2		0 (0)	0	NA
Community support + strengthening infrastructure + low-intensity training	2 (0)	2	14.0 (NA, NA; 5.4, 22.6)		0 (0)	0	NA
Regulation and governance + supervision + other management techniques + low-intensity training	2 (1)	2	13.3 (NA, NA; 2.9, 23.8)		0 (0)	0	NA
Strengthening infrastructure + Health system financing and other incentives + supervision + high-intensity training	1 (0)	2[c]	13.2		0 (0)	0	NA
Health system financing and other incentives + information and communication technology for HCPs	2 (0)	2	12.9 (NA, NA; 11.4, 14.5)		2 (0)	2	-2 (NA, NA; -0.4, -0.1)
Strengthening infrastructure + health system financing and other incentives + other management techniques + printed information or job aid for HCPs	2 (1)	1	12.4 (NA, NA; 1.9, 23.0)		2 (1)	1	-0.9 (NA, NA; -2.2, 0.4)

Strategy	Practice outcomes expressed as a percentage				Continuous practice outcomes		
	No. of study comparisons (no. with low or moderate risk of bias)	No. of countries studied	Median MES based on adjusted effect sizes[a] (interquartile range; minimum, maximum)		No. of study comparisons (no. with low or moderate risk of bias)	No. of countries studied	Median MES based on unadjusted effect sizes (interquartile range; minimum, maximum)
Strengthening infrastructure + health system financing and other incentives	0 (0)	0	NA		1 (0)	1	12.3
Strengthening infrastructure + health system financing and other incentives + low-intensity training	2 (2)	1	12.3 (NA, NA; 5.8, 18.8)		0 (0)	0	NA
Strengthening infrastructure + supervision + printed information or job aid for HCPs + information and communication technology for HCPs	1 (1)	1	12.1		0 (0)	0	NA
Supervision + low-intensity training + printed information or job aid for HCPs	1 (0)	1	12.1		0 (0)	0	NA
Regulation and governance + supervision + high-intensity training	1 (0)	1	11.8		0 (0)	0	NA
Strengthening infrastructure + other management techniques + printed information or job aid for HCPs + information and communication technology for HCPs	1 (1)	1	11.7		0 (0)	0	NA

Strategy	Practice outcomes expressed as a percentage				Continuous practice outcomes		
	No. of study comparisons (no. with low or moderate risk of bias)	No. of countries studied	Median MES based on adjusted effect sizes[a] (interquartile range; minimum, maximum)		No. of study comparisons (no. with low or moderate risk of bias)	No. of countries studied	Median MES based on unadjusted effect sizes (interquartile range; minimum, maximum)
Strengthening infrastructure + HCP-directed financial incentives + health system financing and other incentives + regulation and governance	1 (0)	1	11.6		1 (0)	1	-4.2
Health system financing and other incentives + low-intensity training	1 (0)	1	11.4		1 (0)	1	34.8
Patient support + strengthening infrastructure + health system financing and other incentives + supervision + other management techniques + low-intensity training	1 (0)	1	10.8		1 (0)	1	-7.4
Community support + strengthening infrastructure+ group problem solving + supervision + other management techniques + low-intensity training	1 (0)	1	10.2		0 (0)	0	NA
Group problem solving + supervision + printed information or job aid for HCPs	2 (2)	2	10.1<br>(NA, NA; 6.3, 13.8)		1 (1)	1	-6.5
Community support + strengthening infrastructure + other management techniques + low-intensity training	1 (0)	1	9.8		1 (0)	1	30.6

Strategy	Practice outcomes expressed as a percentage				Continuous practice outcomes		
	No. of study comparisons (no. with low or moderate risk of bias)	No. of countries studied	Median MES based on adjusted effect sizes[a] (interquartile range; minimum, maximum)		No. of study comparisons (no. with low or moderate risk of bias)	No. of countries studied	Median MES based on unadjusted effect sizes (interquartile range; minimum, maximum)
Patient support + strengthening infrastructure + supervision + low-intensity training	1 (0)	1	9.8		0 (0)	0	NA
Strengthening infrastructure + health system financing and other incentives + printed information or job aid for HCPs	1 (1)	1	9.5		1 (1)	1	-25.4
Strengthening infrastructure + health system financing and other incentives + regulation and governance	1 (0)	1	9.1		1 (0)	1	5.9
Group problem solving + printed information or job aid for HCPs	1 (1)	1	8.8		0 (0)	0	NA
Group problem solving + supervision + low-intensity training	2 (1)	2	8.7<br>(NA, NA; 1.4, 16.1)		1 (0)	1	7.3
Regulation and governance + supervision + other management techniques + low-intensity training + information and communication technology for HCPs	1 (1)	1	8.4		0 (0)	0	NA
Patient support + strengthening infrastructure + health system financing and other incentives + supervision + low-intensity training	1 (0)	1	7.8		0 (0)	0	NA

Strategy	Practice outcomes expressed as a percentage				Continuous practice outcomes		
	No. of study comparisons (no. with low or moderate risk of bias)	No. of countries studied	Median MES based on adjusted effect sizes[a] (interquartile range; minimum, maximum)		No. of study comparisons (no. with low or moderate risk of bias)	No. of countries studied	Median MES based on unadjusted effect sizes (interquartile range; minimum, maximum)
HCP-directed financial incentives + health system financing and other incentives + supervision + high-intensity training	1 (0)	1	7.2		0 (0)	0	NA
Community support + strengthening infrastructure + health system financing and other incentives + regulation and governance + supervision + other management techniques + high-intensity training	1 (0)	1	7.2		0 (0)	0	NA
Strengthening infrastructure + supervision	2 (1)	2	6.9 (NA, NA; -8.5, 22.3)		2 (1)	2	15.8 (NA, NA; -16.4, 47.9)
Community support + patient support + strengthening infrastructure + HCP-directed financial incentives + supervision + high-intensity training	1 (1)	4[c]	6.8		1 (1)	4[c]	0.0
Health system financing and other incentives + supervision	1 (0)	1	6.7		1 (0)	1	56.5
HCP-directed financial incentives + information and communication technology for HCPs	1 (0)	1	6.4		0 (0)	0	NA

Strategy	Practice outcomes expressed as a percentage				Continuous practice outcomes		
	No. of study comparisons (no. with low or moderate risk of bias)	No. of countries studied	Median MES based on adjusted effect sizes[a] (interquartile range; minimum, maximum)		No. of study comparisons (no. with low or moderate risk of bias)	No. of countries studied	Median MES based on unadjusted effect sizes (interquartile range; minimum, maximum)
Community support + strengthening infrastructure + HCP-directed financial incentives + health system financing and other incentives + supervision + other management techniques + low-intensity training	1 (0)	1	6.4		0 (0)	0	NA
Patient support + supervision + low-intensity training	2 (1)	2	6.3<br>(NA, NA; 5.2, 7.5)		1 (0)	1	14.2
Strengthening infrastructure + regulation and governance + low-intensity training + information and communication technology for HCPs	1 (1)	1	6.1		0 (0)	0	NA
HCP-directed financial incentives + health system financing and other incentives	0 (0)	0	NA		1 (0)	1	5.9
Group problem solving + supervision + other management techniques + low-intensity training	1 (0)	1	5.6		0 (0)	0	NA
Community support + strengthening infrastructure + health system financing and other incentives	1 (0)	1	5.6		2 (0)	2	0.4<br>(NA, NA; -1.7, 2.5)
Supervision + other management techniques + low-intensity training	2 (1)	2	5.3<br>(NA, NA; -16.2, 26.9)		2 (2)	2	30.1<br>(NA, NA; 28.3,31.9)

Strategy	Practice outcomes expressed as a percentage				Continuous practice outcomes		
	No. of study comparisons (no. with low or moderate risk of bias)	No. of countries studied	Median MES based on adjusted effect sizes[a] (interquartile range; minimum, maximum)		No. of study comparisons (no. with low or moderate risk of bias)	No. of countries studied	Median MES based on unadjusted effect sizes (interquartile range; minimum, maximum)
HCP-directed financial incentives + health system financing and other incentives + information and communication technology for HCPs	1 (0)	1	5.0		0 (0)	0	NA
Regulation and governance + supervision + low-intensity training	1 (0)	1	4.8		0 (0)	0	NA
Patient support + other management techniques	2 (1)	2	4.7 (NA, NA; -2.3, 11.6)		1 (1)	1	-4.3
Community support + regulation and governance + other management techniques	1	1	3.8		0 (0)	0	NA
Community support + HCP-directed financial incentives + health system financing and other incentives + regulation and governance + group problem solving + other management techniques	0 (0)	0	NA		1 (0)	1	3.5
Printed information or job aid for HCPs + information and communication technology for HCPs	2 (1)	2	3.4 (NA, NA; -3.3, 10.1)		0 (0)	0	NA

Strategy	Practice outcomes expressed as a percentage				Continuous practice outcomes		
	No. of study comparisons (no. with low or moderate risk of bias)	No. of countries studied	Median MES based on adjusted effect sizes[a] (interquartile range; minimum, maximum)		No. of study comparisons (no. with low or moderate risk of bias)	No. of countries studied	Median MES based on unadjusted effect sizes (interquartile range; minimum, maximum)
Patient support + strengthening infrastructure + regulation and governance + other management techniques + printed information or job aid for HCPs	1 (0)	1	2.7		0 (0)	0	NA
Regulation and governance + group problem solving	1 (1)	1	2.7		0 (0)	0	NA
Community support + health system financing and other incentives + regulation and governance	1 (0)	1	1.1		0 (0)	0	NA
Community support + strengthening infrastructure + regulation and governance + supervision	1 (1)	1	-0.7		0 (0)	0	NA
Strengthening infrastructure + group problem solving + supervision + other management techniques	1 (0)	1	-2.9		1 (0)	1	-2.2
Community support + strengthening infrastructure	2 (0)	1	-3.5<br>(NA, NA; -8.4, 1.4)		1 (0)	1	2.1
Community support + HCP-directed financial incentives	0 (0)	0	NA		1 (1)	1	-29.3

Footnotes.

GRADE = The Grading of Recommendations Assessment, Development, and Evaluation system, HCP = health care provider, MES = median effect size, NA = not applicable.

[a] Effect sizes expressed as an absolute percentage-point change. See methods section for details on adjustment.

[b] Unless no studies with percentage outcomes were found, in which case results of continuous outcomes were used.

[c] Multi-country study.

Table J1. The effectiveness of strategies to improve health care provider performance for studies of professional health care providers with at least one practice outcome: strategies tested by one or two study comparisons (descending order of effect size for percentage outcomes[a])

Strategy	Practice outcomes expressed as a percentage					Continuous practice outcomes			
	No. of study comparisons (no. with low or moderate risk of bias)	No. of countries studied	Median MES based on adjusted effect sizes[b] (interquartile range; minimum, maximum)	GRADE quality of evidence		No. of study comparisons (no. with low or moderate risk of bias)	No. of countries studied	Median MES based on unadjusted effect sizes (interquartile range; minimum, maximum)	GRADE quality of evidence
Community support + HCP-directed financial incentives + health system financing and other incentives + group problem solving + other management techniques	0 (0)	0	NA	NA		1 (0)	1	375.2	Very low
Health system financing and other incentives + other management techniques	0 (0)	0	NA	NA		1(0)	1	83.0	Low
Strengthening infrastructure + group problem solving	1 (1)	1	72.5	Moderate		0 (0)	0	NA	NA
Community support + strengthening infrastructure + HCP-directed financial incentives + health system financing and other incentives + regulation and governance + supervision + other management techniques + any training	1 (0)	1	60.6	Very low		0(0)	0	NA	NA
Supervision + any training + information and communication technology for HCPs	1 (0)	1	59.4	Low		0 (0)	0	NA	NA
Patient support + other management techniques + any training	0 (0)	0	NA	NA		1 (0)	1	57.8	Low

Strategy	Practice outcomes expressed as a percentage					Continuous practice outcomes			
	No. of study comparisons (no. with low or moderate risk of bias)	No. of countries studied	Median MES based on adjusted effect sizes[b] (interquartile range; minimum, maximum)	GRADE quality of evidence		No. of study comparisons (no. with low or moderate risk of bias)	No. of countries studied	Median MES based on unadjusted effect sizes (interquartile range; minimum, maximum)	GRADE quality of evidence
Community support + HCP-directed financial incentives + other management techniques + any training + information and communication technology for HCPs	1 (1)	1	56.1	High		0 (0)	0	NA	NA
Community support + strengthening infrastructure + health system financing and other incentive + regulation and governance + supervision + any training	0 (0)	0	NA	NA		2 (1)	2	45.1<br>(NA, NA; 20.3, 70.0)	Low
Community support + regulation and governance + any training	1 (0)	1	40.0	Low		0 (0)	0	NA	NA
Health system financing and other incentives + supervision + any training	1 (0)	1	39.4	Very low		0 (0)	0	NA	NA
Community support + regulation and governance + supervision	0 (0)	0	NA	NA		1 (0)	1	38.1	Very low
Group problem solving + supervision + other management techniques + any training + information and communication technology for HCPs	1 (1)	2[b]	34.9	Moderate		0 (0)	0	NA	NA
Community support + regulation and governance + supervision + other management techniques + any training	1 (0)	1	34.5	Very low		0 (0)	0	NA	NA

Strategy	Practice outcomes expressed as a percentage					Continuous practice outcomes			
	No. of study comparisons (no. with low or moderate risk of bias)	No. of countries studied	Median MES based on adjusted effect sizes[b] (interquartile range; minimum, maximum)	GRADE quality of evidence		No. of study comparisons (no. with low or moderate risk of bias)	No. of countries studied	Median MES based on unadjusted effect sizes (interquartile range; minimum, maximum)	GRADE quality of evidence
Strengthening infrastructure + health system financing and other incentives + group problem solving + supervision + other management techniques + any training	1(0)	1	32.8	Very low		0 (0)	0	NA	NA
Community support + strengthening infrastructure + health system financing and other incentives + supervision + other management techniques + any training	1 (0)	1	32.6	Very low		0 (0)	0	NA	NA
Community support + patient support + strengthening infrastructure + regulation and governance + supervision + other management techniques + any training	2 (1)	1	32.2<br>(NA, NA; 27.6, 36.9)	Low		0 (0)	0	NA	NA
Patient support + other management techniques + printed information or job aid for HCPs	1 (0)	1	29.4	Very low		0 (0)	0	NA	NA
Patient support + strengthening infrastructure + supervision + other management techniques + any training	1 (1)	1	29.0	Moderate		0 (0)	0	NA	NA
Health system financing and other incentives + regulation and governance + supervision + other management techniques	1 (0)	1	28.8	Very low		0 (0)	0	NA	NA

Strategy	Practice outcomes expressed as a percentage					Continuous practice outcomes			
	No. of study comparisons (no. with low or moderate risk of bias)	No. of countries studied	Median MES based on adjusted effect sizes[b] (interquartile range; minimum, maximum)	GRADE quality of evidence		No. of study comparisons (no. with low or moderate risk of bias)	No. of countries studied	Median MES based on unadjusted effect sizes (interquartile range; minimum, maximum)	GRADE quality of evidence
Community support + strengthening infrastructure + health system financing and other incentives + regulation and governance + any training	1 (1)	1	27.2	Moderate		0 (0)	0	NA	NA
Community support + strengthening infrastructure + supervision + other management techniques + any training	1(0)	1	26.9	Very low		0 (0)	0	NA	NA
Community support + strengthening infrastructure + health system financing and other incentives + supervision + any training	2 (1)	2	26.2<br>(NA, NA; 14.2, 38.3)	Low		0 (0)	0	NA	NA
HCP-directed financial incentives only	2 (1)	2	26.0<br>(NA, NA; 11.2, 40.8)	Low		1 (1)	1	66.7	High
Any training + information and communication technology for HCPs	2 (0)	2	25.7<br>(NA, NA; 8.1, 43.3)	Very low		0 (0)	0	NA	NA
Health system financing and other incentives + regulation and governance + supervision	1 (0)	1	24.6	Very low		0 (0)	0	NA	NA
Group problem solving + other management techniques	2 (0)	2	24.4<br>(NA, NA; 9.4, 39.4)	Very low		0 (0)	0	NA	NA
Strengthening infrastructure + other management techniques + printed information or job aid for HCPs	1 (1)	1	23.2	Low		0 (0)	0	NA	NA

Strategy	Practice outcomes expressed as a percentage					Continuous practice outcomes			
	No. of study comparisons (no. with low or moderate risk of bias)	No. of countries studied	Median MES based on adjusted effect sizes[b] (interquartile range; minimum, maximum)	GRADE quality of evidence		No. of study comparisons (no. with low or moderate risk of bias)	No. of countries studied	Median MES based on unadjusted effect sizes (interquartile range; minimum, maximum)	GRADE quality of evidence
Community support + strengthening infrastructure + health system financing and other incentives + any training	1 (0)	1	21.2	Very low		0 (0)	0	NA	NA
Health system financing and other incentives + any training	2 (0)	2	20.8 (NA, NA; 11.4, 30.2)	Very low		1 (0)	1	34.8	Very low
Patient support + HCP-directed financial incentives + regulation and governance + supervision + other management techniques + any training	0 (0)	0	NA	NA		1 (0)	1	18.9	Very low
Patient support + supervision + other management techniques + any training	2 (1)	2	17.7 (NA, NA; 3.3, 32.1)	Very low		0 (0)	0	NA	NA
Strengthening infrastructure + any trainings	2 (1)	2	17.7 (NA, NA; 2.2, 33.2)	Very low		0 (0)	0	NA	NA
Patient support + Strengthening infrastructure + regulation and governance + supervision + other management techniques + any training	1 (0)	1	17.3	Very low		0 (0)	0	NA	NA
Supervision + information and communication technology for HCPs	2 (2)	1	16.8 (NA, NA; 13.9, 19.8)	Low		1 (1)	1	36.4	Low

Strategy	Practice outcomes expressed as a percentage					Continuous practice outcomes			
	No. of study comparisons (no. with low or moderate risk of bias)	No. of countries studied	Median MES based on adjusted effect sizes[b] (interquartile range; minimum, maximum)	GRADE quality of evidence		No. of study comparisons (no. with low or moderate risk of bias)	No. of countries studied	Median MES based on unadjusted effect sizes (interquartile range; minimum, maximum)	GRADE quality of evidence
Community support + strengthening infrastructure + health system financing and other incentives + supervision + any training + information and communication technology for HCPs	1 (1)	1	16.0	Moderate		0 (0)	0	NA	NA
Community support + other management techniques	1 (1)	1	15.7	Low		1 (1)	1	-6.0	Low
Patient support + printed information or job aid for HCPs	2 (0)	2	15.4 (NA, NA; 2.2, 28.5)	Very low		0 (0)	0	NA	NA
Community support + patient support + other management techniques + printed information or job aid for HCPS	1 (0)	1	15.2	Very low		0 (0)	0	NA	NA
Strengthening infrastructure + group problem solving + supervision + any training	2 (0)	1	14.8 (NA, NA; 12.4, 17.3)	Very low		0 (0)	0	NA	NA
Community support + patient support + strengthening infrastructure + group problem solving + other management techniques + any training	1 (1)	1	14.7	Low		0 (0)	0	NA	NA
Patient support + health system financing and other incentives + group problem solving + supervision + other management techniques + any training	1 (1)	1	14.3	Moderate		0 (0)	0	NA	NA

Strategy	Practice outcomes expressed as a percentage					Continuous practice outcomes			
	No. of study comparisons (no. with low or moderate risk of bias)	No. of countries studied	Median MES based on adjusted effect sizes[b] (interquartile range; minimum, maximum)	GRADE quality of evidence		No. of study comparisons (no. with low or moderate risk of bias)	No. of countries studied	Median MES based on unadjusted effect sizes (interquartile range; minimum, maximum)	GRADE quality of evidence
Community support + strengthening infrastructure + any training	2 (0)	2	14.0<br>(NA, NA; 5.5, 22.6)	Very low		0 (0)	0	NA	NA
Strengthening infrastructure + health system financing and other incentives + supervision + any training	1(0)	1	13.4	Very low		0 (0)	0	NA	NA
Regulation and governance + supervision + other management techniques + any training	2 (1)	2	13.3<br>(NA, NA; 2.8, 23.7)	Very low		0 (0)	0	NA	NA
Health system financing and other incentives + information and communication technology for HCPs	2 (0)	2	13.1<br>(NA, NA; 11.5, 14.6)	Very low		2 (0)	2	-0.2<br>(NA, NA; -0.4, -0.1)	Very low
Strengthening infrastructure + health system financing and other incentives + any training	2 (2)	1	12.4<br>(NA, NA; 5.9, 18.9)	Low		0 (0)	0	NA	NA
Strengthening infrastructure + health system financing and other incentives	0 (0)	0	NA	NA		1 (0)	1	12.3	Very low
Strengthening infrastructure + health system financing and other incentives + other management techniques + printed information or job aid for HCPs	2 (1)	1	12.3<br>(NA, NA; 1.7, 22.8)	Very low		2 (1)	1	-0.9<br>(NA, NA; -2.2, 0.4)	Very low

Strategy	Practice outcomes expressed as a percentage					Continuous practice outcomes			
	No. of study comparisons (no. with low or moderate risk of bias)	No. of countries studied	Median MES based on adjusted effect sizes[b] (interquartile range; minimum, maximum)	GRADE quality of evidence		No. of study comparisons (no. with low or moderate risk of bias)	No. of countries studied	Median MES based on unadjusted effect sizes (interquartile range; minimum, maximum)	GRADE quality of evidence
Strengthening infrastructure + supervision + printed information or job aid for HCPS + information and communication technology or HCPs	1 (1)	1	12.2	Moderate		0 (0)	0	NA	NA
Supervision + any training + printed information or job aid for HCPs	1 (0)	1	11.9	Very low		0 (0)	0	NA	NA
Strengthening infrastructure + other management techniques + printed information or job aid for HCPs + information and communication technology for HCPs	1 (1)	1	11.5	Low		0 (0)	0	NA	NA
Strengthening infrastructure + HCP-directed financial incentives + health system financing and other incentives + regulation and governance	1 (0)	1	11.5	Very low		1 (0)	1	-4.2	Very low
Patient support + strengthening infrastructure + health system financing and other incentives + supervision + other management techniques + any training	1 (0)	1	10.7	Very low		1 (0)	1	-7.4	Very low
Community support + strengthening infrastructure + group problem solving + supervision + other management techniques + any training	1 (0)	1	10.2	Very low		0 (0)	0	NA	NA

Strategy	Practice outcomes expressed as a percentage					Continuous practice outcomes			
	No. of study comparisons (no. with low or moderate risk of bias)	No. of countries studied	Median MES based on adjusted effect sizes[b] (interquartile range; minimum, maximum)	GRADE quality of evidence		No. of study comparisons (no. with low or moderate risk of bias)	No. of countries studied	Median MES based on unadjusted effect sizes (interquartile range; minimum, maximum)	GRADE quality of evidence
Group problem solving + supervision + printed information or job aid for HCPs	2 (2)	2	9.9 (NA, NA; 6.2, 13.7)	Low		1 (1)	1	-6.5	Low
Community support + strengthening infrastructure + other management techniques + any training	1 (0)	1	9.7	Very low		1 (0)	1	30.6	Very low
Patient support + strengthening infrastructure + supervision + any training	1 (0)	1	9.6	Very low		0 (0)	0	NA	NA
Strengthening infrastructure + health system financing and other incentives + printed information or job aid of HCPs	1 (1)	1	9.3	Low		1 (1)	1	-25.4	Low
Strengthening infrastructure + health system financing and other incentives + regulation and governance	1 (0)	1	9.2	Very low		1 (0)	1	5.9	Very low
Group problem solving + supervision + any training	2 (1)	2	8.8 (NA, NA; 1.5, 16.1)	Very low		2 (1)	2	32.7 (NA, NA; 7.3, 58.1)	Very low
Group problem solving + printed information or job aid for HCPs	1 (1)	1	8.7	Moderate		0 (0)	0	NA	NA
Regulation and governance + supervision + any training	2 (0)	2	8.4 (NA, NA; 4.9, 11.9)	Very low		0 (0)	0	NA	NA

Strategy	Practice outcomes expressed as a percentage					Continuous practice outcomes			
	No. of study comparisons (no. with low or moderate risk of bias)	No. of countries studied	Median MES based on adjusted effect sizes[b] (interquartile range; minimum, maximum)	GRADE quality of evidence		No. of study comparisons (no. with low or moderate risk of bias)	No. of countries studied	Median MES based on unadjusted effect sizes (interquartile range; minimum, maximum)	GRADE quality of evidence
Regulation and governance + supervision + other management techniques + any training + information and communication technology for HCPs	1 (1)	1	8.4	Moderate		0 (0)	0	NA	NA
Patient support + strengthening infrastructure + health system financing and other incentives + supervision + any training	1 (0)	1	8.0	Very low		0 (0)	0	NA	NA
Community support + strengthening infrastructure + health system financing and other incentives + regulation and governance + supervision + other management techniques + any training	1 (0)	1	7.2	Very low		1 (0)	1	121.3	Very low
HCP-directed financial incentives + health system financing and other incentives + supervision + any training	1 (0)	1	7.2	Very low		0 (0)	0	NA	NA
Community support + patient support + strengthening infrastructure + HCP-directed financial incentives + supervision + any training	1 (1)	4[c]	7.0	Moderate		1 (1)	4[c]	0.0	Moderate
Strengthening infrastructure + supervision	2 (1)	2	6.9 (NA, NA; -8.5, 22.2)	Very low		2 (1)	2	15.8 (NA, NA; -16.4, 47.9)	Very low

Strategy	Practice outcomes expressed as a percentage					Continuous practice outcomes			
	No. of study comparisons (no. with low or moderate risk of bias)	No. of countries studied	Median MES based on adjusted effect sizes[b] (interquartile range; minimum, maximum)	GRADE quality of evidence		No. of study comparisons (no. with low or moderate risk of bias)	No. of countries studied	Median MES based on unadjusted effect sizes (interquartile range; minimum, maximum)	GRADE quality of evidence
Community support + strengthening infrastructure + HCP-directed financial incentives + health system financing and other incentives + supervision + other management techniques + any training	1 (0)	1	6.6	Very low		0 (0)	0	NA	NA
Health system financing and other incentives + supervision	1 (0)	1	6.6	Very low		1 (0)	1	56.5	Very low
HCP-directed financial incentives + information and communication technology for HCPs	1 (0)	1	6.6	Very low		0 (0)	0	NA	NA
Patient support + supervision + any training	2 (1)	2	6.4 (NA, NA; 5.2, 7.5)	Very low		1 (0)	1	14.2	Very low
Strengthening infrastructure + regulation and governance + any training + information and communication technology for HCPs	1 (1)	1	6.0	Low		0 (0)	0	NA	NA
HCP-directed financial incentives + health system financing and other incentives	0 (0)	0	NA	NA		1 (0)	1	5.9	Very low
Group problem solving + supervision + other management techniques + any training	1 (0)	1	5.6	Very low		0 (0)	0	NA	NA

Section 5 – Additional results

Strategy	Practice outcomes expressed as a percentage					Continuous practice outcomes			
	No. of study comparisons (no. with low or moderate risk of bias)	No. of countries studied	Median MES based on adjusted effect sizes[b] (interquartile range; minimum, maximum)	GRADE quality of evidence		No. of study comparisons (no. with low or moderate risk of bias)	No. of countries studied	Median MES based on unadjusted effect sizes (interquartile range; minimum, maximum)	GRADE quality of evidence
Community support + strengthening infrastructure + health system financing and other incentives	1 (0)	1	5.4	Very low		2 (0)	2	0.4 (NA, NA; -1.7, 2.5)	Very low
HCP-directed financial incentives + health system financing and other incentives + information and communication technology for HCPs	1 (0)	1	5.0	Very low		0 (0)	0	NA	NA
Patient support + other management techniques	2 (1)	2	4.7 (NA, NA; -2.1, 11.4)	Low		1 (1)	1	-4.3	Moderate
Community support + regulation and governance + other management techniques	1 (1)	1	3.9	Low		0 (0)	0	NA	NA
Community support + HCP-directed financial incentives + health system financing and other incentives + regulation and [illegible]	0 (0)	0	NA	NA		1 (0)	1	3.5	Very low
Printed information or job aid for HCPs+ information and communication technology for HCPs	2 (1)	2	3.5 (NA, NA; -3.2, 10.2)	Very low		0 (0)	0	NA	NA
Patient support + strengthening infrastructure + regulation and governance + other management techniques + printed information or job aid for HCPs	1 (0)	1	2.9	Very low		0 (0)	0	NA	NA
Regulation and governance + group problem solving	1 (1)	1	2.7	Low		0 (0)	0	NA	NA

Strategy	Practice outcomes expressed as a percentage					Continuous practice outcomes			
	No. of study comparisons (no. with low or moderate risk of bias)	No. of countries studied	Median MES based on adjusted effect sizes[b] (interquartile range; minimum, maximum)	GRADE quality of evidence		No. of study comparisons (no. with low or moderate risk of bias)	No. of countries studied	Median MES based on unadjusted effect sizes (interquartile range; minimum, maximum)	GRADE quality of evidence
Community support + health system financing and other incentives + regulation and governance	1 (0)	1	1.2	Very low		0 (0)	0	NA	NA
Community support + strengthening infrastructure + regulation and governance + supervision	1 (1)	1	-0.8	Moderate		0 (0)	0	NA	NA
Strengthening infrastructure + group problem solving + supervision + other management techniques	1 (0)	1	-3.1	Very low		1 (0)	1	-2.2	Very low
Community support + strengthening infrastructure	2 (0)	1	-3.5 (NA, NA; -8.4, 1.4)	Very low		1 (0)	1	2.1	Very low
Community support + HCP-directed financial incentives	0 (0)	0	NA	NA		1 (1)	1	-29.3	Moderate

Footnotes.

GRADE = The Grading of Recommendations Assessment, Development, and Evaluation system, HCP = health care provider, MES = median effect size, NA = not applicable.

[a] Unless no studies with percentage outcomes were found, in which case results of continuous outcomes were used.

[b] Effect sizes expressed as an absolute percentage-point change. See methods section for details on adjustment.

[c] Multi-country study.

Table J2. The effectiveness of strategies to improve health care provider performance for studies predominantly of lay or community health workers with at least one practice outcome: strategies tested by one or two study comparisons (descending order of effect size for percentage outcomes[a])

Strategy	Practice outcomes expressed as a percentage				Continuous practice outcomes			
	No. of study comparisons (no. with low or moderate risk of bias)	No. of countries studied	Median MES based on unadjusted effect sizes (minimum, maximum)	GRADE quality of evidence	No. of study comparisons (no. with low or moderate risk of bias)	No. of countries studied	Median MES based on unadjusted effect sizes (minimum, maximum)	GRADE quality of evidence
Community support + patient support + HCP-directed financial incentives + supervision + any training + information and communication technology for HCPs	0	0	NA	NA	1 (0)	1	113.7	Low
Supervision + information and communication technology for HCPs	0	0	NA	NA	1 (1)	1	99.0	Moderate
Community support + any training	1 (0)	1	56.2	Low	0	0	NA	NA
Supervision + other management techniques + any training	1 (0)	1	50.0	Low	1(0)	1	58.1	Low
Information and communication technology for HCPs only	2 (2)	2	31.2 (NA, NA; -6.1, 68.4)	Moderate	0	0	NA	NA
Community support + supervision + any training	1 (0)	1	25.5	Very low	1 (0)	1	125.0	Low
Community support + strengthening infrastructure + supervision + other management techniques + any training	0	0	NA	NA	1 (0)	1	23.3	Very low
Group problem solving + other management techniques	0	0	NA	NA	1 (0)	1	22.8	Very low
Supervision only	1 (0)	1	22.6	Very low	0	0	NA	NA

Strategy	Practice outcomes expressed as a percentage					Continuous practice outcomes			
	No. of study comparisons (no. with low or moderate risk of bias)	No. of countries studied	Median MES based on unadjusted effect sizes (minimum, maximum)	GRADE quality of evidence		No. of study comparisons (no. with low or moderate risk of bias)	No. of countries studied	Median MES based on unadjusted effect sizes (minimum, maximum)	GRADE quality of evidence
Community support + patient support + strengthening infrastructure + HCP-directed financial incentives + supervision + other management techniques + any training	1 (0)	1	22.0	Very low		0	0	NA	NA
Strengthening infrastructure + any training	1 (0)	1	12.4	Very low		0	0	NA	NA
Group problem solving only	1 (0)	1	11.5	Very low		0	0	NA	NA
Community support + strengthening infrastructure + any training	1 (1)	1	8.9	Low		1 (1)	1	40.2	Moderate
Community support + supervision + other management techniques + any training	1 (1)	1	8.2	Moderate		0	0	NA	NA
Strengthening infrastructure + supervision + other management techniques + information and communication technology for HCPs	2 (2)	2	6.6 (NA, NA; 5.3, 7.8)	Moderate		0	0	NA	NA
Community support + strengthening infrastructure + health system financing and other incentives + supervision + other management techniques + printed information or job aid for HCPs + information and communication technology for HCPs	1 (1)	1	4.8	Moderate		0	0	NA	NA
Patient support + supervision + other management techniques + information and communication technology for HCPs	1 (0)	1	3.6	Very low		1 (0)	1	5.3	Very low

Footnotes.

GRADE = The Grading of Recommendations Assessment, Development, and Evaluation system, HCP = health care provider, MES = median effect size, NA = not applicable.

[a] Unless no studies with percentage outcomes were found, in which case results of continuous outcomes were used.

Table K. The effectiveness of strategies to improve health care provider performance for studies predominantly of lay or community health workers with at least one practice outcome: sensitivity analysis[a]

Strategy	Practice outcomes expressed as a percentage			Continuous practice outcomes		
	No. of study comparisons (no. with low or moderate risk of bias)	No. of countries studied	Median MES based on unadjusted effect sizes[b] (minimum, maximum)	No. of study comparisons (no. with low or moderate risk of bias)	No. of countries studied	Median MES based on unadjusted effect sizes[b] (minimum, maximum)
Strategies tested by at least three comparisons with percentage outcomes or at least three comparisons with continuous outcomes (descending order of effect size for percentage outcomes)						
Any training only	7 (2)	5	5.2 (-0.9, 5.6; -1.2, 24.0)	1 (1)	1	-25.0
Strategies tested by less than three comparisons with percentage outcomes and less than three comparisons with continuous outcomes (descending order of effect size for percentage outcomes[c])						
Community support + patient support + HCP-directed financial incentives + supervision + any training + information and communication technology for HCPs	0	0	NA	1 (0)	1	113.7
Supervision + information and communication technology for HCPs	0	0	NA	1 (1)	1	99.0
Strengthening infrastructure + health system financing and other incentives + supervision + other management techniques + any training	2 (0)	1	63.5 (NA, NA; 63.0, 64.0)	0	0	NA
Community support + strengthening infrastructure + HCP-directed financial incentives + health system financing and other incentives + regulation and governance + supervision + other management techniques + any training	1 (0)	1	61.4	0	0	NA

Strategy	Practice outcomes expressed as a percentage				Continuous practice outcomes		
	No. of study comparisons (no. with low or moderate risk of bias)	No. of countries studied	Median MES based on unadjusted effect sizes[b] (minimum, maximum)		No. of study comparisons (no. with low or moderate risk of bias)	No. of countries studied	Median MES based on unadjusted effect sizes[b] (minimum, maximum)
Supervision + other management techniques + any training	1 (0)	1	50.0		1(0)	1	58.1
Community support + regulation and governance + supervision + other management techniques + any training	1 (0)	1	39.8		0	0	NA
Community support + strengthening infrastructure + health system financing and other incentives + supervision + other management techniques + any training	1 (0)	1	34.5		0	0	NA
Community support + any training	2 (0)	2	33.2 (NA, NA; 10.2, 56.2)		0	0	NA
Information and communication technology for HCPs only	2 (2)	2	31.2 (NA, NA; -6.1, 68.4)		0	0	NA
Community support + supervision + any training	1 (0)	1	25.5		1 (0)	1	125.0
Community support + strengthening infrastructure + supervision + other management techniques + any training	0	0	NA		1 (0)	1	23.3
Group problem solving + other management techniques	0	0	NA		1 (0)	1	22.8
Community support + patient support + strengthening infrastructure + HCP-directed financial incentives + supervision + other management techniques + any training	1 (0)	1	22.0		0	0	NA

Strategy	Practice outcomes expressed as a percentage			Continuous practice outcomes		
	No. of study comparisons (no. with low or moderate risk of bias)	No. of countries studied	Median MES based on unadjusted effect sizes[b] (minimum, maximum)	No. of study comparisons (no. with low or moderate risk of bias)	No. of countries studied	Median MES based on unadjusted effect sizes[b] (minimum, maximum)
Patient support + printed information or job aid for HCPs	1 (0)	1	21.0	0	0	NA
Supervision only	2 (0)	2	20.8 (NA, NA; 19.0, 22.6)	0	0	NA
Strengthening infrastructure + any training	1 (0)	1	12.4	0	0	NA
Group problem solving only	1 (0)	1	11.5	0	0	NA
Regulation and governance + supervision + any training	1 (0)	1	10.0	0	0	NA
Community support + strengthening infrastructure + any training	2 (1)	2	9.6 (NA, NA; 8.9, 10.3)	1 (1)	1	40.2
Community support + supervision + other management techniques + any training	1 (1)	1	8.2	0	0	NA
Strengthening infrastructure + supervision + other management techniques + information and communication technology for HCPs	2 (2)	2	6.6 (NA, NA; 5.3, 7.8)	0	0	NA
Community support + strengthening infrastructure + health system financing and other incentives + regulation and governance + supervision + other management techniques + any training	1 (0)	1	6.0	0	0	NA

Strategy	Practice outcomes expressed as a percentage				Continuous practice outcomes		
	No. of study comparisons (no. with low or moderate risk of bias)	No. of countries studied	Median MES based on unadjusted effect sizes[b] (minimum, maximum)		No. of study comparisons (no. with low or moderate risk of bias)	No. of countries studied	Median MES based on unadjusted effect sizes[b] (minimum, maximum)
Community support + strengthening infrastructure + health system financing and other incentives + supervision + other management techniques + printed information or job aid for HCPs + information and communication technology for HCPs	1 (1)	1	4.8		0	0	NA
Supervision + any training	1 (1)	1	4.0		1 (1)	1	-2.2
Patient support + supervision + other management techniques + information and communication technology for HCPs	1 (0)	1	3.6		1 (0)	1	5.3
Supervision + printed information or job aid for HCPs	1 (1)	1	-0.1		1 (1)	1	-7.1

Footnotes.

HCP = health care provider, MES = median effect size, NA = not applicable.

[a] In this analysis, we used an alternative, expanded definition of lay HCPs that included some professional HCPs who sometimes have attributes of lay HCPs, such as Ethiopian Health Extension Workers, non-pharmacist drug vendors, and informal providers in the private sector.

[b] Effect sizes expressed as an absolute percentage-point change.

[c] Unless no studies with percentage outcomes were found, in which case results of continuous outcomes were used.

Table L1. The effectiveness of strategies to improve health care provider performance for studies of professional health care providers with at least one practice outcome and that had a low or moderate risk of bias

Strategy	Practice outcomes expressed as a percentage				Continuous practice outcomes			
	No. of study comparisons	No. of countries studied	Median MES based on adjusted effect sizes[a] (interquartile range; minimum, maximum)	GRADE quality of evidence	No. of study comparisons	No. of countries studied	Median MES based on unadjusted effect sizes[a] (interquartile range; minimum, maximum)	GRADE quality of evidence
Strategies tested by at least three comparisons with percentage outcomes or at least three comparisons with continuous outcomes (descending order of effect size for percentage outcomes)								
Group problem solving only	3	3	37.5 (NA, NA; 5.5, 61.2)	Moderate	0	0	NA	NA
Supervision + any training	11	5	18.8 (11.3, 24.7; 5.8, 30.8)	Moderate	3	2	-2.2 (NA, NA; -16.3, 7.3)	Moderate
Other management techniques + printed information or job aid for HCPs	2	2	18.3 (NA, NA; 4.7, 31.8)	Low	3	3	11.8 (NA, NA; 0.3, 16.5)	Moderate
Supervision only	8	6	15.9 (5.1, 25.2; 0.03, 40.4)	High	1	1	-90.4	Low
Other management techniques only	3	2	15.8 (NA, NA; -11.1, 17.3)	High	0	0	NA	NA
Patient support + any training	3	3	15.3 (NA, NA; 2.5, 26.9)	High	0	0	NA	NA
Strengthening infrastructure only	3	3	13.0 (NA, NA; -7.0, 15.8)	Moderate	2	2	152.1 (NA, NA; 4.2, 300.0)	Moderate
Any training only	33	16	10.3 (7.3, 20.7; -7.1, 50.6)	Moderate	8	7	17.4 (-7.7, 23.7; -25.0, 81.4)	Moderate
Group problem solving + information and communication technology for HCPs	3	3	6.7 (NA, NA; -3.5, 32.6)	High	0	0	NA	NA
Supervision + printed information or job aid for HCPs	3	7[b]	2.3 (NA, NA; 2.1, 24.4)	Moderate	1	1	-7.1	Low

Strategy	Practice outcomes expressed as a percentage				Continuous practice outcomes			
	No. of study comparisons	No. of countries studied	Median MES based on adjusted effect sizes[a] (interquartile range; minimum, maximum)	GRADE quality of evidence	No. of study comparisons	No. of countries studied	Median MES based on unadjusted effect sizes[a] (interquartile range; minimum, maximum)	GRADE quality of evidence
Printed information or job aid for HCPs only	5	5	1.4 (-4.7, 6.2; -4.9, 11.7)	High	1	1	6.5	Moderate
Information and communication technology for HCPs only	4	3	1.0 (-2.9, 9.9; -2.9, 15)	Moderate	1	1	-38.9	Low
Strengthening infrastructure + supervision + any training	1	1	-4.8	Moderate	4	3	64.3 (31.9, 88.7; 2.6, 110.1)	High
Strategies tested by less than three comparisons with percentage outcomes and less than three comparisons with continuous outcomes (descending order of effect size for percentage outcomes[c])								
Community support + strengthening infrastructure + supervision + other management techniques + any training	0	0	NA	NA	1	1	76.1	Moderate
Strengthening infrastructure + group problem solving	1	1	72.5	Moderate	0	0	NA	NA
Community support + HCP-directed financial incentives + other management techniques + any training + information and communication technology for HCPs	1	1	56.1	High	0	0	NA	NA
Other management techniques + any training	1	1	54.1	Moderate	0	0	NA	NA
Group problem solving + any training	1	1	52.6	Moderate	1	1	52.4	Moderate
Health system financing and other incentives only	0	0	NA	NA	2	2	46.4 (NA, NA; 20.4, 72.4)	Moderate

Strategy	Practice outcomes expressed as a percentage					Continuous practice outcomes			
	No. of study comparisons	No. of countries studied	Median MES based on adjusted effect sizes[a] (interquartile range; minimum, maximum)	GRADE quality of evidence		No. of study comparisons	No. of countries studied	Median MES based on unadjusted effect sizes[a] (interquartile range; minimum, maximum)	GRADE quality of evidence
Group problem solving + supervision + other management techniques + any training + information and communication technology for HCPs	1	2[b]	34.9	Moderate		0	0	NA	NA
Strengthening infrastructure + any training	1	1	33.1	Low		0	0	NA	NA
Strengthening infrastructure + supervision + other management techniques + any training	2	2	33.1 (Na, NA; 29.4, 36.7)	Low		1	1	56.9	Moderate
Patient support + strengthening infrastructure + supervision + other management techniques + any training	1	1	29.0	Moderate		0	0	NA	NA
Community support + patient support + strengthening infrastructure + regulation and governance + supervision + other management techniques + any training	1	1	27.6	Moderate		0	0	NA	NA
Community support + strengthening infrastructure + health system financing and other incentives + regulation and governance + any training	1	1	27.2	Moderate		0	0	NA	NA
Regulation and governance + supervision + other management techniques + any training	1	1	23.7	Low		0	0	NA	NA
Strengthening infrastructure + other management techniques + printed information or job aid for HCPs	1	1	23.2	Low		0	0	NA	NA

Strategy	Practice outcomes expressed as a percentage					Continuous practice outcomes			
	No. of study comparisons	No. of countries studied	Median MES based on adjusted effect sizes[a] (interquartile range; minimum, maximum)	GRADE quality of evidence		No. of study comparisons	No. of countries studied	Median MES based on unadjusted effect sizes[a] (interquartile range; minimum, maximum)	GRADE quality of evidence
Strengthening infrastructure + health system financing and other incentives + other management techniques + printed information or job aid for HCPs	1	1	22.9	Low		1	1	0.4	Low
Strengthening infrastructure + supervision	1	1	22.2	Low		1	1	47.9	Moderate
Community support + strengthening infrastructure + health system financing and other incentives + regulation and governance + supervision + any training	0	0	NA	NA		1	1	20.3	Low
Supervision + information and communication technology for HCPs	2	1	16.8 (NA, NA; 13.9, 19.8)	Low		1	1	36.4	Low
Group problem solving + supervision + any training	1	1	16.1	Low		1	1	58.1	High
Community support + strengthening infrastructure + health system financing and other incentives + supervision + any training + information and communication technology for HCPs	1	1	15.9	Moderate		0	0	NA	NA
Community support + other management techniques	1	1	15.8	Low		1	1	-6.0	Low
Community support + patient support + strengthening infrastructure + group problem solving + other management techniques + any training	1	1	14.7	Low		0	0	NA	NA

Strategy	Practice outcomes expressed as a percentage				Continuous practice outcomes			
	No. of study comparisons	No. of countries studied	Median MES based on adjusted effect sizes[a] (interquartile range; minimum, maximum)	GRADE quality of evidence	No. of study comparisons	No. of countries studied	Median MES based on unadjusted effect sizes[a] (interquartile range; minimum, maximum)	GRADE quality of evidence
Patient support + health system financing and other incentives + group problem solving + supervision + other management techniques + any training	1	1	14.3	Moderate	0	0	NA	NA
Community support + strengthening infrastructure + health system financing and other incentives + supervision + any training	1	1	14.1	Moderate	0	0	NA	NA
Supervision + other management techniques + any training	2	2	13.7<br>(NA, NA; 0.7, 26.7)	Low	2	2	30.1<br>(NA, NA; 28.3, 31.9)	Low
Strengthening infrastructure + health system financing and other incentives + any training	2	1	12.4<br>(Na, NA; 5.9, 18.9)	Low	0	0	NA	NA
Strengthening infrastructure + supervision + printed information or job aid for HCPs + information and communication technology for HCPs	1	1	12.1	Moderate	0	0	NA	NA
Strengthening infrastructure + other management techniques + printed information or job aid for HCPs + information and communication technology for HCPs	1	1	11.6	Low	0	0	NA	NA
HCP-directed financial incentives only	1	1	11.1	Moderate	1	1	66.7	High
Group problem solving + supervision + printed information or job aid for HCPs	2	2	10.0<br>(NA, NA; 6.2, 13.7)	Low	1	1	-6.5	Low

Strategy	Practice outcomes expressed as a percentage				Continuous practice outcomes			
	No. of study comparisons	No. of countries studied	Median MES based on adjusted effect sizes[a] (interquartile range; minimum, maximum)	GRADE quality of evidence	No. of study comparisons	No. of countries studied	Median MES based on unadjusted effect sizes[a] (interquartile range; minimum, maximum)	GRADE quality of evidence
Strengthening infrastructure + health system financing and other incentives + printed information or job aid for HCPs	1	1	9.4	Low	1	1	-25.4	Low
Group problems solving + printed information or job aid for HCPs	1	1	8.7	Moderate	0	0	NA	NA
Regulation and governance + supervision + other management techniques + any training + information and communication technology for HCPs	1	1	8.4	Moderate	0	0	NA	NA
Community support + supervision + any training	2	2	7.5 (NA, NA; -2.9, 17.9)	Low	0	0	NA	NA
Community support + patient support + strengthening infrastructure + HCP-directed financial incentives + supervision + any training	1	4[b]	7.0	Moderate	1	4[b]	0.0	Moderate
Strengthening infrastructure + regulation and governance + any training + information and communication technology for HCPs	1	1	6.0	Low	0	0	NA	NA
Patient support + supervision + any training	1	1	5.2	Low	0	0	NA	NA
Community support + any training	0	0	NA	NA	1	1	4.5	Low
Strengthening infrastructure + health system financing and other incentives + supervision + other management techniques + any training	1	1	4.4	Low	0	0	NA	NA

Strategy	Practice outcomes expressed as a percentage					Continuous practice outcomes			
	No. of study comparisons	No. of countries studied	Median MES based on adjusted effect sizes[a] (interquartile range; minimum, maximum)	GRADE quality of evidence		No. of study comparisons	No. of countries studied	Median MES based on unadjusted effect sizes[a] (interquartile range; minimum, maximum)	GRADE quality of evidence
Community support + regulation and governance + other management techniques	1	1	3.9	Low		0	0	NA	NA
Patient support + supervision + other management techniques + any training	1	1	3.3	Moderate		0	0	NA	NA
Regulation and governance + group problem solving	1	1	2.7	Low		0	0	NA	NA
Strengthening infrastructure + supervision + any training + information and communication technology for HCPs	2	2	-0.2 (NA, NA; -1.6, 1.3)	Low		0	0	NA	NA
Community support + strengthening infrastructure + regulation and governance + supervision	1	1	-0.8	Moderate		0	0	NA	NA
Patient support + other management techniques	1	1	-2.2	Moderate		1	1	-4.3	Moderate
Printed information or job aid for HCPs + information and communication technology for HCPs	1	1	-3.2	Low		0	0	NA	NA
Strengthening infrastructure + supervision + any training	1	1	-4.8	Low		0	0	NA	NA
Community support + HCP-directed financial incentives	0	0	NA	NA		1	1	-29.3	Moderate

Footnotes.

GRADE = The Grading of Recommendations Assessment, Development, and Evaluation system, HCP = health care provider, MES = median effect size, NA = not applicable.

[a] Effect sizes expressed as an absolute percentage-point change. See methods for details on adjustment.

[b] Multi-country study.

[c] Unless no studies with percentage outcomes were found, in which case results of continuous outcomes were used.

Table L2. The effectiveness of strategies to improve health care provider performance for studies of lay or community health workers with at least one practice outcome and that had a low or moderate risk of bias

Strategy	Practice outcomes expressed as a percentage					Continuous practice outcomes			
	No. of study comparisons	No. of countries studied	Median MES based on unadjusted effect sizes[a] (minimum, maximum)	GRADE quality of evidence		No. of study comparisons	No. of countries studied	Median MES based on unadjusted effect sizes[a] (minimum, maximum)	GRADE quality of evidence
Strategies tested by fewer than three comparisons with percentage outcomes and less than three comparisons with continuous outcomes (descending order of effect size for percentage outcomes[b])									
Supervision + information and communication technology for HCPs	0	0	NA	NA		1	1	99.0	Moderate
Information and communication technology for HCPs only	2	2	31.2<br>(NA, NA; -6.1, 68.4)	Low		0	0	NA	NA
Community support + strengthening infrastructure + any training	1	1	8.9	Low		1	1	40.2	Moderate
Community support + supervision + other management techniques + any training	1	1	8.2	Moderate		0	0	NA	NA
Strengthening infrastructure + supervision + other management techniques + information and communication technology for HCPs	2	2	6.6<br>(NA, NA; 5.3, 7.8)	Moderate		0	0	NA	NA
Community support + strengthening infrastructure + health system financing and other incentives + supervision + other management techniques + printed information or job aid for HCPs + information and communication technology for HCPs	1	1	4.8	Low		0	0	NA	NA

Footnotes.

GRADE = The Grading of Recommendations Assessment, Development, and Evaluation system, HCP = health care provider, MES = median effect size, NA = not applicable.

[a] Effect sizes expressed as an absolute percentage-point change.

[b] Unless no studies with percentage outcomes were found, in which case results of continuous outcomes were used.

Table M. Assessing strategy effectiveness by broadening strategy definitions to increase contextual and implementation diversity

Strategy	Practice outcomes expressed as a percentage				Continuous practice outcomes		
	No. of study com-parisons	No. of countries studied	Median MES based on adjusted effect sizes[a] (interquartile range; minimum, maximum)		No. of study com-parisons	No. of countries studied	Median MES based on unadjusted effect sizes[a] (interquartile range; minimum, maximum)
Strategies for professional HCPs							
Narrow definition (same as Table 3 in main article) Strengthening infrastructure + health system financing and other incentives + supervision + other management techniques + any training	3	2	57.7 (NA, NA; 4.4, 58.7)		0	0	NA
Broadened definition Strengthening infrastructure + health system financing and other incentives + supervision + other management techniques + any training **+/- other strategy components**	9	5	32.8 (6.6, 58.7; 4.4, 60.6)		2	2	57.0 (NA, NA; -7.4, 121.3)
Narrow definition (same as Table 3 in main article) Group problem solving + any training	4	2	56.0 (40.9, 68.6; 29.2, 77.8)		1	1	52.4
Broadened definition Group problem solving + any training **+/- other strategy components**	14	11	16.1 (10.2, 34.9; 1.5, 77.8)		3	2	52.4 (NA, NA; 7.3, 58.1)
Narrow definition (same as Table 3 in main article) Strengthening infrastructure + supervision + other management techniques + any training	2	2	33.1 (NA, NA; 29.4, 36.7)		4	4	183.2 (63.2, 456.3; 56.9, 615.5)
Broadened definition Strengthening infrastructure + supervision + other management techniques + any training **+/- other strategy components**	17	10	29.4 (10.7, 36.9; 4.4, 60.6)		9	7	76.1 (69.4, 297.1; -7.4, 615.5)

Strategy	Practice outcomes expressed as a percentage				Continuous practice outcomes		
	No. of study com-parisons	No. of countries studied	Median MES based on adjusted effect sizes[a] (interquartile range; minimum, maximum)		No. of study com-parisons	No. of countries studied	Median MES based on unadjusted effect sizes[a] (interquartile range; minimum, maximum)
Narrow definition (same as Table 3 in main article)<br>Group problem solving only	12	10	28.0<br>(12.1, 41.7; 5.5, 61.2)		4	3	-8.1<br>(-24.3, 44.2; -28.2, 84.1)
Broadened definition<br>Group problem solving **without training +/- other strategy components**	23	17	12.1<br>(6.2, 37.5; -3.5, 72.5)		8	6	3.5<br>(-2.2, 84.1; -28.2, 375.2)
Narrow definition (same as Table 3 in main article)<br>Community support + supervision + any training	4	4	20.7<br>(7.5, 24.3; -2.9, 25.3)		0	0	NA
Broadened definition<br>Community support + supervision + any training **+/- other strategy components**	17	14	23.4<br>(14.2, 34.5; -2.9, 60.6)		7	9	76.1<br>(20.3, 121.3; 0, 153.0)
Narrow definition (same as Table 3 in main article)<br>Supervision + any training	26	17	18.1<br>(6.0, 25.2; -2.7, 67.0)		8	5	11.1<br>(7.3, 60.4; -16.3, 101.1)
Broadened definition<br>Supervision + any training **+/- other strategy components**	87	36	14.8<br>(5.8, 27.6; -16.2, 67.0)		30	19	31.9<br>(10.5, 76.1; -16.3, 615.5)
Narrow definition (same as Table 3 in main article)<br>Strengthening infrastructure + supervision + any training	4	4	8.9<br>(-0.8, 39.8; -4.8, 64.9)		4	3	64.3<br>(31.9, 88.7; 2.6, 110.1)
Broadened definition<br>Strengthening infrastructure + supervision + any training **+/- other strategy components**	33	23	16.0<br>(9.6, 36.7; -4.8, 64.9)		16	14	73.9<br>(56.9, 153.0; -7.4, 615.5)

Strategy	Practice outcomes expressed as a percentage				Continuous practice outcomes		
	No. of study com-parisons	No. of countries studied	Median MES based on adjusted effect sizes[a] (interquartile range; minimum, maximum)		No. of study com-parisons	No. of countries studied	Median MES based on unadjusted effect sizes[a] (interquartile range; minimum, maximum)
Narrow definition (same as Table 3 in main article)<br>Information and communication technology for HCPs only	4	3	1.0<br>(-2.8, 9.9; -2.9, 15.1)		1	1	-38.9
Broadened definition<br>Information and communication technology for HCPs<br>**+/- other strategy components**	28	19	8.4<br>(4.7, 16.0; -3.5, 59.4)		4	4	-0.2<br>(-19.6, 18.2; -38.9, 36.4)
Narrow definition (same as Table J1 in Section 5)<br>HCP-directed financial incentives only	2	2	26.0<br>(NA, NA; 11.2, 40.8)		1	1	66.7
Broadened definition<br>HCP-directed financial incentives **+/- other strategy components**	10	12	7.2<br>(6.6, 40.8; 5.0, 60.6)		8	8	18.9<br>(3.5, 66.7; -29.3, 375.2)
Narrow definition (same as Table 3 in main article)<br>Health system financing and other incentives only	2	2	1.2<br>(NA, NA; -2.6, 5.0)		3	2	20.4<br>(NA, NA; -23.9, 72.4)
Broadened definition<br>Health system financing and other incentives<br>**+/- other strategy components**	38	21	14.2<br>(7.2, 28.8; -2.6, 60.6)		23	11	5.9<br>(-0.4, 20.4; -25.4, 375.2)
Narrow definition<br>Regulation and governance	0	0	NA		0	0	NA
Broadened definition<br>Regulation and governance **+/- other strategy components**	10	8	27.6<br>(3.9, 36.9; -0.8, 60.6)		5	4	20.3<br>(3.5, 70.0; 3.5, 121.3)

Strategy	Practice outcomes expressed as a percentage				Continuous practice outcomes		
	No. of study com-parisons	No. of countries studied	Median MES based on adjusted effect sizes[a] (interquartile range; minimum, maximum)		No. of study com-parisons	No. of countries studied	Median MES based on unadjusted effect sizes[a] (interquartile range; minimum, maximum)
Strategies for predominantly lay health workers (or community health workers)							
Narrow definition (same as Table J2 in Section 5)<br>Information and communication technology for HCPs only	2	2	31.2<br>(NA, NA; -6.1, 68.4)		0	0	NA
Broadened definition<br>Information and communication technology for HCPs **+/- other strategy components**	6	5	4.8<br>(3.6, 7.8; -6.1, 68.4)		3	3	99.0<br>(NA, NA; 5.3, 113.7)
Narrow definition (same as Table J2 in Section 5)<br>Community support + any training	1	1	56.2		0	0	NA
Broadened definition<br>Community support + any training **+/- other strategy components**	5	5	8.9<br>(8.2, 22.0; 8.2, 56.2)		4	3	76.9<br>(31.8, 119.3; 23.3, 125.0)

Footnotes.

HCP = Health care provider, MES = median effect size, NA = not applicable.

[a] Effect sizes expressed as an absolute percentage-point change. Among strategies for predominantly lay health workers, effect sizes were not adjusted. See methods section for details on adjustment.

Table N1. The effectiveness of strategies tested by at least three study comparisons each, for studies of professional health care providers with at least one practice outcome expressed as a percentage: stratification by low-resource versus moderate-resource setting where the study was conducted

Strategy	Median MES based on adjusted effect sizes[a] (interquartile range; minimum, maximum), no. of study comparisons	
	Low-resource settings[b]	Moderate-resource settings[b]
Median MES greater in moderate-resource settings by 10 %-points or more		
Group problem solving only	12.1<br>(8.9, 20.7; 5.5, 41.7)<br>6 comparisons	40.2<br>(31.7, 60.9; 28.0, 61.2)<br>6 comparisons
Patient support + any training	11.2<br>(NA, NA; -6.4, 15.3)<br>3 comparisons	26.9<br>(NA, NA; 2.6, 31.4)<br>3 comparisons
Supervision + any training	11.8<br>(3.2, 20.6; -2.0, 30.8)<br>15 comparisons	24.7<br>(13.4, 46.5; -2.7, 67.0)<br>11 comparisons
Difference in median MES less than 10 %-points		
Supervision only	7.5<br>(5.1, 16.2; -6.1, 27.6)<br>7 comparisons	14.8<br>(10.0, 27.6; 0.03, 56.3)<br>9 comparisons
Printed information or job aid for HCPs only	3.0<br>(NA, NA; -13.7, 6.6)<br>3 comparisons	1.4<br>(-4.7, 6.2; -4.8, 11.6)<br>5 comparisons
Any training only	9.0<br>(4.8, 27.0; -7.1, 54.3)<br>22 comparisons	10.3<br>(7.0, 18.0; -19.9, 60.8)<br>56 comparisons

Footnotes.

MES = Median effect size, NA = not applicable

[a] Effect sizes expressed as an absolute percentage-point change. See methods section for details on adjustment.

[b] Low-resource settings include non-hospital settings in low-income countries and rural only settings in middle-income countries. Moderate-resource settings include hospital settings in low income countries and places in middle-income countries that are not only rural.

Table N2. The effectiveness of strategies tested by at least three study comparisons each, for studies of professional health care providers with at least one practice outcome expressed as a percentage: stratification by low- versus middle-income country where the study was conducted

Strategy	Median MES based on adjusted effect sizes[a] (interquartile range; minimum, maximum), no. of study comparisons	
	Low-income country	Middle-income country[b]
Median MES greater in middle-income countries by 10 %-points or more		
Group problem solving only	12.1<br>(8.9, 20.7; 5.5, 41.7)<br>6 comparisons	40.2<br>(31.7, 60.9; 28.0, 61.2)<br>6 comparisons
Patient support + any training	11.2<br>(NA, NA; -6.4, 15.3)<br>3 comparisons	26.9<br>(NA, NA; 2.6, 31.4)<br>3 comparisons
Difference in median MES less than 10 %-points		
Supervision only	10.0<br>(6.2, 16.2; 0.5, 27.6)<br>8 comparisons	14.8<br>(11.3, 27.6; -6.1, 56.3)<br>8 comparisons
Printed information or job aid for HCPs only	-0.9<br>(-9.3, 4.8; -13.7, 6.6)<br>4 comparisons	3.8<br>(-1.6, 8.9; -4.7, 11.6)<br>4 comparisons
Supervision + any training	18.1<br>(3.2, 24.0; -2, 30.8)<br>12 comparisons	13.9<br>(11.8, 25.2; -2.7, 67.0)<br>14 comparisons
Any training only	7.5<br>(0.1, 15.7; -19.9, 54.3)<br>24 comparisons	10.3<br>(7.0, 20.7; -7.1, 60.8)<br>54 comparisons

Footnotes.

MES = Median effect size, NA = not applicable

[a] Effect sizes expressed as an absolute percentage-point change. See methods section for details on adjustment.

[b] One study of any training only was conducted in a mix of low- and middle-income countries (Argentina, Brazil, Chile, Colombia, and Costa Rica).

Table O. Sensitivity and secondary analyses on the effectiveness of strategies to improve health care provider performance for studies of professional health care providers with at least one practice outcome expressed as a percentage: meta-analyses of adjusted and unadjusted effect sizes, median analysis of unadjusted weighted effect sizes, and median analysis of unadjusted unweighted effect sizes

Strategy	No. of study compar-isons	Primary analysis: Median MES based on adjusted weighted effect sizes[a] (interquartile range; minimum, maximum)	Secondary analysis: Mean MES based on meta-analysis of adjusted effect sizes (95% CI) and I^2 heterogeneity statistic	Secondary analysis: Mean MES based on meta-analysis of unadjusted effect sizes (95% CI) and I^2 heterogeneity statistic	Sensitivity analysis: Median MES based on unadjusted weighted effect sizes[a] (interquartile range; minimum, maximum)	Sensitivity analysis: Median MES based on unadjusted unweighted effect sizes[a] (interquartile range; minimum, maximum)
Strategies tested by at least three comparisons each (descending order of effect size from primary analysis)						
Strengthening infrastructure + health system financing and other incentives + supervision + other management techniques + any training	3	57.7<br>(NA, NA; 4.4, 58.7)	40.3 (5.1, 75.4)<br>I^2 = 97%	45.0 (9.0, 81.1)<br>I^2 = 98%	63.0<br>(NA, NA; 8.1, 64.0)	63.0<br>(NA, NA; 8.1, 64.0)
Group problem solving + any training	4	56.0<br>(40.9, 68.6; 29.2, 77.8)	53.2 (41.1, 65.4)<br>I^2 = 69%	60.9 (48.4, 73.3)<br>I^2 = 72%	63.4<br>(48.1, 77.8; 35.3, 89.6)	63.4<br>(48.1, 77.8; 35.3, 89.6)
Group problem solving only	12	28.0<br>(12.1, 41.7; 5.5, 61.2)	29.8 (17.3, 42.3)<br>I^2 = 69%	34.5 (21.3, 47.8)<br>I^2 = 74%	30.1<br>(14.3, 51.7; 8.1, 71.2)	32.2<br>(17.0, 50.7; 8.1, 71.2)
Community support + supervision + any training	4	20.7<br>(7.5, 24.3; -2.9, 25.3)	14.6 (-0.7, 30.0)<br>I^2 = 81%	14.7 (0.6, 28.7)<br>I^2 = 78%	19.6<br>(8.9, 24.1; -1.8, 28.5)	19.6<br>(8.9, 24.1; -1.8, 28.5)
Supervision + any training	26	18.1<br>(6.0, 25.2; -2.7, 67)	18.1 (11.1, 25.1)<br>I^2 = 91%	20.1 (10.8, 29.5)<br>I^2 = 97%	14.5<br>(4.5, 30.0; 0.0, 73.3)	14.5<br>(5.0, 32.4; 0.0, 73.3)
Other management techniques only	4	16.5<br>(2.3, 21.3; -11.1, 25.3)	17.0 (5.3, 28.7)<br>I^2 = 38%	14.8 (-2.8, 32.5)<br>I^2 = 72%	10.4<br>(0.7, 22.0; -8.4, 33.0)	10.4<br>(0.7, 22.0; -8.4, 33.0)
Other management techniques + any training	5	15.9<br>(2.8, 23.9; -1.7, 54.2)	21.2 (3.8, 38.5)<br>I^2 = 80%	23.1 (6.3, 40.0)<br>I^2 = 79%	10.0<br>(10.0, 25.9; 9.2, 51.9)	13.7<br>(10.0, 25.9; 9.2, 51.9)
Community support + any training	4	15.1<br>(9.0, 25; 8.2, 29.6)	17.6 (8.4, 26.7)<br>I^2 = 0%	9.2 (0.4, 18.0)<br>I^2 = 0%	8.2<br>(5.2, 14.7; 4.3, 19.3)	8.2<br>(5.2, 14.7; 4.3, 19.3)

Section 5 – Additional results

Strategy	No. of study compar-isons	Primary analysis: Median MES based on adjusted weighted effect sizes[a] (interquartile range; minimum, maximum)	Secondary analysis: Mean MES based on meta-analysis of adjusted effect sizes (95% CI) and I^2 heterogeneity statistic	Secondary analysis: Mean MES based on meta-analysis of unadjusted effect sizes (95% CI) and I^2 heterogeneity statistic	Sensitivity analysis: Median MES based on unadjusted weighted effect sizes[a] (interquartile range; minimum, maximum)	Sensitivity analysis: Median MES based on unadjusted unweighted effect sizes[a] (interquartile range; minimum, maximum)
Supervision only	16	14.8<br>(6.2, 25.2; -6.1, 56.3)	18.1 (10.2, 26.0)<br>I^2 = 84%	18.9 (10.3, 27.5)<br>I^2 = 88%	11.7<br>(6.9, 23.7; 2.1, 67.8)	11.2<br>(6.6, 25.8; 2.1, 67.8)
Strengthening infrastructure only	3	13.0<br>(NA, NA; -7, 15.8)	9.8 (-1.0, 20.7)<br>I^2 = 30%	5.1 (-8.0, 18.2)<br>I^2 = 45%	2.0<br>(NA, NA; -10.0, 11.0)	2.0<br>(NA, NA; -10.0, 11.0)
Supervision + other management techniques + any training	5	11.4<br>(0.7, 11.4; -16.2, 26.7)	3.5 (-11.8, 18.8)<br>I^2 = 85%	10.2 (-3.5, 23.8)<br>I^2 = 84%	22.3<br>(2.9, 22.3; -5.5, 33.3)	2.9<br>(0.6, 22.3; -5.5, 33.3)
Patient support + any training	6	11.2<br>(2.6, 15.3; -6.4, 31.4)	8.6 (-1.9, 19.0)<br>I^2 = 91%	10.5 (-4.4, 25.4)<br>I^2 = 96%	15.1<br>(2.8, 20.5; -9.4, 25.1)	17.8<br>(2.8, 23.1; -9.4, 25.1)
Any training only	78	10.3<br>(6.1, 20.7; -19.9, 60.8)	13.7 (10.5, 16.9)<br>I^2 = 83%	13.4 (10.0, 16.8)<br>I^2 = 86%	9.6<br>(4.3, 20.5; -21.3, 68.1)	8.7<br>(4.0, 19.0; -21.3, 68.1)
Strengthening infrastructure + supervision + any training	4	8.9<br>(-0.8, 39.8; -4.8, 64.9)	17.6 (-5.2, 40.4)<br>I^2 = 89%	24.5 (0.6, 48.4)<br>I^2 = 91%	12.8<br>(5.7, 47.3; 3.3, 77.0)	12.8<br>(5.7, 47.3; 3.3, 77.0)
Supervision + other management techniques	4	7.7<br>(-1.3, 11.7; -7.9, 13.3)	10.2 (5.1, 15.3)<br>I^2 = 14%	3.9 (-0.3, 8.2)<br>I^2 = 0%	2.1<br>(1.6, 4.2; 1.5, 5.8)	2.1<br>(1.6, 4.2; 1.5, 5.8)
Group problem solving + information and communication technology for HCPs	3	6.7<br>(NA, NA; -3.5, 32.6)	11.4 (-8.9, 31.7)<br>I^2 = 40%	3.7 (-17.2, 24.6)<br>I^2 = 45%	-2.3<br>(NA, NA; -10.7, 25.8)	-2.3<br>(NA, NA; -10.7, 25.8)
Supervision + printed information or job aid for HCPs	3	2.3<br>(NA, NA; 2.1, 24.4)	10.3 (-7.7, 28.3)<br>I^2 = 96%	10.8 (-12.3, 33.9)<br>I^2 = 97%	1.9<br>(NA, NA; -0.1, 29.0)	1.9<br>(NA, NA; -0.1, 29.0)
Printed information or job aid for HCPs only	8	1.4<br>(-4.8, 6.2; -13.7, 11.6)	-2.8 (-9.3, 3.7)<br>I2 = 47%	-1.5 (-5.3, 2.3)<br>I^2 = 0%	2.5<br>(-0.7, 5.2; -5.5, 7.0)	1.6<br>(-0.5, 6.1; -5.5, 7.0)
Strengthening infrastructure + supervision + any training + information and communication technology for HCPs	3	1.3<br>(NA, NA; -1.7, 20.1)	2 (-9.0, 13.1)<br>I^2 = -60%	3.4 (-8.7, 15.6)<br>I^2 = 14%	3.9<br>(NA, NA; -3.8, 26.0)	3.9<br>(NA, NA; -3.8, 26.0)

Section 5 – Additional results

Strategy	No. of study compar-isons	Primary analysis: Median MES based on adjusted weighted effect sizes[a] (interquartile range; minimum, maximum)	Secondary analysis: Mean MES based on meta-analysis of adjusted effect sizes (95% CI) and I^2 heterogeneity statistic	Secondary analysis: Mean MES based on meta-analysis of unadjusted effect sizes (95% CI) and I^2 heterogeneity statistic	Sensitivity analysis: Median MES based on unadjusted weighted effect sizes[a] (interquartile range; minimum, maximum)	Sensitivity analysis: Median MES based on unadjusted unweighted effect sizes[a] (interquartile range; minimum, maximum)
Information and communication technology for HCPs only	4	1.0<br>(-2.8, 9.9; -2.9, 15.1)	0.6 (-6.6, 7.8)<br>I^2 = 37%	4.4 (-5.9, 14.7)<br>I^2 = 67%	1.9<br>(-2.4, 14.2; -4.7, 24.5)	1.9<br>(-2.4, 14.2; -4.7, 24.5)
Strategies tested by less than three comparisons each (descending order of effect size from primary analysis)						
Strengthening infrastructure + group problem solving	1	72.5	72.5 (40.7, 104.2)	69.8 (38.1, 101.5)	69.8	69.8
Community support + strengthening infrastructure + HCP-directed financial incentives + health system financing and other incentives + regulation and governance + supervision + other management techniques + any training	1	60.6	60.6 (49.7, 71.5)	61.4 (50.8, 72.0)	61.4	61.4
Supervision + any training + information and communication technology for HCPs	1	59.4	59.4 (14.3, 104.6)	68.9 (23.9, 113.9)	68.9	68.9
Community support + HCP-directed financial incentives + other management techniques + any training + information and communication technology for HCPs	1	56.1	56.1 (19.9, 92.2)	48.9 (12.9, 84.9)	48.9	48.9
Community support + regulation and governance + any training	1	40.0	40.0 (11.7, 68.4)	39.1 (10.9, 67.2)	39.0	39.0
Health system financing and other incentives + supervision + any training	1	39.4	39.4 (25.9, 53)	42.0 (28.6, 55.4)	42.0	42.0
Group problem solving + supervision + other management techniques + any training + information and communication technology for HCPs	1	34.9	34.9 (14.0, 55.9)	42.0 (21.3, 62.7)	42.0	42.0

Section 5 – Additional results

Strategy	No. of study compar-isons	Primary analysis: Median MES based on adjusted weighted effect sizes[a] (interquartile range; minimum, maximum)	Secondary analysis: Mean MES based on meta-analysis of adjusted effect sizes (95% CI) and I^2 heterogeneity statistic	Secondary analysis: Mean MES based on meta-analysis of unadjusted effect sizes (95% CI) and I^2 heterogeneity statistic	Sensitivity analysis: Median MES based on unadjusted weighted effect sizes[a] (interquartile range; minimum, maximum)	Sensitivity analysis: Median MES based on unadjusted unweighted effect sizes[a] (interquartile range; minimum, maximum)
Community support + regulation and governance + supervision other management techniques + any training	1	34.5	34.5 (25.2, 43.8)	39.8 (31.1, 48.5)	39.8	39.8
Strengthening infrastructure + supervision + other management techniques + any training	2	33.1<br>(NA, NA; 29.4, 36.7)	36.5 (30.8, 42.3)<br>I^2 = 0%	41.9 (36.2, 47.6)<br>I^2 = 0%	40.0<br>(NA, NA; 38.0, 42.0)	40.0<br>(NA, NA; 38.0, 42.0)
Strengthening infrastructure + health system financing and other incentives + group problem solving + supervision + other management techniques + any training	1	32.8	32.8 (12.3, 53.2)	36.5 (16.1, 56.9)	36.5	36.5
Community support + strengthening infrastructure + health system financing and other incentives + supervision + other management techniques + any training	1	32.6	32.6 (5.1, 60.1)	34.5 (7.1, 61.9)	34.5	34.5
Community support + patient support + strengthening infrastructure + regulation and governance + supervision + other management techniques + any training	2	32.2<br>(NA, NA; 27.6, 36.9)	32.3 (23.2, 41.4)<br>I^2 = 83%	34.6 (23.6, 45.6)<br>I^2 = 90%	34.6<br>(NA, NA; 29.0, 40.1)	34.6<br>(NA, NA; 29.0, 40.1)
Patient support + other management techniques + printed information or job aid for HCPs	1	29.4	29.4 (14.2, 44.5)	32.4 (17.5, 47.3)	32.4	32.4
Patient support + strengthening infrastructure + supervision + other management techniques + any training	1	29.0	29.0 (7.4, 50.7)	30.0 (8.4, 51.6)	30.0	30.0
Health system financing and other incentives + regulation and governance + supervision + other management techniques	1	28.8	28.8 (19.4, 38.2)	24.5 (15.7, 33.3)	24.5	24.5

Strategy	No. of study compar-isons	Primary analysis: Median MES based on adjusted weighted effect sizes[a] (interquartile range; minimum, maximum)	Secondary analysis: Mean MES based on meta-analysis of adjusted effect sizes (95% CI) and I^2 heterogeneity statistic	Secondary analysis: Mean MES based on meta-analysis of unadjusted effect sizes (95% CI) and I^2 heterogeneity statistic	Sensitivity analysis: Median MES based on unadjusted weighted effect sizes[a] (interquartile range; minimum, maximum)	Sensitivity analysis: Median MES based on unadjusted unweighted effect sizes[a] (interquartile range; minimum, maximum)
Community support + strengthening infrastructure + health system financing and other incentives + regulation and governance + any training	1	27.2	27.2 (11.5, 42.9)	25.6 (10.1, 41.0)	25.6	25.6
Community support + strengthening infrastructure + supervision + other management techniques + any training	1	26.9	26.9 (6.8, 47.0)	26.5 (6.5, 46.5)	26.5	26.5
Community support + strengthening infrastructure + health system financing and other incentives + supervision + any training	2	26.2<br>(NA, NA; 14.2, 38.3)	17.6 (1.0, 34.2)<br>I^2 = 22%	24.5 (0.3, 48.7)<br>I^2 = 45%	31.8<br>(NA, NA; 17.5, 46.1)	31.8<br>(NA, NA; 17.5, 46.1)
HCP-directed financial incentives only	2	26.0<br>(NA, NA; 11.2, 40.8)	27.8 (-1.0, 56.6)<br>I^2 = 84%	27.5 (-4.0, 59.0)<br>I^2 = 87%	25.8<br>(NA, NA; 9.7, 42.0)	25.8<br>(NA, NA; 9.7, 42.0)
Any training + information and communication technology for HCPs	2	25.7<br>(NA, NA; 8.1, 43.3)	28.5 (-5.6, 62.6)<br>I^2 = 64%	29.0 (-15.3, 73.4)<br>I^2 = 78%	26.8<br>(NA, NA; 4.1, 49.5)	26.8<br>(NA, NA; 4.1, 49.5)
Health system financing and other incentives + regulation and governance + supervision	1	24.6	24.6 (16.0, 33.1)	19.9 (12.0, 27.8)	19.9	19.9
Group problem solving + other management techniques	2	24.4<br>(NA, NA; 9.4, 39.4)	21.3 (-7.5, 50.1)<br>I^2 = 79%	21.0 (-18.2, 60.2)<br>I^2 = 88%	23.3<br>(NA, NA; 3.2, 43.4)	23.3<br>(NA, NA; 3.2, 43.4)
Strengthening infrastructure + other management techniques + printed information or job aid for HCPs	1	23.2	23.2 (15.2, 31.2)	20.2 (12.7, 27.7)	20.2	20.2
Community support + strengthening infrastructure + health system financing and other incentives + any training	1	21.2	21.2 (3.3, 39.1)	22.7 (5.0, 40.4)	22.7	22.7
Health system financing and other incentives + any training	2	20.8<br>(NA, NA; 11.4, 30.2)	18.8 (0.5, 37.1)<br>I^2 = 0%	20.0 (1.8, 38.3)<br>I^2 = 0%	21.3<br>(NA, NA; 15.2, 27.5)	21.3<br>(NA, NA; 15.2, 27.5)

Strategy	No. of study compar-isons	Primary analysis: Median MES based on adjusted weighted effect sizes[a] (interquartile range; minimum, maximum)	Secondary analysis: Mean MES based on meta-analysis of adjusted effect sizes (95% CI) and I^2 heterogeneity statistic	Secondary analysis: Mean MES based on meta-analysis of unadjusted effect sizes (95% CI) and I^2 heterogeneity statistic	Sensitivity analysis: Median MES based on unadjusted weighted effect sizes[a] (interquartile range; minimum, maximum)	Sensitivity analysis: Median MES based on unadjusted unweighted effect sizes[a] (interquartile range; minimum, maximum)
Other management techniques + printed information or job aid for HCPs	2	18.2<br>(NA, NA; 4.7, 31.8)	17.7 (-8.9, 44.3)<br>I^2 = 86%	16.8 (-6.9, 40.5)<br>I^2 = 82%	17.4<br>(NA, NA; 5.3, 29.5)	17.4<br>(NA, NA; 5.3, 29.5)
Patient support + supervision + other management techniques + any training	2	17.7<br>(NA, NA; 3.3, 32.1)	16.4 (-11.6, 44.5)<br>I^2 = 88%	21.4 (-10.9, 53.7)<br>I^2 = 91%	22.5<br>(NA, NA; 6.0, 39.0)	22.5<br>(NA, NA; 6.0, 39.0)
Strengthening infrastructure + any training	2	17.7<br>(NA, NA; 2.2, 33.2)	16.3 (-13.9, 46.6)<br>I^2 = 73%	17.6 (-21.2, 56.3)<br>I^2 = 83%	18.6<br>(NA, NA; -1.2, 38.4)	18.6<br>(NA, NA; -1.2, 38.4)
Patient support + strengthening infrastructure + regulation and governance + supervision + other management techniques + any training	1	17.3	17.3 (-16.3, 50.8)	14.7 (-18.8, 48.2)	14.7	14.7
Supervision + information and communication technology for HCPs	2	16.8<br>(NA, NA; 13.9, 19.8)	14.1 (6.9, 21.4)<br>I^2 = 0%	11.8 (5.5, 18.1)<br>I^2 = 0%	16.5<br>(NA, NA; 11.5, 21.5)	16.5<br>(NA, NA; 11.5, 21.5)
Community support + strengthening infrastructure + health system financing and other incentives + supervision + any training + information and communication technology for HCPs	1	16.0	16 (10.1, 21.8)	15.5 (10.2, 20.8)	15.5	15.5
Community support + other management techniques	1	15.7	15.7 (-12.7, 44.2)	18.2 (-10.1, 46.5)	18.2	18.2
Patient support + printed information or job aid for HCPs	2	15.4<br>(NA, NA; 2.2, 28.5)	14.2 (-11.4, 39.9)<br>I^2 = 85%	9.5 (-10.1, 29.0)<br>I^2 = 76%	10.9<br>(NA, NA; 0.8, 21.0)	10.9<br>(NA, NA; 0.8, 21.0)
Community support + patient support + other management techniques + printed information or job aid for HCPs	1	15.2	15.2 (7.0, 23.3)	10.9 (3.3, 18.5)	10.9	10.9
Strengthening infrastructure + group problem solving + supervision + any training	2	14.8<br>(NA, NA; 12.4, 17.3)	14.6 (-13.6, 42.8)<br>I^2 = 0%	16.6 (-11.5, 44.8)<br>I^2 = 0%	16.8<br>(NA, NA; 14.3, 19.3)	16.8<br>(NA, NA; 14.3, 19.3)

Strategy	No. of study compar-isons	Primary analysis: Median MES based on adjusted weighted effect sizes[a] (interquartile range; minimum, maximum)	Secondary analysis: Mean MES based on meta-analysis of adjusted effect sizes (95% CI) and I^2 heterogeneity statistic	Secondary analysis: Mean MES based on meta-analysis of unadjusted effect sizes (95% CI) and I^2 heterogeneity statistic	Sensitivity analysis: Median MES based on unadjusted weighted effect sizes[a] (interquartile range; minimum, maximum)	Sensitivity analysis: Median MES based on unadjusted unweighted effect sizes[a] (interquartile range; minimum, maximum)
Community support + patient support + strengthening infrastructure + group problem solving + other management techniques + any training	1	14.7	14.7 (-8.8, 38.2)	8.8 (-14.6, 32.2)	8.8	8.8
Patient support + health system financing and other incentives + group problem solving + supervision + other management techniques + any training	1	14.3	14.3 (7.8, 20.8)	20 (13.5, 26.4)	19.9	19.9
Community support + strengthening infrastructure + any training	2	14<br>(NA, NA; 5.5, 22.6)	13.2 (-3.4, 29.9)<br>I^2 = 88%	10.6 (7.4, 13.8)<br>I^2 = 0%	12.2<br>(NA, NA; 10.3, 14.0)	12.2<br>(NA, NA; 10.3, 14.0)
Strengthening infrastructure + health system financing and other incentives + supervision + any training	1	13.4	13.4 (8.8, 17.9)	5.1 (0.9, 9.3)	5.1	5.1
Regulation and governance + supervision + other management techniques + any training	2	13.3<br>(NA, NA; 2.8, 23.7)	13.9 (-6.6, 34.3)<br>I^2 = 92%	10.9 (-6.1, 27.9)<br>I^2 = 90%	10.3<br>(NA, NA; 1.6, 19.0)	10.3<br>(NA, NA; 1.6, 19.0)
Health system financing and other incentives + information and communication technology for HCPs	2	13.1<br>(NA, NA; 11.5, 14.6)	13.1 (-2.5, 28.6)<br>I^2 = 0%	4 (-11.4, 19.4)<br>I^2 = 0%	4.0<br>(NA, NA; 2.0, 6.0)	4.0<br>(NA, NA; 2.0, 6.0)
Strengthening infrastructure + health system financing and other incentives + any training	2	12.4<br>(NA, NA; 5.9, 18.9)	12.4 (-5.2, 30)<br>I^2 = 0%	11.5 (-6, 29.1)<br>I^2 = 0%	11.5<br>(NA, NA; 5.0, 18.0)	11.5<br>(NA, NA; 5.0, 18.0)
Strengthening infrastructure + health system financing and other incentives + other management techniques + printed information or job aid for HCPs	2	12.3<br>(NA, NA; 1.7, 22.8)	12.1 (-8.6, 32.8)<br>I^2 = 92%	13.3 (-7.2, 33.7)<br>I^2 = 92%	13.4<br>(NA, NA; 3.0, 23.9)	13.4<br>(NA, NA; 3.0, 23.9)
Strengthening infrastructure + supervision + printed information or job aid for HCPs + information and communication technology for HCPs	1	12.2	12.2 (-9.5, 33.8)	16.3 (-5.1, 37.7)	16.3	16.3

Section 5 – Additional results

Strategy	No. of study compar-isons	Primary analysis: Median MES based on adjusted weighted effect sizes[a] (interquartile range; minimum, maximum)	Secondary analysis: Mean MES based on meta-analysis of adjusted effect sizes (95% CI) and I^2 heterogeneity statistic	Secondary analysis: Mean MES based on meta-analysis of unadjusted effect sizes (95% CI) and I^2 heterogeneity statistic	Sensitivity analysis: Median MES based on unadjusted weighted effect sizes[a] (interquartile range; minimum, maximum)	Sensitivity analysis: Median MES based on unadjusted unweighted effect sizes[a] (interquartile range; minimum, maximum)
Supervision + any training + printed information or job aid for HCPs	1	11.9	11.9 (-12.6, 36.4)	17.1 (-7.1, 41.3)	17.1	17.1
Strengthening infrastructure + other management techniques + printed information or job aid for HCPs + information and communication technology for HCPs	1	11.5	11.5 (-15.4, 38.5)	5 (-21.8, 31.8)	5	5
Strengthening infrastructure + HCP-directed financial incentives + health system financing and other incentives + regulation and governance	1	11.5	11.5 (7.5, 15.5)	4.1 (2.4, 5.8)	4.1	4.1
Patient support + strengthening infrastructure + health system financing and other incentives + supervision + other management techniques + any training	1	10.7	10.7 (-12.5, 33.9)	1.4 (-21.7, 24.5)	1.4	1.4
Community support + strengthening infrastructure + group problem solving + supervision + other management techniques + any training	1	10.2	10.2 (-10.5, 30.9)	4.8 (-15.7, 25.2)	4.7	4.7
Group problem solving + supervision + printed information or job aid for HCPs	2	9.9<br>(NA, NA; 6.2, 13.7)	6.5 (-0.6, 13.6)<br>$I^2 = 0\%$	5.5 (-1.4, 12.4)<br>$I^2 = 0\%$	7.7<br>(NA, NA; 5.3, 10.1)	7.7<br>(NA, NA; 5.3, 10.1)
Community support + strengthening infrastructure + other management techniques + any training	1	9.7	9.7 (-7.7, 27.1)	6.6 (-10.7, 23.9)	6.6	6.6
Patient support + strengthening infrastructure + supervision + any training	1	9.6	9.6 (-9.8, 29)	9.5 (-9.8, 28.7)	9.5	9.5
Strengthening infrastructure + health system financing and other incentives + printed information or job aid for HCPs	1	9.3	9.3 (0.3, 18.4)	5.7 (-3.1, 14.5)	5.7	5.7

Section 5 – Additional results

Strategy	No. of study compar-isons	Primary analysis: Median MES based on adjusted weighted effect sizes[a] (interquartile range; minimum, maximum)	Secondary analysis: Mean MES based on meta-analysis of adjusted effect sizes (95% CI) and I^2 heterogeneity statistic	Secondary analysis: Mean MES based on meta-analysis of unadjusted effect sizes (95% CI) and I^2 heterogeneity statistic	Sensitivity analysis: Median MES based on unadjusted weighted effect sizes[a] (interquartile range; minimum, maximum)	Sensitivity analysis: Median MES based on unadjusted unweighted effect sizes[a] (interquartile range; minimum, maximum)
Strengthening infrastructure + health system financing and other incentives + regulation and governance	1	9.2	9.2 (-10.6, 29.1)	9.5 (-10.3, 29.2)	9.5	9.5
Group problem solving + supervision + any training	2	8.8<br>(NA, NA; 1.5, 16.1)	8.8 (-5.5, 23.2)<br>I^2 = 95%	10.6 (-10.1, 31.3)<br>I^2 = 98%	10.6<br>(NA, NA; 0, 21.1)	10.6<br>(NA, NA; 0, 21.1)
Group problem solving + printed information or job aid for HCPs	1	8.7	8.7 (-12.3, 29.6)	7.1 (-13.7, 27.9)	7.1	7.1
Regulation and governance + supervision + any training	2	8.4<br>(NA, NA; 4.9, 11.9)	5.7 (0.7, 10.7)<br>I^2 = 0%	10.0 (6.0, 13.9)<br>I^2 = 0%	9.8<br>(NA, NA; 9.5, 10.0)	9.8<br>(NA, NA; 9.5, 10.0)
Regulation and governance + supervision + other management techniques + any training + information and communication technology for HCPs	1	8.4	8.4 (3.1, 13.7)	4.5 (-0.6, 9.6)	4.5	4.5
Patient support + strengthening infrastructure + health system financing and other incentives + supervision + any training	1	8.0	8.0 (-7.1, 23)	2.4 (-12.5, 17.3)	2.4	2.4
Community support + strengthening infrastructure + health system financing and other incentives + regulation and governance + supervision + other management techniques + any training	1	7.2	7.2 (-4.8, 19.3)	6 (-5.8, 17.8)	6.0	6.0
HCP-directed financial incentives + health system financing and other incentives + supervision + any training	1	7.2	7.2 (-18.2, 32.6)	9.2 (-16.1, 34.5)	9.2	9.2
Community support + patient support + strengthening infrastructure + HCP-directed financial incentives + supervision + any training	1	7.0	7 (-9.4, 23.3)	-1.3 (-17.6, 15.0)	-1.3	-1.3

Section 5 – Additional results

Strategy	No. of study compar-isons	Primary analysis: Median MES based on adjusted weighted effect sizes[a] (interquartile range; minimum, maximum)		Secondary analysis: Mean MES based on meta-analysis of adjusted effect sizes (95% CI) and I^2 heterogeneity statistic		Secondary analysis: Mean MES based on meta-analysis of unadjusted effect sizes (95% CI) and I^2 heterogeneity statistic		Sensitivity analysis: Median MES based on unadjusted weighted effect sizes[a] (interquartile range; minimum, maximum)		Sensitivity analysis: Median MES based on unadjusted unweighted effect sizes[a] (interquartile range; minimum, maximum)
Strengthening infrastructure + supervision	2	6.9<br>(NA, NA; -8.5, 22.2)		7.6 (-22.5, 37.6)<br>I^2 = 85%		6.1 (-22.0, 34.2)<br>I^2 = 84%		5.4<br>(NA, NA; -9.0, 19.7)		5.4<br>(NA, NA; -9.0, 19.7)
Health system financing and other incentives + supervision	1	6.6		6.6 (-1.5, 14.8)		2.3 (-5.2, 9.7)		2.3		2.3
Community support + strengthening infrastructure + HCP-directed financial incentives + health system financing and other incentives + supervision + other management techniques + any training	1	6.6		6.6 (3, 10.2)		0.1 (-3.1, 3.2)		0.1		0.1
HCP-directed financial incentives + information and communication technology for HCPs	1	6.6		6.6 (1.2, 11.9)		-1.5 (-6.2, 3.2)		-1.5		-1.5
Patient support + supervision + any training	2	6.4<br>(NA, NA; 5.2, 7.5)		7.3 (1.9, 12.8)<br>I^2 = 0%		8.1 (2.9, 13.3)<br>I^2 = 0%		10.3<br>(NA, NA; 7.8, 12.9)		10.3<br>(NA, NA; 7.8, 12.9)
Strengthening infrastructure + regulation and governance + any training + information and communication technology for HCPs	1	6.0		6.0 (2.6, 9.4)		2.5 (-0.8, 5.8)		2.5		2.5
Group problem solving + supervision + other management technique + any training	1	5.6		5.6 (-11.9, 23.2)		14.7 (-2.7, 32.1)		14.7		14.7
Community support + strengthening infrastructure + health system financing and other incentives	1	5.4		5.4 (-6.2, 17.1)		0.7 (-10.9, 12.2)		0.7		0.7
HCP-directed financial incentives + health system financing and other incentives + information and communication technology for HCPs	1	5.0		5.0 (-18.7, 28.7)		6.0 (-17.6, 29.6)		6.0		6.0

Section 5 – Additional results

Strategy	No. of study compar-isons	Primary analysis: Median MES based on adjusted weighted effect sizes[a] (interquartile range; minimum, maximum)	Secondary analysis: Mean MES based on meta-analysis of adjusted effect sizes (95% CI) and I^2 heterogeneity statistic	Secondary analysis: Mean MES based on meta-analysis of unadjusted effect sizes (95% CI) and I^2 heterogeneity statistic	Sensitivity analysis: Median MES based on unadjusted weighted effect sizes[a] (interquartile range; minimum, maximum)	Sensitivity analysis: Median MES based on unadjusted unweighted effect sizes[a] (interquartile range; minimum, maximum)
Patient support + other management techniques	2	4.7<br>(NA, NA; -2.1, 11.4)	2.7 (-11.8, 17.1)<br>I^2 = 0%	6.2 (-9.5, 21.8)<br>I^2 = 14%	8.3<br>(NA, NA; 0, 16.5)	8.3<br>(NA, NA; 0, 16.5)
Community support + regulation and governance + other management techniques	1	3.9	3.9 (-2.6, 10.4)	8.0 (2.1, 13.9)	8	8
Printed information or job aid for HCPs + information and communication technology for HCPs	2	3.5<br>(NA, NA; -3.2, 10.2)	6.3 (-11.4, 23.9)<br>I^2 = 0%	5.1 (-12.4, 22.7)<br>I^2 = 0%	2.4<br>(NA, NA; -4.2, 9.0)	2.4<br>(NA, NA; -4.2, 9.0)
Patient support + strengthening infrastructure + regulation and governance + other management techniques + printed information or job aid for HCPs	1	2.9	2.9 (-31.9, 37.7)	-0.8 (-35.5, 34.0)	-0.8	-0.8
Regulation and governance + group problem solving	1	2.7	2.7 (-21.3, 26.6)	4.2 (-19.7, 28.1)	4.2	4.2
Health system financing and other incentives only	2	1.2<br>(NA, NA; -2.6, 5)	0 (-9.1, 9.1)<br>I^2 = 0%	3.7 (-5.2, 12.6)<br>I^2 = 0%	3.9<br>(NA, NA; 3.3, 4.5)	3.9<br>(NA, NA; 3.3, 4.5)
Community support + health system financing and other incentives + regulation and governance	1	1.2	1.2 (-13.6, 16)	-2.5 (-17.2, 12.2)	-2.5	-2.5
Community support + strengthening infrastructure + regulation and governance + supervision	1	-0.8	-0.8 (-3.7, 2.0)	0.8 (-1.1, 2.7)	0.8	0.8
Strengthening infrastructure + group problem solving + supervision + other management techniques	1	-3.1	-3.1 (-59.0, 52.9)	-5.6 (-61.5, 50.3)	-5.6	-5.6
Community support + strengthening infrastructure	2	-3.5<br>(NA, NA; -8.4, 1.4)	-6.1 (-15.8, 3.6)<br>I^2 = 0%	2.6 (-6.8, 12.1)<br>I^2 = 0%	5.7<br>(NA, NA; 0.05, 11.3)	5.7<br>(NA, NA; 0.05, 11.3)

Footnotes.

CI = Confidence interval, HCP = health care provider, MES = median effect size, NA = not applicable.

[a] Effect sizes expressed as an absolute percentage-point change. See methods section for details on adjustment.

Comments on Table O.

We found that using unadjusted effect sizes had a small impact on the effects for most strategies (median absolute difference of 3.5 %-points), as would be expected when baseline values were close to the mean of 40.1%. Also as expected, without adjustment, some effects became more extreme. For example, the strategy of supervision plus any training plus information and communication technology for HCPs (Table O, row 23), which had a baseline of 15.7% and a public facility only setting in a non-Asian country, had a median MES of 59.4 %-points in the adjusted analysis and 68.9 %-points in the unadjusted analysis. With analysis weights conservatively designed to avoid large between-study variation, unweighted analyses were generally similar to weighted analyses.

For the secondary analysis, which used random-effects meta-analysis for percentage outcomes only, we generally found results that were similar to those from the primary analysis of median MES in Table 3. Notable differences between the primary and secondary analyses were as follows.

1) The effect of "strengthening infrastructure + health system financing and other incentives + supervision + other management techniques + any training" (Table O, row 1) was lower with meta-analysis (40.3 %-points, compared to 57.7 %-points from the primary analysis, n = 3 study comparisons).
2) The effect of "supervision + other management techniques + any training" (row 11) was lower with meta-analysis (3.5 %-points, compared to 11.4 %-points from the primary analysis, n = 5 study comparisons).
3) The effect of "other management techniques + any training" (row 7) was higher with meta-analysis (21.2 %-points, compared to 15.9 %-points from the primary analysis, n = 5 study comparisons).
4) The effect of "strengthening infrastructure + supervision + any training" (row 14) was higher with meta-analysis (17.6 %-points, compared to 8.9 %-points from the primary analysis, n = 4 study comparisons).
5) The effect of "supervision + printed information or job aid for HCPs" (row 17) was higher with meta-analysis (10.3 %-points, compared to 2.3 %-points from the primary analysis, n = 3 study comparisons).

Of the 20 strategies tested by at least three study comparisons with percentage outcomes, 10 (50.0%) had 95% CIs that excluded zero (rows 1–5, 7–10, 13, and 15, "meta-analysis of adjusted effect sizes" column, of Table O). Of the 81 strategies tested by fewer than three study comparisons with percentage outcomes, 35 (43.2%) had 95% CIs that excluded zero.

Table P. The effectiveness of strategies evaluated in equivalency studies with a gold standard control group

Strategy	Gold standard control	Intervention group	Median effect size (%-points)	Comment
Studies of professional HCPs				
Any training	Physicians perform tubal ligation	Nurse-midwives perform tubal ligation	0[a]	Success: intervention was equivalent to gold standard control
Community support + patient support + supervision + other management techniques[b] + any training	Usual vaccine services	Integrating family planning into routine vaccination services[c]	9.2[d]	Success: intervention was better than gold standard control
Health system finances and other incentives + supervision + any training	Health care services with usual consultation and drug fee[e]	Consultation fees were removed, and training and supervision were added	1.7[a]	Success: intervention was essentially equivalent to gold standard control
			1.8[d]	Success: intervention was essentially equivalent to gold standard control
Study of lay or community health workers				
Supervision was removed	HCPs received reminders, and if HCP was overdue for patient visit, the supervisor was contacted	The step of contacting the supervisor was removed[f]	-151.3[d]	Not a success: intervention was worse than gold standard control

Footnotes.

HCP = Health care provider, %-point = percentage point.

[a] Percentage outcomes.

[b] Integration of services.

[c] The concern was that the addition of family planning could negatively impact vaccination services.

[d] Continuous outcomes.

[e] At baseline, HCPs received bonuses and other benefits derived from user fees. So when the fees were removed (and the bonuses ceased), the concern was that quality would diminish.

[f] The concern was that if HCPs knew that supervisors would not contact them for overdue patient visits, then HCPs' attentiveness to conducting patient visits would decline.

Figure A. Study follow-up times[a] for 317 studies that reported study duration

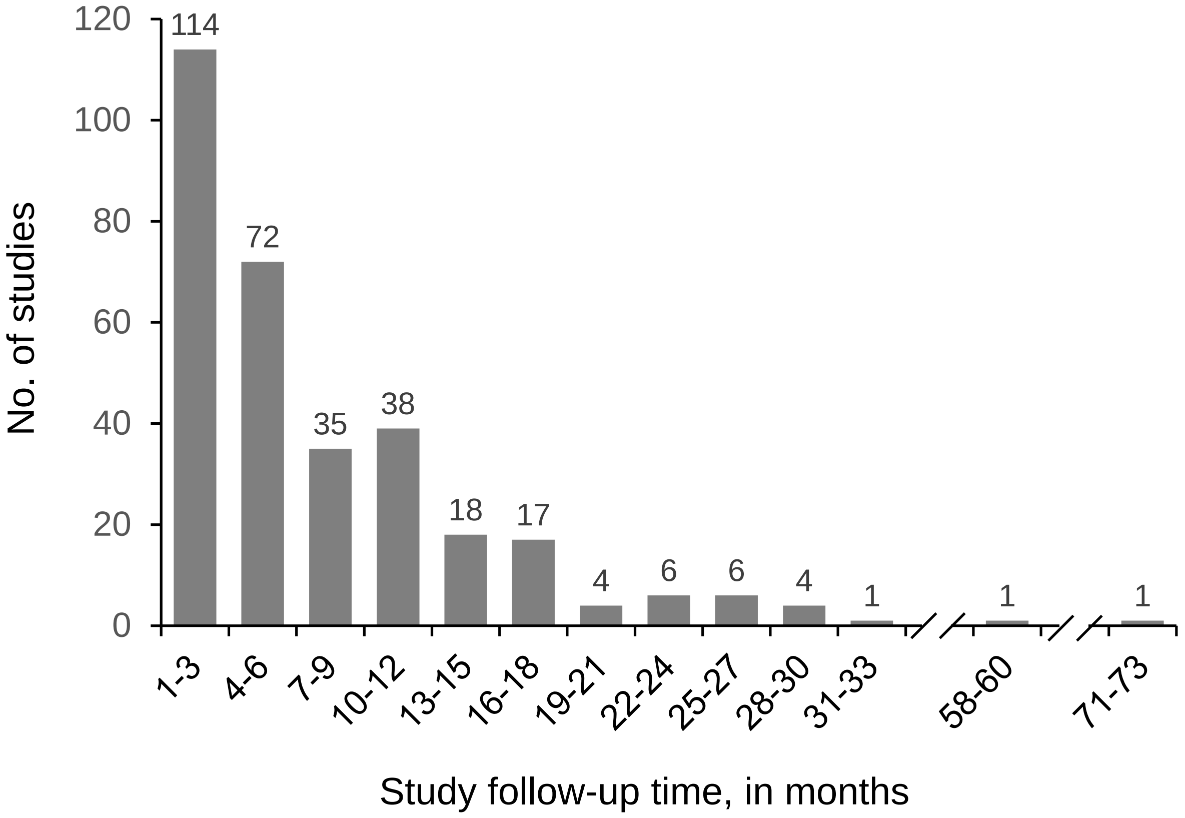

Footnotes.

[a] Follow-up time for a given study is the maximum follow-up time for measurement of all primary outcomes in the study.

Figure B. Effect size for practice outcome expressed as a percentage as a function of baseline performance level

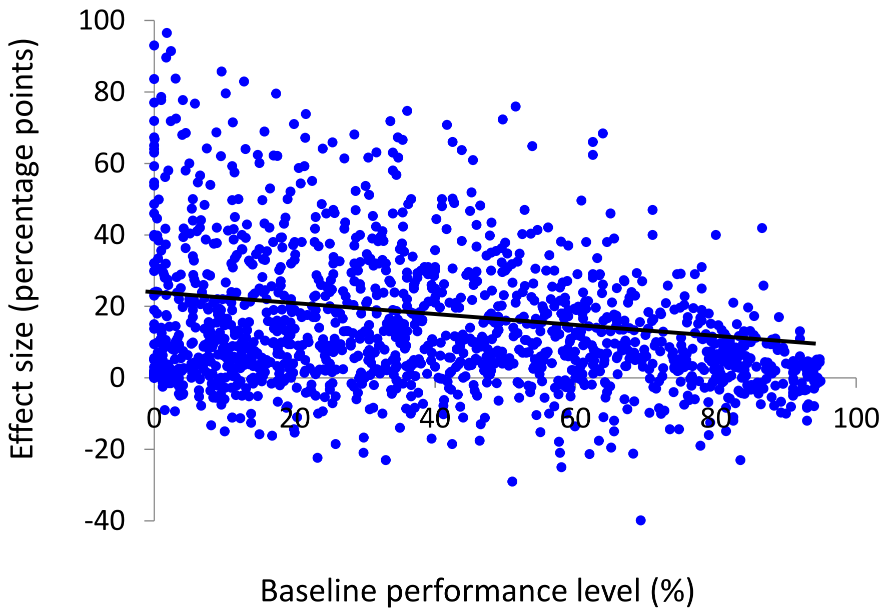

Footnotes

1. Analysis of 1475 effect sizes

2. Baseline performance level = the average of baseline values of intervention and control groups

3. Univariate regression line is: Effect size = 23.4 – 0.2 x baseline level

Figure C1. Effect size distributions for practice outcomes expressed as a percentage as a function of the number of components in the strategy

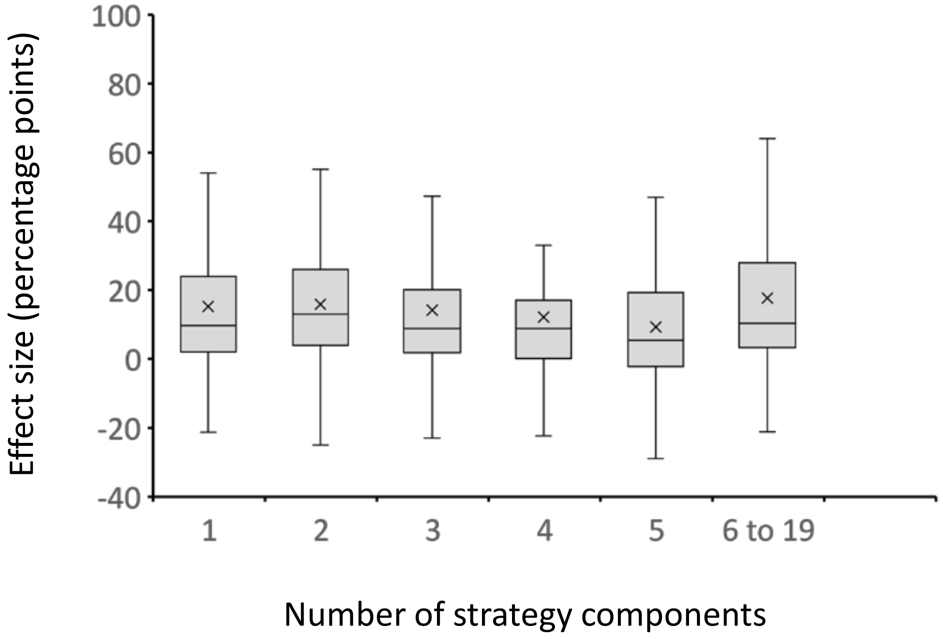

Footnotes

1. In these box-and-whisker plots, the "X" represents the mean effect size for each category of the number of strategy components, the line inside the box represents the median effect size, the boundaries of the box represent the 25th (lower) and 75th (upper) percentiles, the lower whisker represents the minimum, and the upper whisker represents the maximum.

2. Analysis of 1482 effect sizes

3. There were 374 effect sizes for the 1 component category, 238 effect sizes for the 2 component category, 306 effect sizes for the 3 component category, 124 effect sizes for the 4 component category, 196 effect sizes for the 5 component category, and 244 effect sizes for the 6 to 19 component category.

4. The number of components in a strategy can be greater than the number of strategy component categories (i.e., the categories used to describe strategies in Table F). For example, the hypothetical strategy of "provision of drugs plus implementation of an essential drug list" has two strategy components. However, as both components are in the "strengthening infrastructure" component category, the strategy (if it were in Table F) would be listed as "strengthening infrastructure only."

Figure C2. Effect size distributions for practice outcomes expressed as a percentage as a function of the number of component categories in the strategy

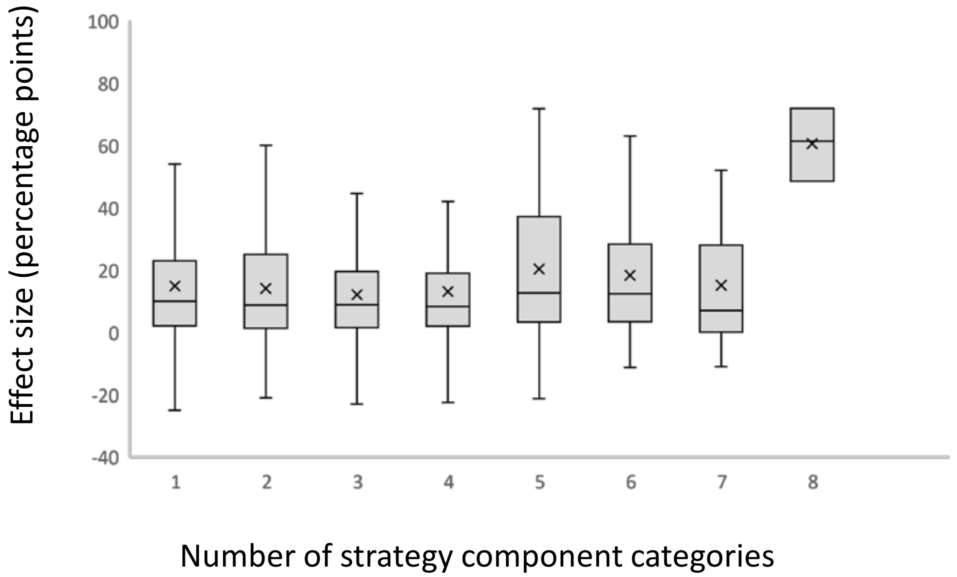

Footnotes

1. In these box-and-whisker plots, the “X” represents the mean effect size for each category of the number of strategy components, the line inside the box represents the median effect size, the boundaries of the box represent the 25th (lower) and 75th (upper) percentiles, the lower whisker represents the minimum, and the upper whisker represents the maximum.

2. Analysis of 1482 effect sizes

3. There were 625 effect sizes for 1 component category, 338 effect sizes for 2 component categories, 234 effect sizes for 3 component categories, 170 effect sizes for 4 component categories, 40 effect sizes for 5 component categories, and 45 effect sizes for 6 component categories, 27 effect sizes for 7 component categories, and 3 effect sizes for 8 component categories.
